# Supplementary material for: Robust genetic codes enhance protein evolvability
Source: PLoS Biol. 2024 May 16;22(5):e3002594. doi: 10.1371/journal.pbio.3002594 (PMC11098591; doi:10.1371/journal.pbio.3002594)
Supplement: S1 Text — (PDF) [file pbio.3002594.s001.pdf]

S1 Text: Supplementary materials

for

Robust genetic codes enhance protein evolvability

Hana Rozhoňová[1,2,*], Carlos Martí-Gómez[3], David M. McCandlish[3], and Joshua L. Payne[1,2,*]

[1]Institute of Integrative Biology, ETH Zürich, Zürich, Switzerland
[2]Swiss Institute of Bioinformatics, Lausanne, Switzerland
[3]Simons Center for Quantitative Biology, Cold Spring Harbor Laboratory, Cold Spring Harbor, NY, USA
[*]hana.rozhonova@env.ethz.ch (HR), joshua.payne@env.ethz.ch (JLP)

Contents

Supplementary figures **3**

Supplementary tables **22**

1 Artificial inflation of GB1 landscape ruggedness **36**

2 Analysis of physicochemical properties from the Aaindex database **38**

3 Data set-specific definition of code robustness **41**

4 Restricted amino acid permutation codes **46**

5 Random codon assignment codes **48**

6 Landscape dimensionality **50**

7 Causes of the correlation between code robustness and mean fitness reached by greedy adaptive walks **53**

7.1 Greedy walks in the DHFR landscape 54

8 Weak mutation adaptive walks **56**

9 Fitness landscape visualizations **58**

9.1 Standard genetic code 58

9.2 Robust genetic codes 59

9.3 Non-robust genetic codes 59

10 Epistasis under the Ostrov codes **61**

11 Examples of Ostrov codes promoting or diminishing evolvability **64**

Supplementary figures

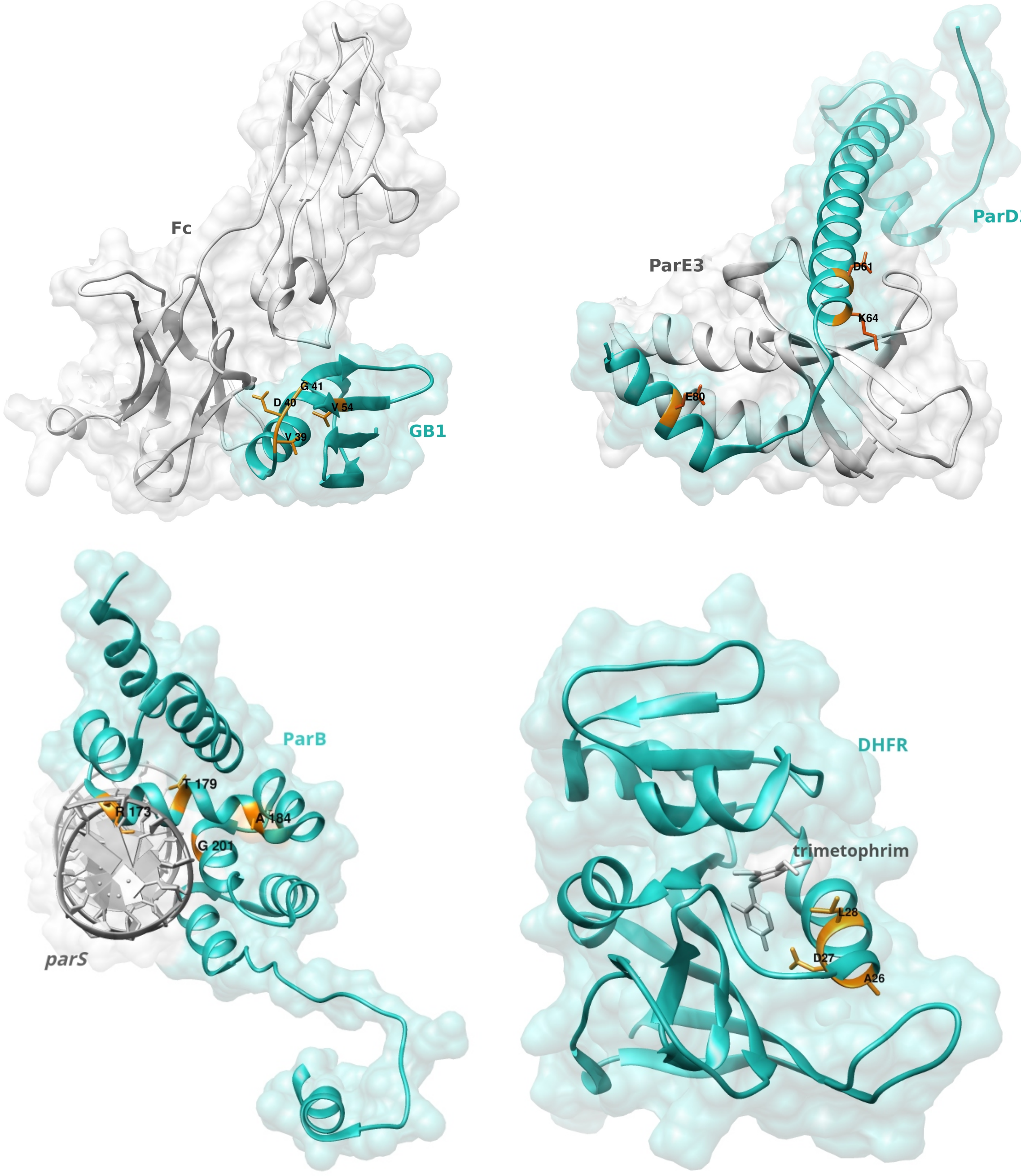

Figure A: Structures of the (top left) GB1, (top right) ParD3, (bottom left) ParB, and (bottom right) DHFR proteins, in complex with their corresponding ligands (Fc domain of IgG for GB1; ParE3 for ParD3; *parS* DNA motif for ParB; antibiotic trimetophrim for DHFR). The residues used to build the adaptive landscapes are highlighted in orange. PDB IDs: 1FCC for GB1, 5CEG for ParD3, 6S6H for ParB, 6XG5 for DHFR.

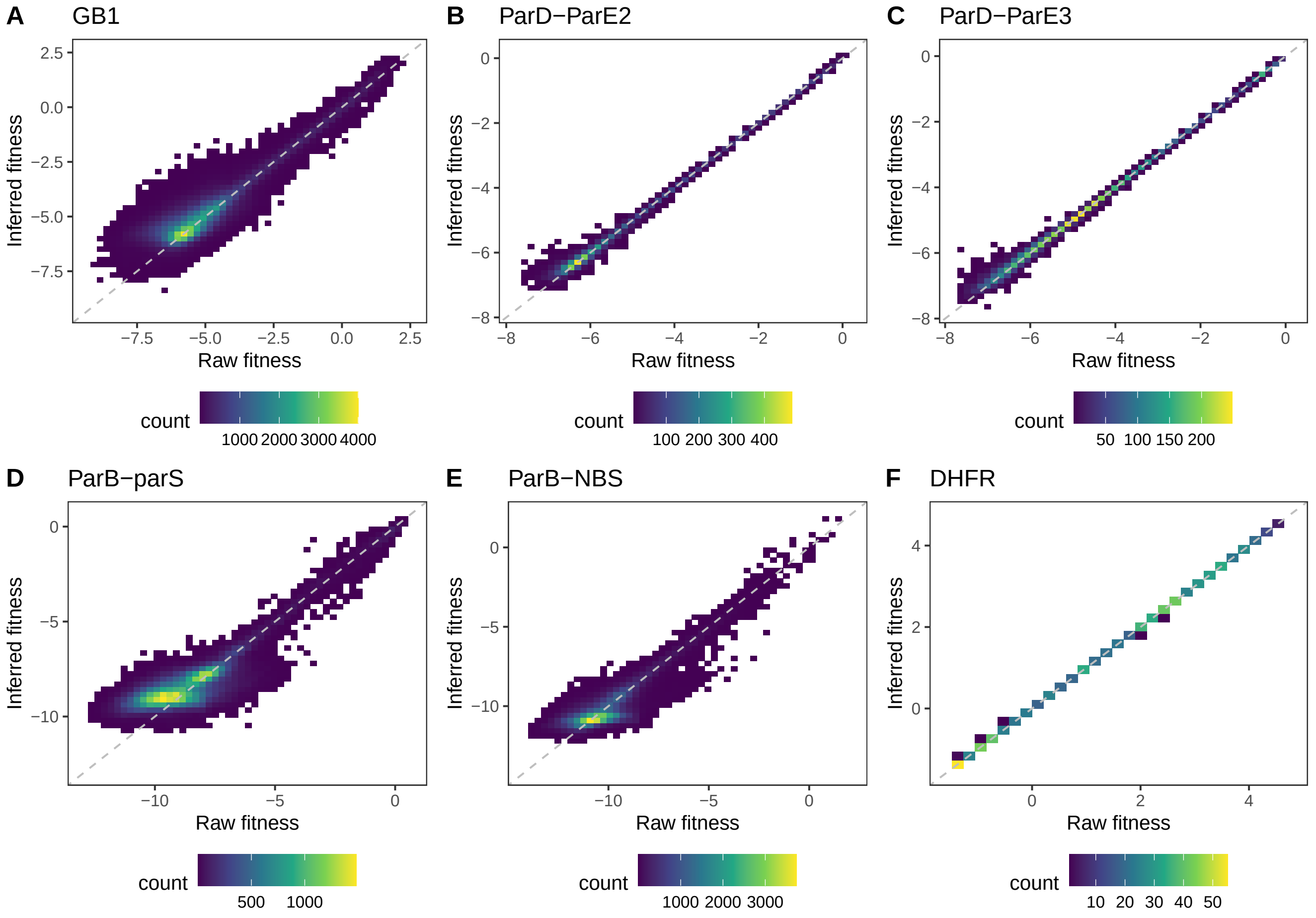

Figure B: Density plot of the raw fitness values and the fitness values inferred using empirical variance component regression (Methods) [1] for the (A) GB1, (B) ParD-ParE2, (C) ParD-ParE3, (D) ParB-*parS*, (E) ParB-*NBS*, and (F) DHFR data sets. For DHFR, only the 827 functional variants are shown. The data and code required to generate this Figure can be found at https://zenodo.org/records/10677993.

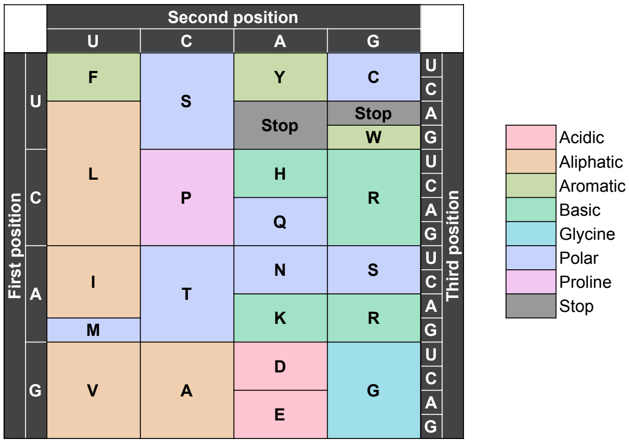

Figure C: Codon table for the standard genetic code, with codons colored based on the physicochemical property of the encoded amino acid. Classification into physicochemical properties taken from ref. [2].

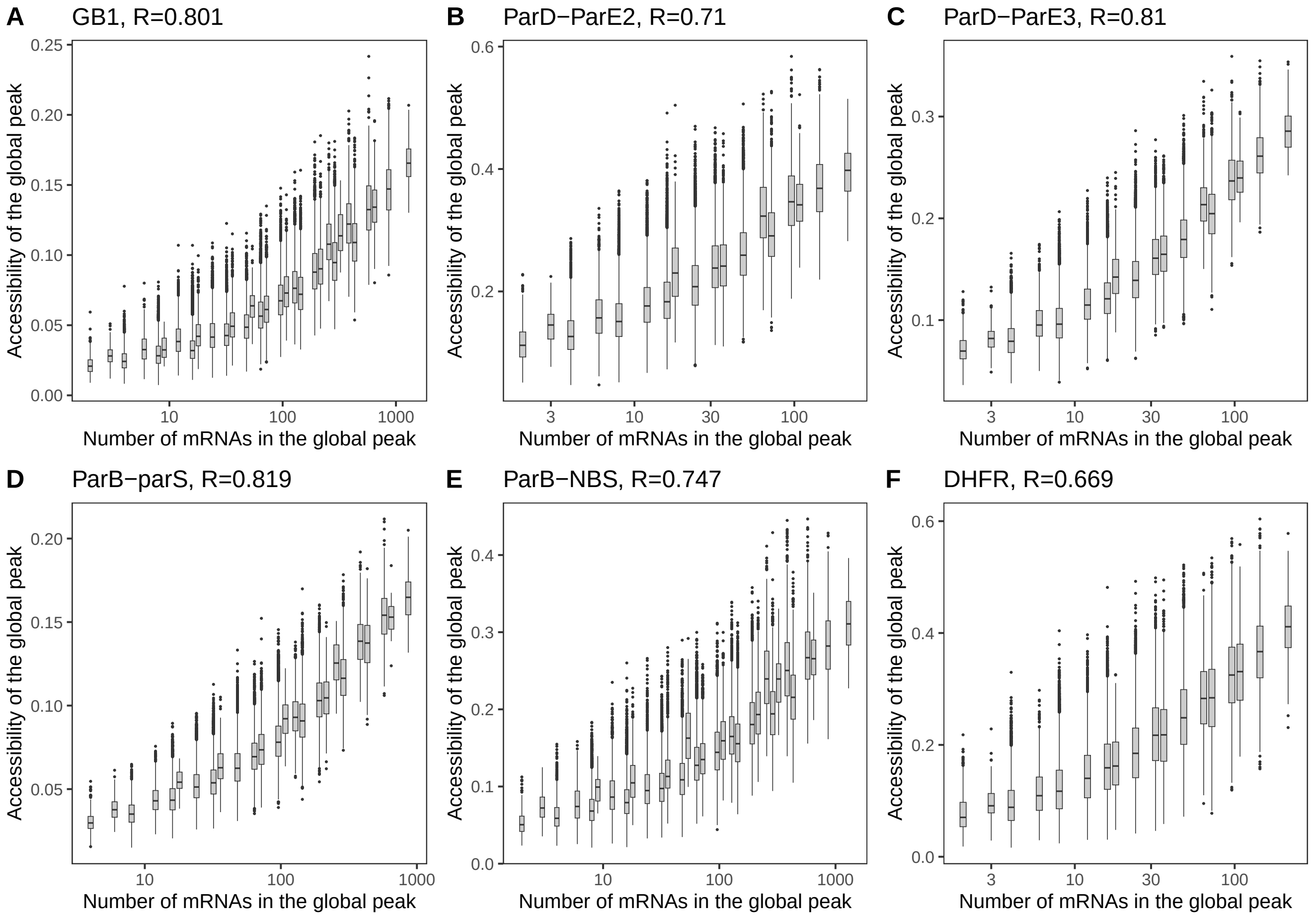

Figure D: Accessibility of the global peak in relation to its size for the (A) GB1, (B) ParD-ParE2, (C) ParD-ParE3, (D) ParB-*parS*, (E) ParB-*NBS*, and (F) DHFR landscapes. Mutational accessibility is measured as the proportion of randomly chosen direct paths to the global peak that are accessible, meaning that fitness increases monotonically along the path. Peak size is measured as the number of mRNA sequences encoding the protein with the maximum fitness value. Data pertain to the 100,000 amino acid permutation codes. Values of the Pearson's correlation are shown on the top of each plot. The box-and-whisker plots show the median, 25th and 75th percentile. The upper whisker extends from the top of the box to the largest value no further than 1.5-times the inter-quartile range, the lower whisker extends from the bottom of the box to the smallest value no further than 1.5-time the inter-quartile range. Data beyond the end of the whiskers are plotted individually. The data and code required to generate this Figure can be found at https://zenodo.org/records/10677993.

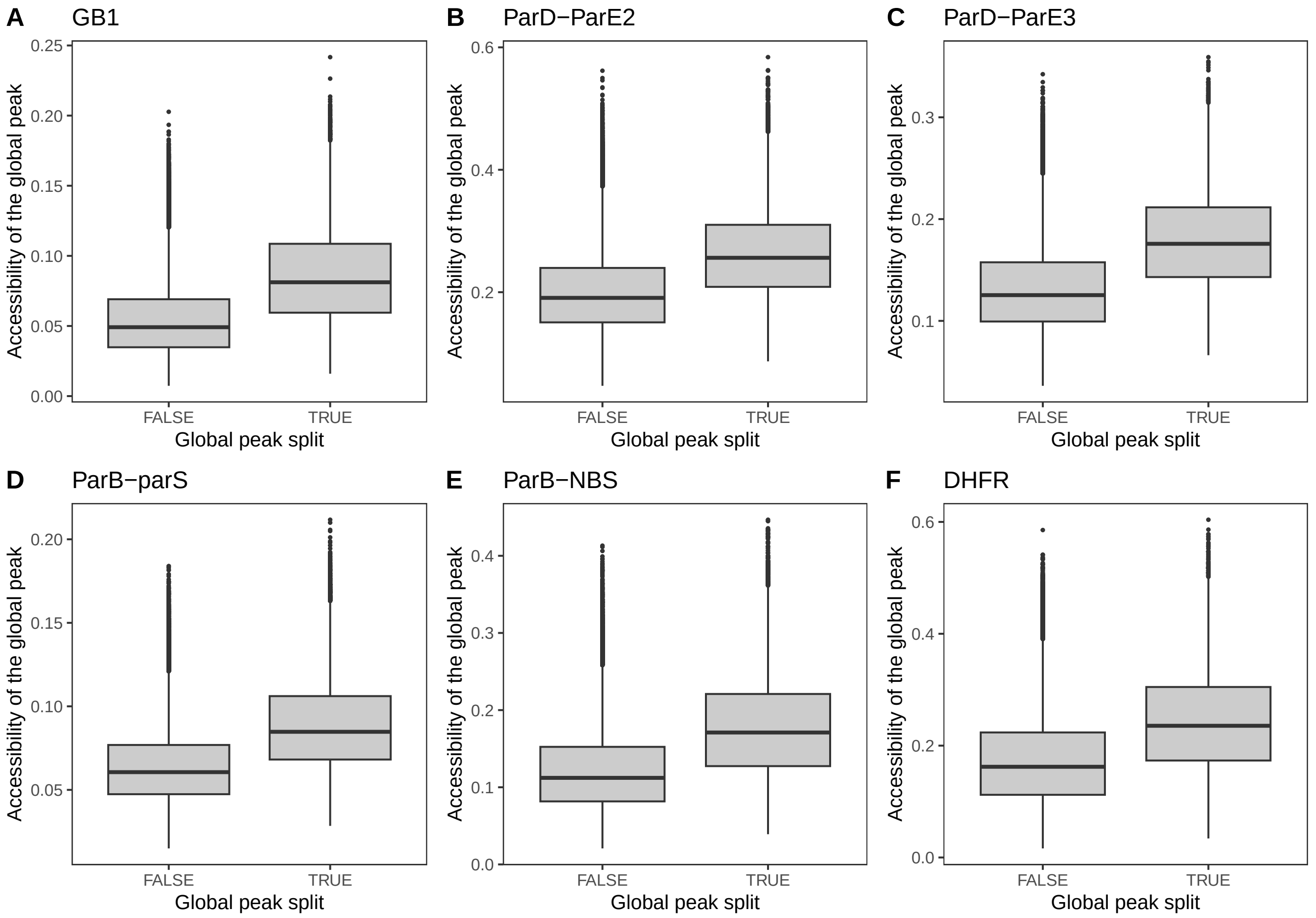

Figure E: Accessibility of the global peak in relation to whether it forms a single connected region in genotype space for the (A) GB1, (B) ParD-ParE2, (C) ParD-ParE3, (D) ParB-*parS*, (E) ParB-*NBS*, and (F) DHFR landscapes. The global peak occupies disconnected regions of genotype space when an amino acid in the protein sequence with the highest fitness value is encoded by the split codon block. Data pertain to the 100,000 amino acid permutation codes. Meaning of box-and-whisker plots defined in Fig. D. The data and code required to generate this Figure can be found at https://zenodo.org/records/10677993.

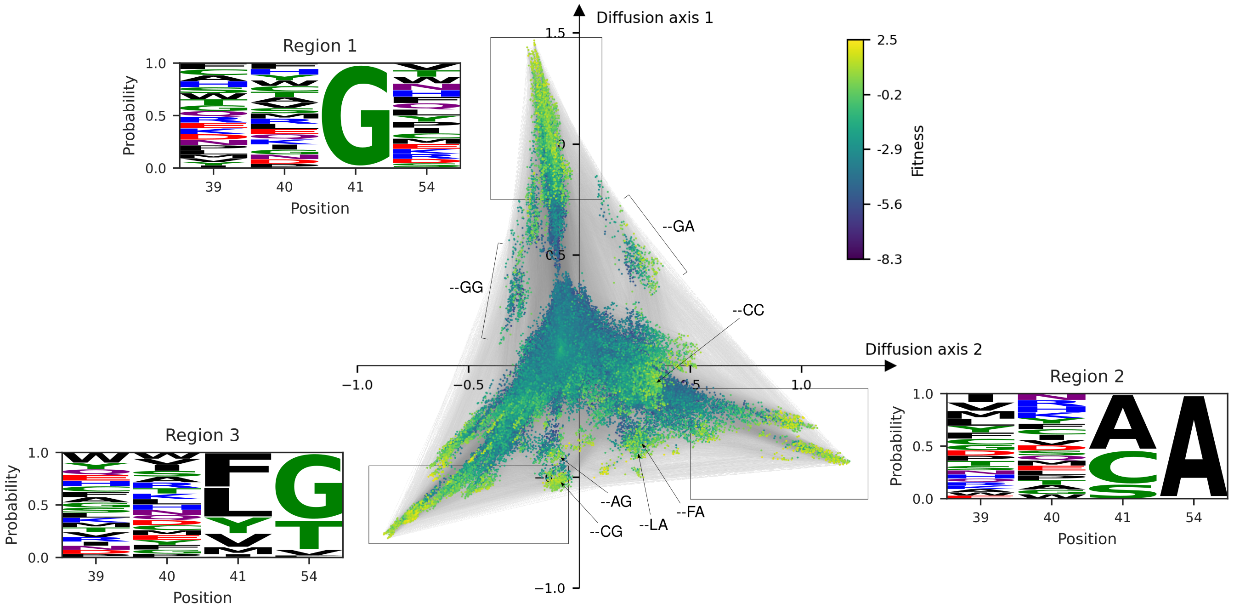

Figure F: Visualization of the GB1 fitness landscape at the protein sequence level. Vertices represent 4-amino acid sequences and edges connect vertices if their corresponding sequences differ by a single amino acid substitution. Vertex color represents protein fitness. Vertices are placed at the coordinates along the diffusion axes, which at a technical level are defined by the subdominant eigenvectors of the rate matrix describing the weak mutation dynamics [3] (see Methods for details), and squared distances have units of time measured in expected amino acid substitutions per site under neutrality. Boxes are drawn around the 3 main regions of functional sequences and frequency logos of those subsets of variants are drawn next to them. The data and code required to generate this Figure can be found in https://github.com/parizkh/rewired_codes_landscapes/tree/main/GB1/05_landscape_visualizations.

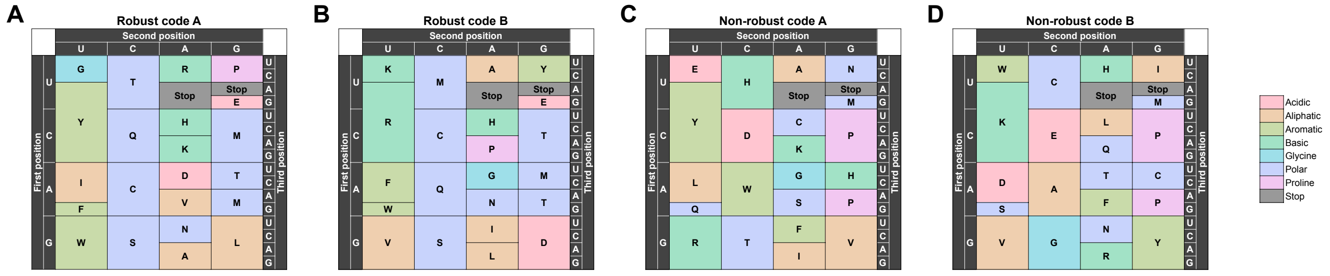

Figure G: The amino acid permutation codes with the (A, B) highest and (C, D) lowest level of robustness. Codons are colored based on the physicochemical properties of the encoded amino acid, following Pines et al. [2].

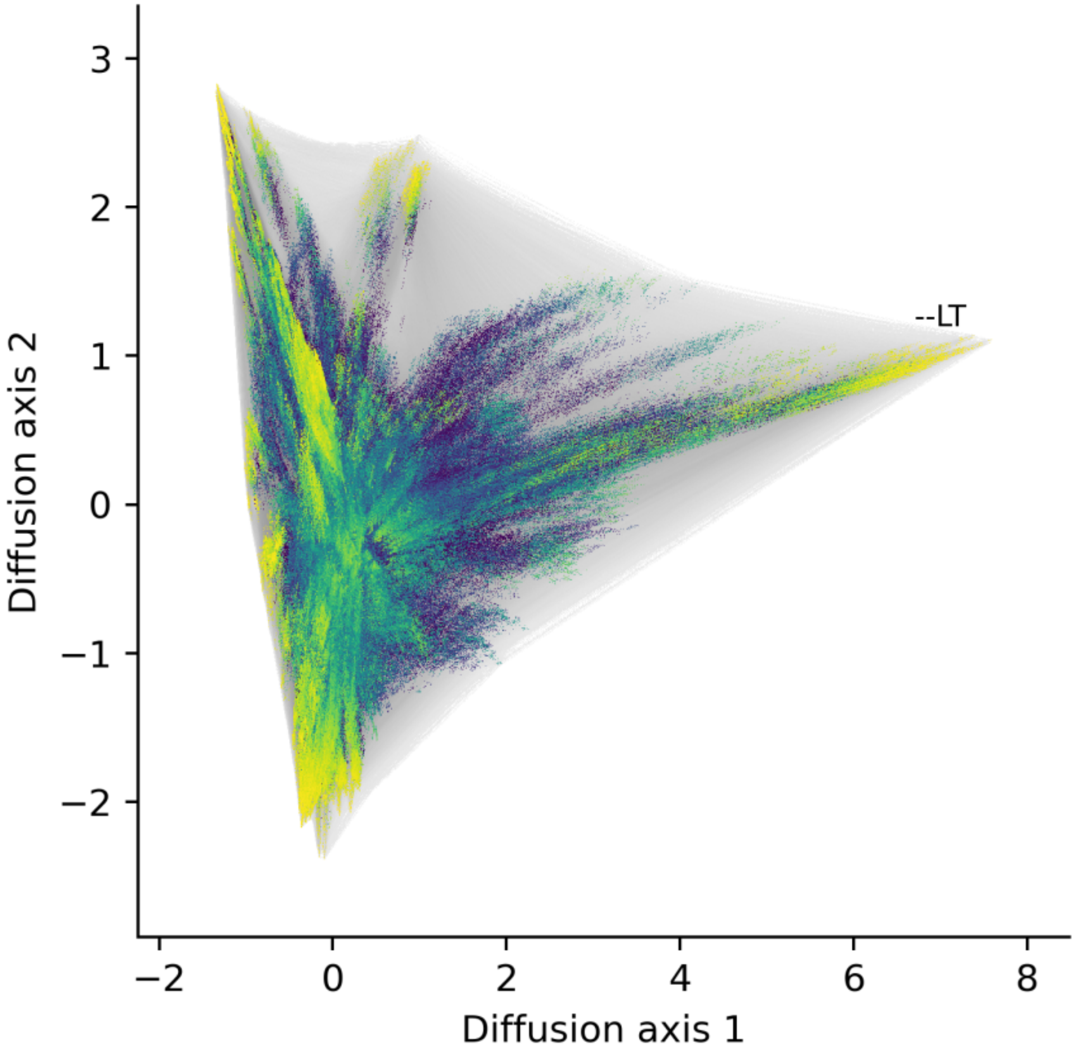

Figure H: Visualization of the GB1 landscape under Robust Code B. The cluster of 41L-54T variants are indicated, which are separated from the rest of the landscape along Diffusion Axis 1. See Fig. 4 for further information on the layout, as well as the meaning of vertices and edges, and see Fig. 4C for an additional visualization of this landscape along Diffusion Axis 3. The data and code required to generate this Figure can be found in https://github.com/parizkh/rewired_codes_landscapes/tree/main/GB1/05_landscape_visualizations.

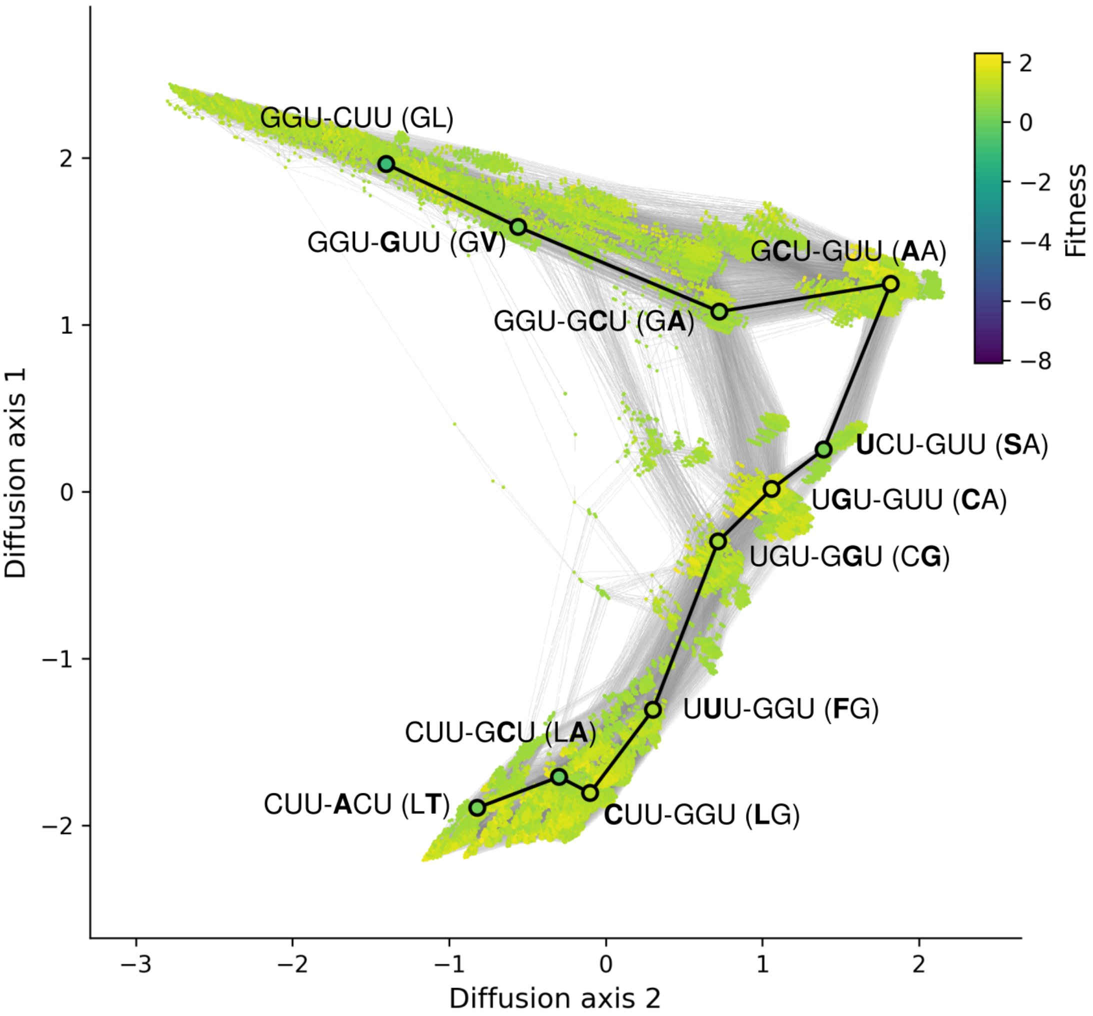

Figure I: Visualization of the genotype network of high-fitness variants in the GB1 landscape. The highlighted mutational path connects an mRNA sequence for 41G-54L to an mRNA sequence for 41L-54T via a series of intermediate mRNA sequences that are also part of the genotype network (i.e. that are among the 1% most fit sequences). Each highlighted edge corresponds to a non-synonymous point mutation, see Fig. 4 for further information on the layout. The modified nucleotide and amino acid are shown in bold at each step in the path. Color bar indicates protein fitness. The data and code required to generate this Figure can be found in https://github.com/parizkh/rewired_codes_landscapes/tree/main/GB1/05_landscape_visualizations.

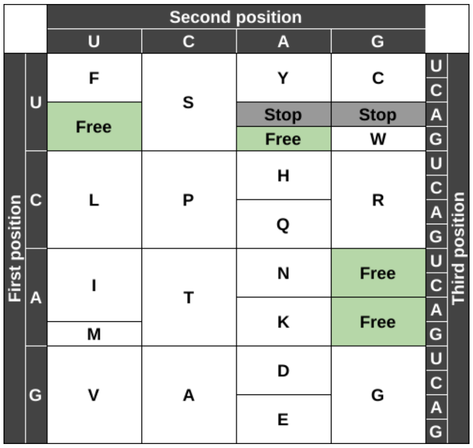

Figure J: The codon table of the 57-codon *E. coli* genome [4], highlighting the four codon blocks that have been freed for reassignment.

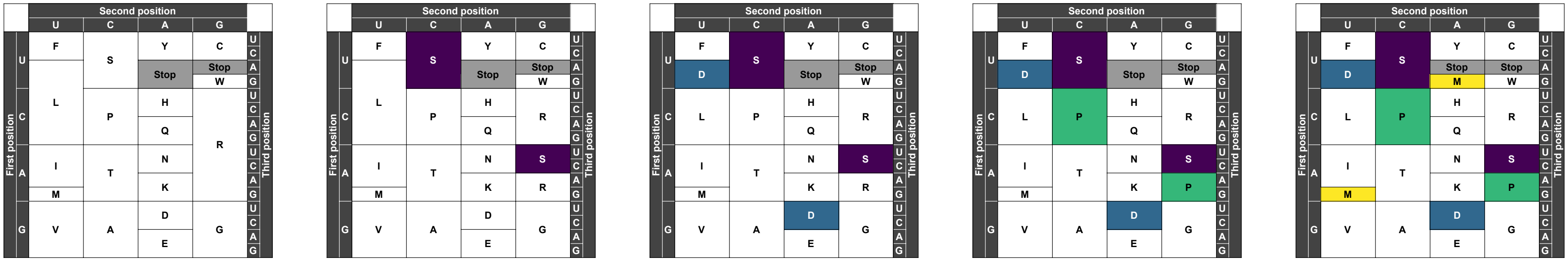

Figure K: Examples of Ostrov codes with (left) 0 to (right) 4 split codon blocks, which are highlighted in colors.

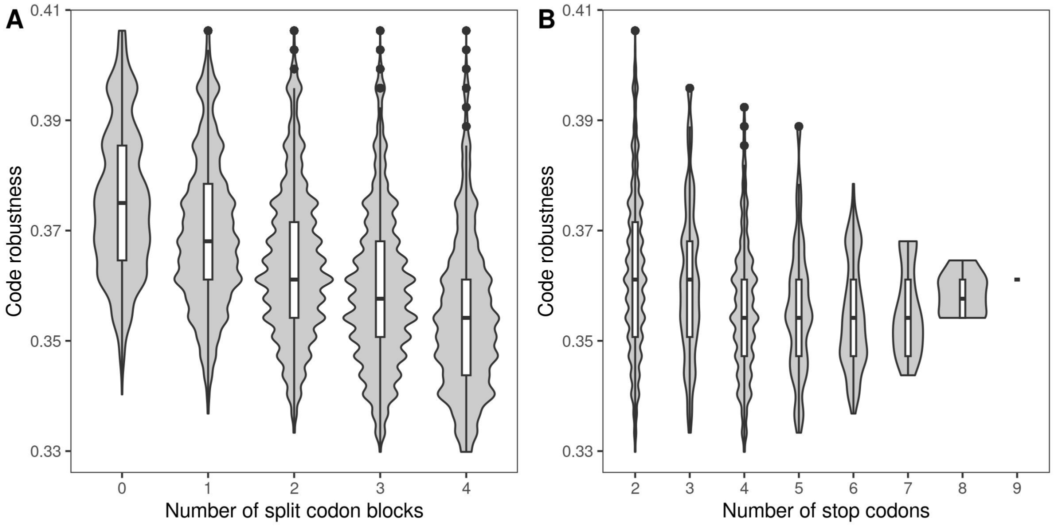

Figure L: Violin plots of code robustness in relation to (A) the number of split codon blocks and (B) the number of stop codons in the 194,481 Ostrov codes. The violin plots show the distribution and the box-and-whisker plots the median, 25th and 75th percentile of code robustness (see Fig D in S1 Text for details). The data and code required to generate this Figure can be found at https://zenodo.org/records/10677993.

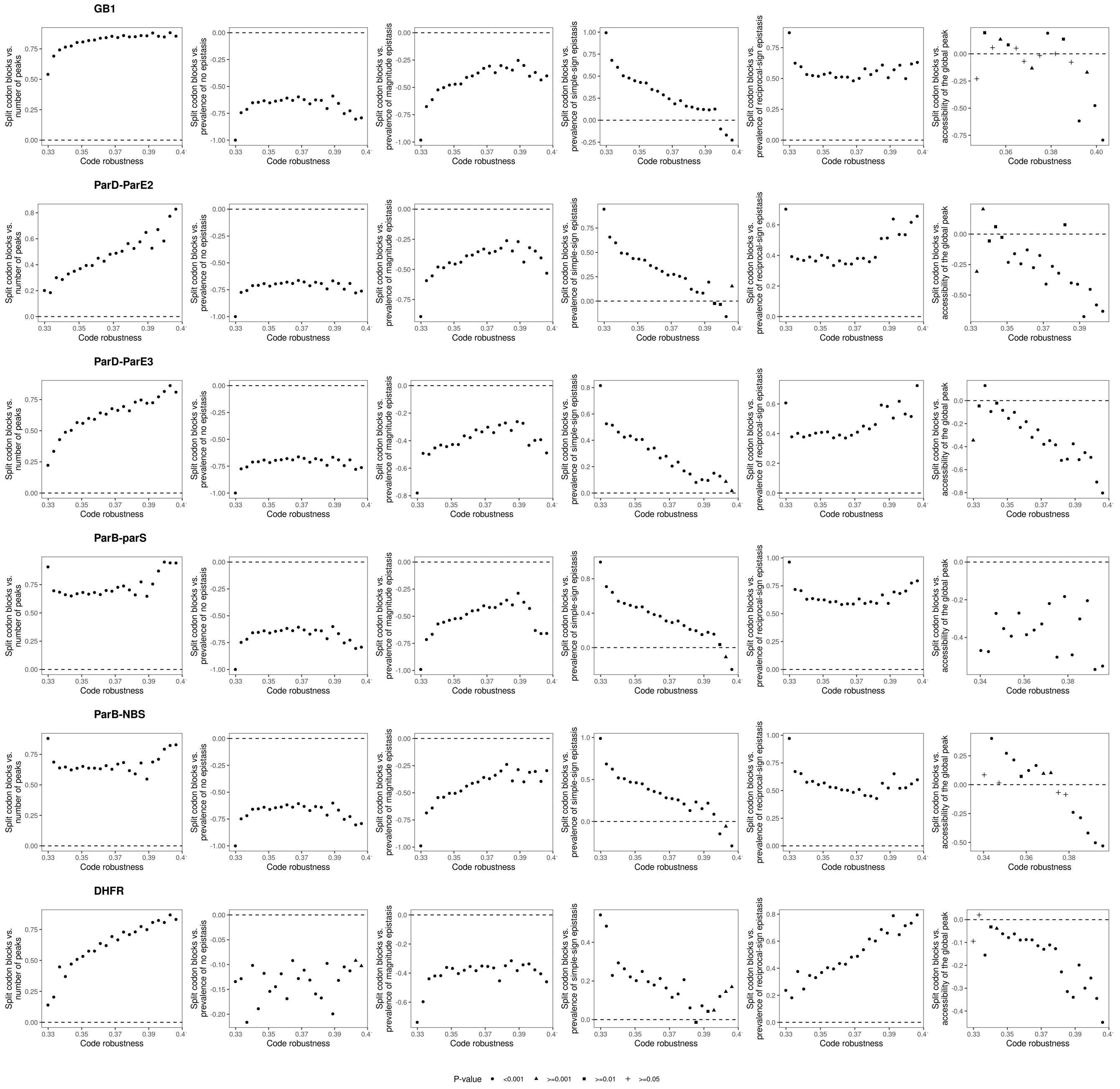

Figure M: Pearson's correlation between various measures of landscape ruggedness and the number of split codon blocks, for Ostrov codes with a given value of robustness. The shape of the points denotes the p-value of the correlation coefficient, corrected for testing multiple hypotheses (legend). The magnitude, simple-sign, and reciprocal-sign epistasis results are based on prevalence of a given type of epistasis relative to all epistatic squares. Data for global peak accessibility are based on the subset of Ostrov codes that preserve the size of the global peak and the global peak occupies a single connected region in genotype space. Dashed horizontal lines indicates no correlation. The data and code required to generate this Figure can be found at https://zenodo.org/records/10677993.

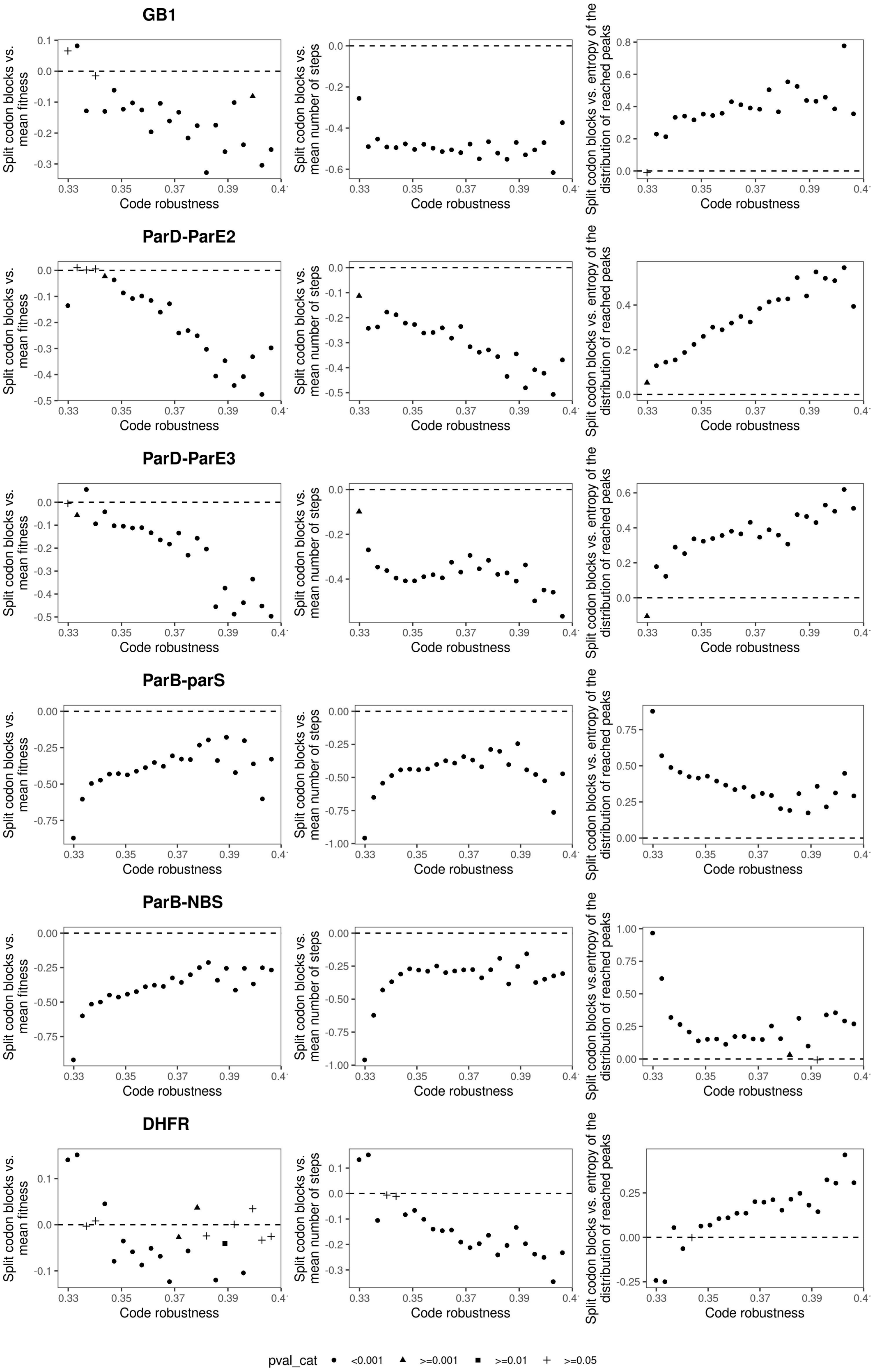

Figure N: Correlation between various outcomes of the greedy adaptive walks and the number of split codon blocks, for Ostrov codes with a given value of robustness. The shape of the points denotes the p-value of the correlation coefficient, corrected for testing multiple hypotheses (legend). Dashed horizontal lines indicates no correlation. The data and code required to generate this Figure can be found at https://zenodo.org/records/10677993.

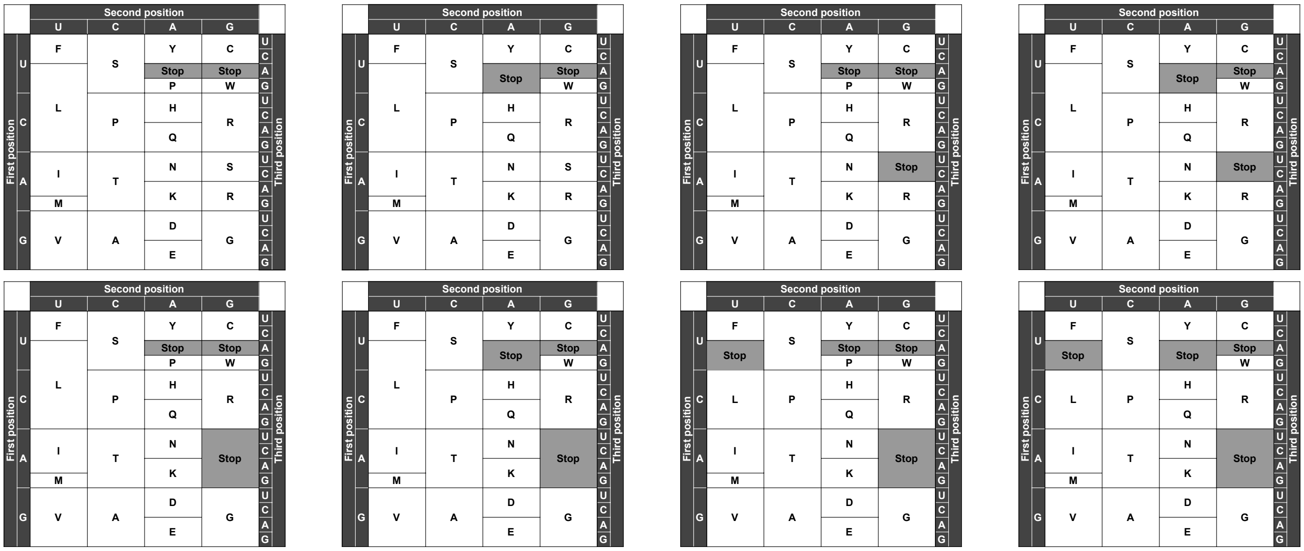

Figure O: Examples of Ostrov codes with (top left) 2 to (bottom right) 9 stop codons, which are highlighted in grey.

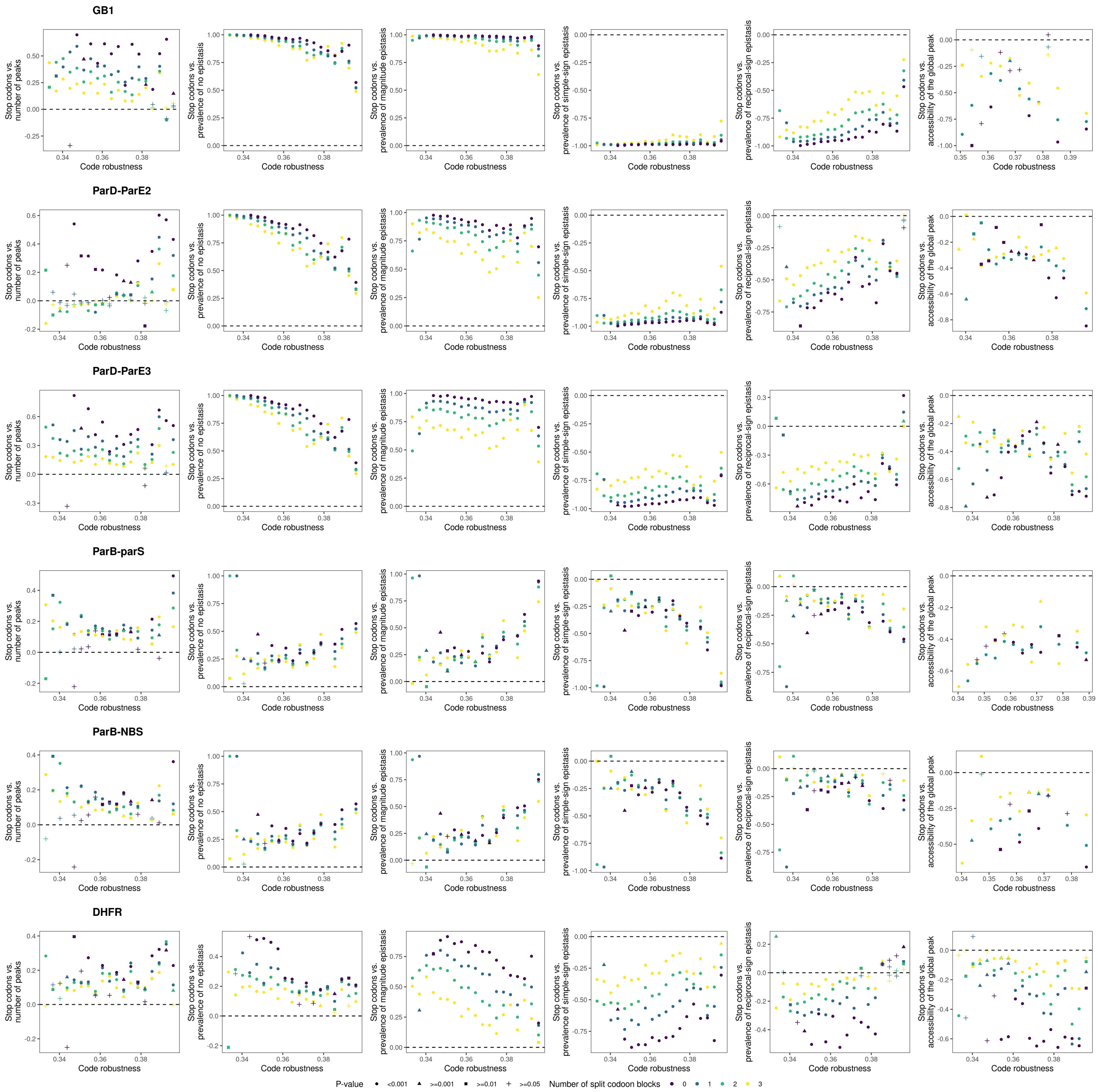

Figure P: Correlation between various measures of landscape ruggedness and the number of stop codons, for Ostrov codes with a given value of robustness. The shape of the points denotes the p-value of the correlation coefficient, corrected for testing multiple hypotheses, and the color of the points denotes the number of split codon blocks (legend). The magnitude, simple-sign, and reciprocal-sign epistasis results are based on prevalence of a given type of epistasis relative to all epistatic squares. Data for global peak accessibility are based on the subset of Ostrov codes that preserve the size of the global peak and the global peak occupies a single connected region in genotype space. Dashed horizontal lines indicates no correlation. See Section 10 in S1 Text for further details regarding epistasis. The data and code required to generate this Figure can be found at https://zenodo.org/records/10677993.

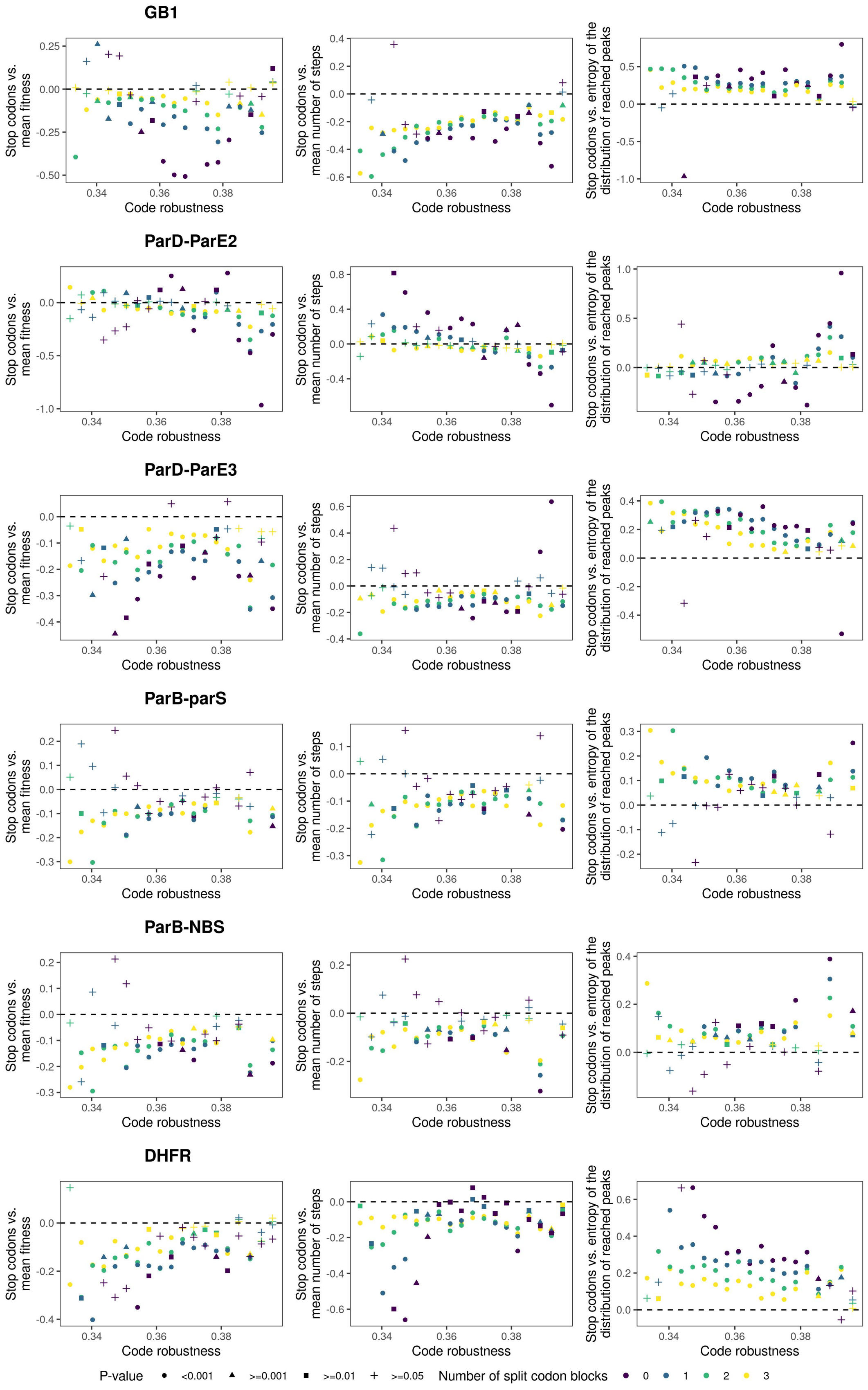

Figure Q: Correlation between various outcomes of the greedy adaptive walks and the number of stop codons, for Ostrov codes with a given value of robustness. The shape of the points denotes the p-value of the correlation coefficient, corrected for testing multiple hypotheses, and the color of the points denotes the number of split codon blocks (legend). The data and code required to generate this Figure can be found at https://zenodo.org/records/10677993.

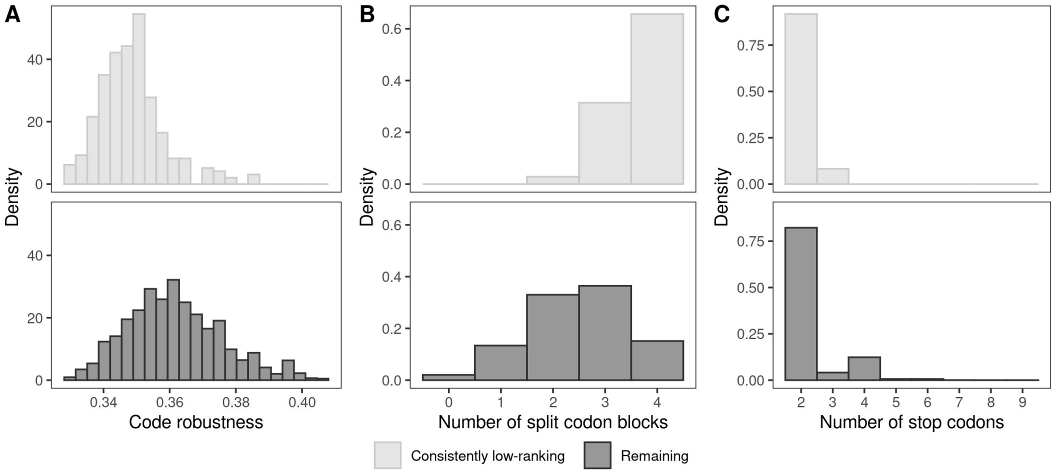

Figure R: Comparison of the properties of the 280 consistently low-ranking codes with the remaining 194,201 codes, in terms of (A) code robustness, (B) number of split codon blocks, and (C) number of stop codons. The data and code required to generate this Figure can be found at https://zenodo.org/records/10677993.

Supplementary tables

	Proportion of reciprocal-sign epistasis	Accessibility of the global peak
**GB1**		
Number of adaptive peaks	0.459	0.237
Proportion of reciprocal-sign epistasis		0.160
**ParD-ParE2**		
Number of adaptive peaks	0.404	0.284
Proportion of reciprocal-sign epistasis		0.384
**ParD-ParE3**		
Number of adaptive peaks	0.373	0.201
Proportion of reciprocal-sign epistasis		0.057
**ParB-*parS***		
Number of adaptive peaks	0.515	0.429
Proportion of reciprocal-sign epistasis		0.511
**ParB-*NBS***		
Number of adaptive peaks	0.624	0.496
Proportion of reciprocal-sign epistasis		0.691
**DHFR**		
Number of adaptive peaks	0.229	0.241
Proportion of reciprocal-sign epistasis		0.078

Table A: Correlations of the individual measures of ruggedness with each other, for the six data sets.

	GB1	ParD-ParE2	ParD-ParE3	ParB-*parS*	ParB-*NBS*	DHFR
Number of peaks	-0.144	-0.119	-0.0346	-0.049	-0.103	-0.084
Prevalence of no epistasis	-	-	-	-	-	0.128
Prevalence of magnitude epistasis	0.247	0.219	0.118	0.097	0.148	-0.128; 0.102
Prevalence of simple-sign epistasis	-0.125	-0.125	-0.0444	-0.020	-0.081	-0.104; -0.043
Prevalence of reciprocal-sign epistasis	-0.277	-0.262	-0.191	-0.137	-0.209	-0.168; -0.200
Accessibility of the global peak	0.024	0.0052 $p = 0.099$	-0.151	0.097	0.035	0.078
Accessibility of the global peak (codes preserving the size of the global peak)	0.086	0.0920	-0.0846	0.065	0.097	0.111

Table B: Correlation of various measures of landscape ruggedness with code robustness for amino acid permutation codes. All correlations are statistically significant, unless stated otherwise. Results for the prevalence of no epistasis are shown only for the DHFR landscape, because all amino acid permutation codes have the same proportion of squares with no epistasis in the remaining five landscapes: As the fitness values of all protein variants are distinct in these five ladscapes, only squares that involve at least two synonymous mutations exhibit no epistasis, and because all 100,000 amino acid permutation codes have the same block structure, the prevalence of such squares is the same for all of them. This is not true in the DHFR landscape, because of the non-functional variants, which are all assigned the same fitness value. For magnitude, simple-sign, and reciprocal-sign epistasis in the DHFR landscape, the first number is the correlation between the absolute prevalence of the corresponding type of epistasis and code robustness, while the second one is the correlation between code robustness and the prevalence of a given type of epistasis among epistatic squares only. The size of the subset of codes that preserve the size of the global peak and under which the global peak forms a single connected region in the genotype space (last line of the table) is $n = 3,769$, GB1; $n = 12,059$, ParD-ParE2; $n = 6,781$, ParD-ParE3; $n = 2,301$, ParB-*parS*; $n = 3,257$, ParB-*NBS*; $n = 6,852$, DHFR.

		GB1	ParD-ParE2	ParD-ParE3	ParB-*parS*	ParB-*NBS*	DHFR
Ruggedness	Number of peaks	0.00037	0.326	0.570	0.0229	0.046	0.205
	Prevalence of no epistasis	-	-	-	-	-	0.892
	Prevalence of magnitude epistasis	0.975	0.664	0.341	0.750	0.973	0.097; 0.240
	Prevalence of simple-sign epistasis	0.123	0.548	0.604	0.481	0.103	0.323; 0.832
	Prevalence of reciprocal-sign epistasis	0.0169	0.189	0.676	0.150	0.00404	0.154; 0.317
	Accessibility of the global peak	0.538; 0.864	0.609; 0.610	0.117; 0.643	0.688; 0.681	0.719; 0.590	0.622; 0.983
Greedy walks	Mean fitness	0.812; 0.857	0.961; 0.970	0.647; 0.864	0.953; 0.967	0.988; 0.975	0.186; 0.277
	Mean number of steps	0.936; 0.903	0.993; 0.995	0.928; 0.844	0.415; 0.282	0.995; 0.998	0.385; 0.980
	Entropy of the distribution of reached peaks	0.00304; 0.0024	0.00034; 0.00041	0.189; 0.084	0.0139; 0.0148	0.0024; 0.00675	0.490; 1.000
Weak mutation walks	Mean fitness after 500 mutations, $N = 10$	0.781; 0.839	0.756; 0.749	0.447; 0.807	0.975; 0.990	0.951; 0.943	0.071; 0.123
	Mean fitness after 500 mutations, $N = 100$	0.832; 0.884	0.795; 0.802	0.385; 0.748	0.960; 0.983	0.852; 0.738	0.171; 0.277
	Mean fitness after 500 mutations, $N = 10,000$	0.842; 0.893	0.797; 0.806	0.404; 0.775	0.961; 0.980	0.824; 0.662	0.257; 0.410
	Mean fitness after 500 mutations, $N = 1,000,000$	0.842; 0.894	0.796; 0.805	0.404; 0.775	0.961; 0.980	0.824; 0.660	0.255; 0.408

Table C: Proportion of the amino acid permutation codes with lower or equal value of a given characteristic, as compared to the standard genetic code. Results for no epistasis shown only for DHFR, as it is the only landscape in which proportion of squares exhibiting no epistasis differs among amino acid permutation codes (see description of Table B in S1 Text). For magnitude, simple-sign, and reciprocal-sign epistasis in the DHFR landscape, the first number gives the proportion for the absolute prevalence of the corresponding type of epistasis, while the second one for the prevalence of a given type of epistasis among epistatic squares only. For accessibility of the global peak and the greedy and weak mutation adaptive walks, two proportions are shown: the first one is the proportion of such codes in the whole data set of 100,000 amino acid permutation codes, the second is based on the subset of codes that preserve the size of the global peak and under which the global peak forms a single connected region in the genotype space ($n = 3,769$, GB1; $n = 12,059$, ParD-ParE2; $n = 6,781$, ParD-ParE3; $n = 2,301$, ParB-*parS*; $n = 3,257$, ParB-*NBS*; $n = 6,852$, DHFR). Note that in the ParB-*parS* data set, the standard genetic code is not a member of this set, as the global peak sequence in this landscape is RCWS and S is encoded by the split codon block in the standard genetic code.

	GB1	ParD-ParE2	ParD-ParE3	ParB-*parS*	ParB-*NBS*	DHFR
Mean fitness	0.107; 0.130	0.121; 0.183	−0.00422 ($p = 0.182$); 0.092	−0.0118; −0.0776	0.0731; 0.0468	−0.096; −0.082
Mean number of steps	0.192; 0.239	0.179; 0.170	0.157; 0.124	0.0454; 0.0654	0.0723; 0.0633	−0.002 ($p = 0.448$); −0.001 ($p = 0.918$)
Entropy of the distribution of reached peaks	−0.147; −0.203	−0.172; −0.213	−0.0702; −0.123	−0.0730; −0.0257 ($p = 0.218$)	−0.100; −0.0652	−0.065; −0.048

Table D: Correlation of code robustness with the outcomes of greedy adaptive walks for amino acid permutation codes. The first number is the correlation in the set of 100,000 amino acid permutation codes, the second the correlation in the subset of codes that preserve the size of the global peak and under which the global peak is formed by a single connected region in the sequence space ($n = 3,769$, GB1; $n = 12,059$, ParD-ParE2; $n = 6,781$, ParD-ParE3; $n = 2,301$, ParB-*parS*; $n = 3,257$, ParB-*NBS*; $n = 6,852$, DHFR). All correlations are statistically significant, unless specified otherwise (p-values in parentheses).

Landscape	R	p-value
GB1	0.285	$p < 2.2 \cdot 10^{-16}$
ParD-ParE2	0.377	$p < 2.2 \cdot 10^{-16}$
ParD-ParE3	0.352	$p < 2.2 \cdot 10^{-16}$
ParB-*parS*	0.389	$p < 2.2 \cdot 10^{-16}$
ParB-*NBS*	0.614	$p < 2.2 \cdot 10^{-16}$
DHFR	0.219	$p < 2.2 \cdot 10^{-16}$

Table E: Correlation between the size of the global peak and mean fitness reached by the greedy adaptive walks in the set of 100,000 amino acid permutation codes.

	GB1	ParD-ParE2	ParD-ParE3	ParB-*parS*	ParB-*NBS*	DHFR
Number of peaks	−0.412	−0.359	−0.300	−0.082	−0.145	−0.188
Prevalence of no epistasis	0.072	0.165	0.165	0.081	0.081	0.290
Prevalence of magnitude epistasis	−0.117; 0.0066, $p = 0.0035$	0.005, $p = 0.029$; 0.152	−0.132; 0.056	−0.265; −0.073	−0.141; −0.005 ($p = 0.036$)	−0.286; 0.193
Prevalence of simple-sign epistasis	0.010; 0.087	−0.080; −0.010	−0.065; 0.005, $p = 0.018$	0.038; 0.140	0.013; 0.097	−0.298; 0.045
Prevalence of reciprocal-sign epistasis	−0.180; −0.225	−0.309; −0.311	−0.198; −0.180	−0.102; −0.101	−0.176; −0.209	−0.357; −0.459
Accessibility of the global peak	0.142	−0.082	−0.230	0.234	0.197	0.150
Accessibility of the global peak (codes preserving the size of the global peak)	0.256	0.006, $p = 0.403$	0.039	0.248	0.507	0.387

Table F: Correlation of various measures of landscape ruggedness with code robustness for the Ostrov codes. All correlations are statistically significant, unless stated otherwise. For magnitude, simple-sign, and reciprocal-sign epistasis, the first number is the correlation between the absolute prevalence of the corresponding type of epistasis and code robustness, while the second one is the correlation between code robustness and the prevalence of a given type of epistasis among epistatic squares only (i.e., in the second case, squares with no epistasis are discarded).

	GB1	ParD-ParE2	ParD-ParE3	ParB-*parS*	ParB-*NBS*	DHFR
Mean fitness	0.388; 0.357	0.208; 0.331	−0.076; 0.277	−0.134; −0.038	−0.018; 0.175	−0.313; −0.257
Mean number of steps	0.359; 0.289	0.266; 0.180	0.261; 0.170	−0.083; −0.018 ($p = 0.032$)	0.096; 0.271	−0.133; 0.064
Entropy of the distribution of reached peaks	−0.264; −0.183	−0.151; −0.220	0.040; −0.205	0.097; 0.032	−0.053; −0.151	−0.078; −0.214

Table G: Correlation of code robustness with the outcomes of greedy adaptive walks for the Ostrov codes. The first number is the correlation in the set of 100,000 amino acid permutation codes, the second the correlation in the subset of codes that preserve the size of the global peak and under which the global peak is formed by a single connected region in the sequence space. All correlations are statistically significant, unless stated otherwise.

	GB1	ParD-ParE2	ParD-ParE3	ParB-*parS*	ParB-*NBS*	DHFR
Number of peaks	0.847	0.487	0.657	0.655	0.631	0.627
Prevalence of no epistasis	−0.613	−0.699	−0.699	−0.624	−0.624	−0.225
Prevalence of magnitude epistasis	0.681; −0.380	0.313; −0.414	0.444; −0.364	0.658; −0.402	0.626; −0.392	0.217; −0.401
Prevalence of simple-sign epistasis	0.452; 0.278	0.507; 0.322	0.485; 0.297	0.475; 0.310	0.473; 0.304	0.279; 0.144
Prevalence of reciprocal-sign epistasis	0.614; 0.558	0.568; 0.444	0.576; 0.434	0.633; 0.588	0.618; 0.542	0.321; 0.528
Accessibility of the global peak	−0.094	−0.229	−0.256	−0.387	−0.078	−0.232

Table H: Correlation of various measures of landscape ruggedness with number of split codon blocks for the Ostrov codes. All correlations are statistically significant. The mutational accessibility results pertain to the codes that preserve the size of the global peak and under which the global peak forms a single connected region in the genotype space. For magnitude, simple-sign, and reciprocal-sign epistasis, the first number is the correlation between the absolute prevalence of the corresponding type of epistasis and number of split codon blocks, while the second one is the correlation between number of split codon blocks and the prevalence of a given type of epistasis among epistatic squares only (i.e., in the second case, squares with no epistasis are discarded).

	GB1	ParD-ParE2	ParD-ParE3	ParB-*parS*	ParB-*NBS*	DHFR
Mean fitness	−0.261	−0.214	−0.109	−0.290	−0.347	0.063
Mean number of steps	−0.564	−0.348	−0.431	−0.339	−0.306	−0.081
Entropy of the distribution of reached peaks	0.453	0.360	0.316	0.289	0.174	0.146

Table I: Correlation of number of split codon blocks with the outcomes of greedy adaptive walks for the Ostrov codes. All correlations are statistically significant.

	Number of split codon blocks	GB1	ParD-ParE2	ParD-ParE3	ParB-*parS*	ParB-*NBS*	DHFR
Number of peaks	0	0.460	0.213	0.359	0.066	0.072	0.224
	1	0.390	0.103	0.304	0.089	0.087	0.155
	2	0.330	0.073	0.245	0.110	0.104	0.127
	3	0.249	0.049	0.173	0.124	0.114	0.097
Prevalence of no epistasis	0	0.915	0.773	0.773	0.325	0.325	0.217
	1	0.922	0.789	0.789	0.291	0.291	0.154
	2	0.930	0.804	0.804	0.259	0.259	0.121
	3	0.938	0.822	0.822	0.233	0.233	0.084
Prevalence of magnitude epistasis	0	−0.444; 0.986	0.102; 0.906	0.061; 0.951	−0.174; 0.295	−0.213; 0.276	−0.199; 0.582
	1	−0.429; 0.981	0.154; 0.865	0.071; 0.893	−0.136; 0.270	−0.171; 0.254	−0.143; 0.535
	2	−0.395; 0.970	0.190; 0.806	0.088; 0.812	−0.100; 0.243	−0.132; 0.228	−0.112; 0.454
	3	−0.325; 0.939	0.189; 0.689	0.090; 0.664	−0.073; 0.216	−0.099; 0.198	−0.078; 0.333
Prevalence of simple-sign epistasis	0	−0.983; −0.990	−0.959; −0.969	−0.953; −0.948	−0.330; −0.312	−0.321; −0.297	−0.489; −0.703
	1	−0.983; −0.987	−0.952; −0.953	−0.931; −0.905	−0.303; −0.292	−0.297; −0.281	−0.348; −0.596
	2	−0.982; −0.982	−0.940; −0.926	−0.893; −0.842	−0.276; −0.269	−0.272; −0.260	−0.267; −0.475
	3	−0.977; −0.966	−0.903; −0.860	−0.805; −0.713	−0.250; −0.244	−0.246; −0.236	−0.185; −0.331
Prevalence of reciprocal-sign epistasis	0	−0.928; −0.883	−0.680; −0.495	−0.794; −0.688	−0.283; −0.223	−0.263; −0.184	−0.162; 0.087
	1	−0.918; −0.852	−0.626; −0.446	−0.753; −0.617	−0.244; −0.182	−0.225; −0.147	−0.116; 0.041
	2	−0.898; −0.802	−0.555; −0.386	−0.688; −0.533	−0.208; −0.145	−0.187; −0.111	−0.095; 0.015
	3	−0.843; −0.698	−0.438; −0.296	−0.568; −0.409	−0.175; −0.111	−0.149; −0.074	−0.069; −0.002 ($p = 0.547$)
Accessibility of the global peak	0	−0.488	−0.291	−0.457	−0.420	−0.327	−0.567
	1	−0.427	−0.262	−0.407	−0.448	−0.292	−0.365
	2	−0.330	−0.205	−0.341	−0.415	−0.211	−0.259
	3	-	-	−0.250	-	-	−0.174

Table J: Correlation of various measures of landscape ruggedness with number of stop codons, conditioned on the number of split codon blocks, for the Ostrov codes. Results for 4 split codon blocks not shown because all codes with 4 split codon blocks have 2 stop codons. All correlations are statistically significant. The accessibility of the global peak results pertain to the codes that preserve the size of the global peak and under which the global peak forms a single connected region in the genotype space. For magnitude, simple-sign, and reciprocal-sign epistasis, the first number is the correlation between the absolute prevalence of the corresponding type of epistasis and number of stop codons, while the second one is the correlation between number of stop codons and the prevalence of a given type of epistasis among epistatic squares only (i.e., in the second case, squares with no epistasis are discarded). See Section 10 in S1 Text for the explanation of the epistasis results.

	Number of split codon blocks	GB1	ParD-ParE2	ParD-ParE3	ParB-*parS*	ParB-*NBS*	DHFR
	0	−0.364	−0.147	−0.183	$-6.85 \cdot 10^{-4}$ ($p = 0.966$)	−0.067	0.081
	1	−0.264	−0.128	−0.139	−0.033	−0.078	0.014 ($p = 0.025$)
Mean fitness	2	−0.196	−0.107	−0.104	−0.066	−0.095	−0.012
	3	−0.133	−0.078	−0.069	−0.090	−0.111	−0.022
	0	−0.274	−0.079	−0.119	−0.041 ($p = 0.010$)	−0.064	0.005 ($p = 0.732$)
	1	−0.281	−0.074	−0.131	−0.055	−0.065	−0.036
Mean number of steps	2	−0.277	−0.075	−0.135	−0.078	−0.074	−0.057
	3	−0.259	−0.074	−0.127	−0.103	−0.088	−0.054
	0	0.310	0.064	0.182	0.019 ($p = 0.233$)	0.071	0.290
	1	0.295	0.068	0.181	0.045	0.066	0.252
Entropy of the distribution of reached peaks	2	0.269	0.071	0.165	0.072	0.062	0.208
	3	0.222	0.064	0.132	0.093	0.060	0.144

Table K: Correlation of the greedy adaptive walks outcomes with number of stop codons, conditioned on the number of split codon blocks, for the Ostrov codes. Results for 4 split codon blocks not shown because all codes with 4 split codon blocks have 2 stop codons. All correlations are statistically significant, unless stated otherwise.

	ParD-ParE2	ParD-ParE3	ParB-*parS*	ParB-*NBS*	DHFR
GB1	0.094	0.073	−0.022	−0.006	−0.277
ParD-ParE2		−0.045	0.007	0.021	−0.054
ParD-ParE3			−0.058	−0.013	−0.010
ParB-*parS*				0.059	−0.002
ParB-*NBS*					−0.008

Table L: Correlation of the mean fitness reached in the greedy walks by individual Ostrov codes across the six data sets.

	GB1		ParD-ParE2		ParD-ParE3		ParB-*parS*		ParB-*NBS*		DHFR	
	Mean fitness	P-value	Mean fitness	P-value	Mean fitness	P-value	Mean fitness	P-value	Mean fitness	P-value	Mean fitness	P-value
Pines et al. [2]												
OPT	1.413	0.613	-0.063	0.498	-0.020	0.011	-2.727	0.774	-0.674	0.775	4.354	0.052
OPT-NR	1.207	0.949	-0.032	0.219	-0.045	0.056	-2.583	0.574	-1.652	1.0	4.155	0.177
CMC	1.372	0.722	0.0232	0.108	-0.0397	0.041	-3.160	1.0	-0.142	0.403	4.261	0.096
CMC^2	1.356	0.756	0.0495	0.059	-0.00585	0.004	-1.840	0.276	0.255	0.340	4.485	0.036
REC	1.449	0.503	-0.0143	0.153	-0.102	0.252	-2.496	0.488	-1.449	1.0	4.355	0.052
Ostrov	1.363	0.742	-0.0570	0.428	-0.0145	0.008	-2.674	0.679	-0.804	0.938	4.444	0.044
Calles et al. [5]												
FS20	-1.778	1.0	-2.864	1.0	-2.231	1.0	-7.061	1.0	-8.547	1.0	2.673	1.0
RED20	-0.871	1.0	-2.125	1.0	-0.939	1.0	-7.228	1.0	-8.288	1.0	3.361	1.0
This study												
standard	1.497	0.341	0.061	0.031	-0.125	0.350	-1.492	0.213	0.958	0.155	3.955	0.823
Code A	1.531	0.237	0.021	0.110	-0.057	0.093	-1.228	0.191	1.003	0.149	4.123	0.197
Code B	1.553	0.179	0.032	0.094	-0.066	0.128	-1.320	0.197	0.906	0.164	4.123	0.196
Code C	1.483	0.390	0.062	0.028	-0.046	0.058	-1.458	0.209	0.952	0.156	4.089	0.232
Code D	1.258	0.909	-0.142	0.955	-0.243	0.972	-3.156	1.0	-0.804	0.938	3.928	0.876

Table M: Mean fitness reached in the greedy adaptive walks using the evolvability-promoting codes of Pines et al. [2], evolvability-diminishing codes of Calles et al. [5], and the codes identified in this study (codes A-C promote evolvability, code D diminishes). The p-value equals the proportion of the Ostrov codes that have reached the same or higher fitness in the greedy walks.

1 Artificial inflation of GB1 landscape ruggedness

We reasoned that the weak correlation between code robustness and global peak accessibility might be due to the low variance in landscape ruggedness under the 100,000 amino acid permutation codes. To test this, we artificially inflated the ruggedness of the GB1 landscape under the standard genetic code by separately increasing the number of local peaks and the prevalence of reciprocal sign epistasis.

In particular, to increase the number of local peaks, we chose a number (ranging from 1 to 10,000) of protein sequences at random and set their fitness to a value that ensured that the corresponding region of the genotype network would form a local peak. In particular, we used a value of 2.5, which is halfway between the fitness value of the global peak (WWLA, fitness 2.52) and the second-best binding sequence (FYAA, fitness 2.48). Even when changing the fitness value of the same number of protein sequences, the number of local peaks in the resulting landscapes varies slightly, due to three reasons: (1) the original landscape contains 115 local peaks, some of which might cease to be local peaks if a neighboring sequence is artificially elevated; on the contrary, if a local peak is chosen and its elevation increased, the number of local peaks in the landscape does not change; (2) protein sequences containing the amino acid serine, which is encoded by the split codon block, are encoded by two disconnected regions in the genotype network, and artificially increasing their fitness thus creates two local peaks instead of one; (3) if two chosen sequences are neighbors in the genotype space, the corresponding mRNA sequences form one large plateau, and thus only one local peak is created instead of two.

To artificially increase the prevalence of reciprocal-sign epistasis, we randomly sampled an mRNA sequence of length 12 (i.e., encoding 4 amino acids), making sure it did not translate to the global peak sequence (WWLA) and the translation did not contain any stop codons. We then sampled two mutations in different positions of the sequence that were non-synonymous, both alone and in combination, and such that the single mutants and double mutant did not contain a stop codon. We further required that the single mutants did not translate to the global peak sequence. We then permuted the fitness values of the corresponding protein sequences so that the double mutant had the highest value, the wild type the second highest, and the two single mutants the two lowest values. Since permuting the fitness values of a quadruplet of protein sequences changes the shape of many squares, it is again the case that even among landscapes in which the same number of squares was changed, the proportion of different types of epistasis is variable. We varied the number of squares forced to exhibit reciprocal-sign epistasis from 1 and 100,000.

The resulting landscapes ranged in their number of local peaks from 115 to 3,356 and in their prevalence of reciprocal sign epistasis from 0.047 to 0.130. With these landscapes, we observed a strong correlation between global peak accessibility and the two measures of landscape ruggedness ($R = -0.893$, $p < 2.2 \cdot 10^{-16}$, number of peaks vs. mutational accessibility of the global peak; $R = -0.987$, $p < 2.2 \cdot 10^{-16}$, prevalence of reciprocal-sign epistasis vs. mutational accessibility of the global peak; Fig S) in S1 Text. Moreover, we observe that the moderate effect size is consistent with the range of reciprocal-sign epistasis prevalence in the amino acid permutation codes

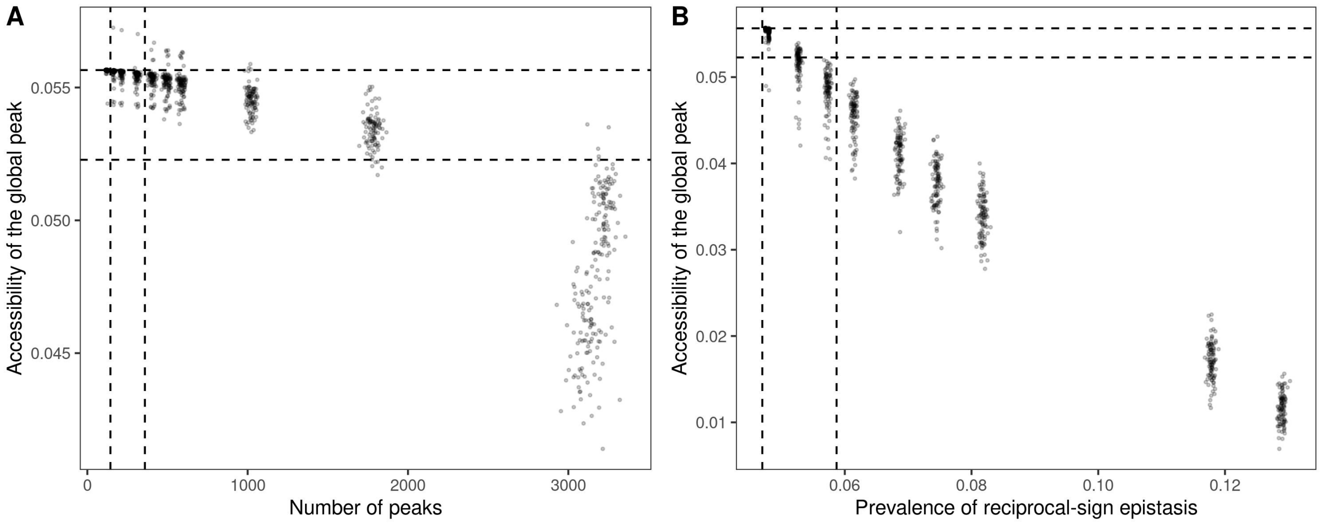

Figure S: Accessibility of the global peak in relation to two artificially-inflated measures of landscape ruggedness, (A) the number of peaks and (B) the prevalence of reciprocal-sign epistasis. The vertical lines show the 0.01 and 0.99 quantiles in the number of peaks and the prevalence of reciprocal sign epistasis, respectively, for the 100,000 amino acid permutation codes. The horizontal lines show the change in accessibility observed among the 100,000 amino acid permutation codes; the distance between them is the difference between the average accessibility of the global peak in landscapes generated using the 1% most and 1% least robust amino acid permutation codes. For visual clarity, the lines are positioned so that the top line coincides with the mutational accessibility of the global peak in the original landscape. Data pertain to the GB1 landscape under the standard genetic code. The data and code required to generate this Figure can be found at https://zenodo.org/records/10677993.

(Fig S in S1 Text, panel B) and that the expected effect size based on the range in the number of peaks would be even lower (Fig S in S1 Text, panel A).

2 Analysis of physicochemical properties from the Aaindex database

In the main text, we defined code robustness based on a discrete categorization of amino acids (Fig C in S1 Text). To see whether our conclusions also hold for other definitions of code robustness, and to discover which particular physicochemical properties of amino acids drive the correlation between code robustness and landscape ruggedness, here we additionally explore definitions of code robustness based on hundreds of different amino acid properties.

In particular, we downloaded the set of 566 different descriptors of amino acids from the AAindex database, version 9.2 [6, 7]. Of those, 13 contained at least one missing value; we discarded those, so the final set contained 553 different amino acid properties. These properties were previously divided into 4 categories: "alpha and turn propensity", "beta propensity", "hydrophobicity", and "other" [8]. For each property p and each of our 100,000 amino acid permutation codes we computed the mean absolute change in the property under genetic code c, $\mathrm{MAC}(p, c)$, as

$$\mathrm{MAC}(p, c) = \frac{1}{|\mathcal{V}|} \sum_{\{v,v'\} \in \mathcal{V}} |p[aa(v, c)] - p[aa(v', c)]|$$

where $\mathcal{V}$ is the set of all codon pairs that are one substitution away from each other (excluding pairs that contain at least one stop codon), $aa(v, c)$ is the amino acid encoded by codon v in genetic code c, and $p[a]$ is the value of property p for amino acid a. $\mathrm{MAC}(p, c)$ quantifies the sensitivity of code c with respect to amino acid property p: if $\mathrm{MAC}(p, c)$ is large, it means that single-nucleotide substitutions tend to cause large changes in property p; if $\mathrm{MAC}(p, c)$ is low, single-nucleotide substitutions tend to preserve property p.

For each property p we then computed Pearson's correlation between the sensitivities of our amino acid permutation codes with respect to property p and the different measures of landscape ruggedness:

$$R(p, \mathbf{T}) = \mathrm{corr}\left(\mathbf{MAC}(p, \mathbf{c}), \mathbf{T}(\mathbf{c})\right),$$

where $\mathbf{MAC}(p, \mathbf{c})$ is the vector of the values of $\mathrm{MAC}(p, c)$ for the set of 100,000 amino acid permutation codes and $\mathbf{T}(\mathbf{c})$ is the vector of values of certain landscape ruggedness measure (e.g., number of peaks) for these codes. For the mutational accessibility of the global peak, we only considered codes that preserve the size of the global peak, relative to the standard genetic code, and under which the global peak consists of a single connected region in the genotype space.

We determined the significance of each $R(p, \mathbf{T})$ by comparison with a null distribution calculated from 1,000,000 randomly generated amino acid 'properties': We generated 1,000,000 null amino acid 'properties' by uniformly sampling 20 random numbers between 0 and 1. For each such null property p_{null}, we computed $\mathbf{MAC}(p_{\mathrm{null}}, \mathbf{c})$ and $R(p_{\mathrm{null}}, \mathbf{T})$ as described above. The significance of the true correlation coefficient is then the proportion of these 1,000,000 null correlation coefficients that are more extreme than the true value. We then corrected the significance values for each landscape ruggedness measure for multiple testing using Benjamini-Hochberg correction [9].

The results are shown in Table N in S1 Text. With the exception of the ParB-*NBS* landscape, we observe many amino acid properties that are consistent with our previous observation that more robust codes cause smoother adaptive landscapes, and only very few that support the opposite statement (i.e., less robust codes implying smoother landscapes). For example, for the GB1 data, 111 out of the 553 properties (20.1% of the tested properties) exhibit a significant negative correlation between code robustness and the prevalence of reciprocal-sign epistasis, whereas there are only 7 properties (i.e., 1.3% of the database) for which the opposite is true. For GB1, the statistically significant properties are enriched in beta-sheet propensity indices and, less consistently across the different landscape ruggedness measures, in alpha-helix propensity and hydrophobicity indices (Table N in S1 Text). The importance of the preservation of hydrophobicity is consistent with three of the four residues (V39, G41, V54) being located in the protein core [10]; however, the consistent significance of beta-sheet propensity indices is somewhat surprising, as only one of the four residues is located in a beta sheet (Fig A in S1 Text). Similarly, the properties that are significant for the DHFR landscape are enriched in hydrophobicity indices, likely as a result of the fact that most peaks have D or E in their second position, which are the two most hydrophobic amino acids. The properties that are significant for the two ParD3 landscapes, as well as the ParB-*parS* landscape, in contrast, are consistently enriched in alpha-helix propensity indices (Table N in S1 Text), which is consistent with both proteins being mostly helical (Fig A in S1 Text).

In sum, the amino acid properties most relevant to code robustness are protein-specific, and depend on the structural and functional properties of the assayed residues in each protein. Increasing code robustness relative to these properties generally results in smoother adaptive landscapes.

	Total (+/- corr.)	Alpha and turn propensity		Beta propensity		Hydrophobicity		Other	
		Observed	Enrichment P-value	Observed	Enrichment P-value	Observed	Enrichment P-value	Observed	Enrichment P-value
**GB1**									
Number of peaks	171 (169/2)	14	1	23	**0.00937**	127	$\mathbf{< 2.2 \cdot 10^{-16}}$	7	1
Magnitude epistasis	45 (5/40)	21	**0.00251**	9	**0.00815**	12	0.973	3	1.00
Simple-sign epistasis	0								
Reciprocal-sign epistasis	118 (111/7)	50	$\mathbf{1.03 \cdot 10^{-4}}$	17	**0.0123**	43	0.762	8	1.00
Accessibility of the global peak	6 (5/1)	0	1	0	1	0	1	6	$\mathbf{3.53 \cdot 10^{-4}}$
**ParD-ParE2**									
Number of peaks	10 (5/5)	3	0.511	0	1	2	0.949	5	0.0980
Magnitude epistasis	62 (11/51)	34	$\mathbf{1.66 \cdot 10^{-6}}$	3	0.880	10	1	15	0.710
Simple-sign epistasis	3 (0/3)	0	1	0	1	1	0.776	2	0.174
Reciprocal-sign epistasis	89 (82/7)	56	$\mathbf{4.09 \cdot 10^{-13}}$	6	0.720	10	1	17	0.961
Accessibility of the global peak	1 (0/1)	1	0.262	0	1	0	1	0	1
**ParD-ParE3**									
Number of peaks	7 (2/5)	0	1	0	1	5	0.0891	2	0.594
Magnitude epistasis	72 (2/70)	56	$\mathbf{< 2.2 \cdot 10^{-16}}$	4	0.834	4	1	8	1
Simple-sign epistasis	48 (44/4)	36	$\mathbf{2.41 \cdot 10^{-12}}$	4	0.537	3	1	5	0.998
Reciprocal-sign epistasis	95 (95/0)	74	$\mathbf{< 2.2 \cdot 10^{-16}}$	5	0.882	4	1	12	1
Accessibility of the global peak	4 (4/0)	1	0.704	1	0.282	2	0.512	0	1
**ParB-*parS***									
Number of peaks	13 (0/13)	0	1	0	1	10	**0.00664**	3	0.713
Magnitude epistasis	32 (2/30)	25	$\mathbf{1.29 \cdot 10^{-9}}$	3	0.474	3	1	1	1
Simple-sign epistasis	38 (34/4)	28	$\mathbf{1.35 \cdot 10^{-9}}$	3	0.592	2	1	5	0.986
Reciprocal-sign epistasis	30 (27/3)	22	$\mathbf{9.48 \cdot 10^{-8}}$	3	0.431	4	1	1	1
Accessibility of the global peak	0								
**ParB-*NBS***									
Number of peaks	13 (0/13)	0	1	0	1	12	$\mathbf{1.11 \cdot 10^{-4}}$	1	0.982
Magnitude epistasis	15 (15/0)	0	1	0	1	14	$\mathbf{1.95 \cdot 10^{-5}}$	1	0.990
Simple-sign epistasis	21 (0/21)	0	1	0	1	20	$\mathbf{9.86 \cdot 10^{-8}}$	1	0.999
Reciprocal-sign epistasis	10 (1/9)	0	1	1	0.564	9	**0.00143**	0	1
Accessibility of the global peak	2 (0/2)	0	1	0	1	2	0.154	0	1
**DHFR**									
Number of peaks	0								
No epistasis	20 (0/20)	0	1	2	0.480	18	$\mathbf{3.65 \cdot 10^{-6}}$	0	1
Magnitude epistasis	10 (0/10)	1	0.952	0	1	9	$\mathbf{1.43 \cdot 10^{-3}}$	0	1
Simple-sign epistasis	5 (5/0)	1	0.781	0	1	4	0.081	0	1
Reciprocal-sign epistasis	39 (39/0)	1	1	10	$\mathbf{7.50 \cdot 10^{-4}}$	27	$\mathbf{1.45 \cdot 10^{-4}}$	1	1
Accessibility of the global peak	14 (0/14)	0	1	1	0.687	13	$\mathbf{4.66 \cdot 10^{-5}}$	0	1

Table N: Amino acid properties that are significantly correlated with landscape ruggedness. For each ruggedness measure, the total number of statistically significant properties is shown (number of positively/negatively correlated properties in parentheses). These properties are then broken down into four categories, for which the number of statistically significant properties is shown, along with the p-value of a one-tailed binomial test for over-abundance of the given category among the significantly correlated properties. Statistically significant p-values (< 0.05, **in bold**) mean that the category is over-represented among the significantly correlated properties, compared to the null expectation. The proportions of the categories in the database are 26.2% alpha and turn propensity, 8.0% beta propensity, 39.2% hydrophobicity, and 26.6% other.
Results for no epistasis only shown for the DHFR landscape, because for the remaining landscapes the proportion of squares exhibiting no epistasis is the same under all amino acid permutation codes (see caption of Table B in S1 Text). For DHFR, the correlations for magnitude, simple-sign, and reciprocal-sign epistasis were computed using the proportions of a given epistasis type among epistatic squares only.

3 Data set-specific definition of code robustness

In the main text, we showed that code robustness, defined as the proportion of 'conservative' substitutions allowed by a code, exhibits a weak inverse correlation with landscape ruggedness. To define 'conservative' substitutions, we categorized amino acids into discrete groups (see Supp. Fig. C), based on the long-standing observation that some amino acid pairs are more exchangeable than others [11, 12, 13]. However, because it was only possible to perform our analyses on a small number of landscapes, which were, in turn, based on only a small number of protein sites, the extent to which this measure of conservativeness applies to each of our data sets is not immediately apparent. For instance, it may be the case that the amino acid exchangeabilities in our individual data sets are markedly different from the general principles of exchangeability deduced from a large number of proteins and protein sites.

To test whether this is the case, we considered a data set-specific definition of code robustness, calculated as the expected change in fitness upon mutation. To do so, we first calculated an empirical amino acid exchangeability matrix for each data set, in which each matrix entry reports the mean absolute change in fitness for the corresponding pair of amino acids across all possible genetic backgrounds (Fig T in S1 Text). For the DHFR landscape, we only considered functional variants. We then defined code robustness as the mean amino acid exchangeability across all possible single-nucleotide substitutions, excluding mutations to or from stop codons.

For each data set, we correlated the empirical amino acid exchangeabilities with those based on physicochemical properties, for all 553 amino acid properties from the Aaindex database, separately [6, 7] (see also Section 2 in S1 Text). The distributions of correlation coefficients are shown in Fig U in S1 Text and the most strongly correlated properties for each data set are listed in Table O in S1 Text. Whereas the empirical amino acid exchangeabilities exhibit strong correlations with physicochemical properties in some cases (e.g., for DHFR, the empirical exhangeabilities exhibit a correlation of $R = 0.983$ with the property FAUJ880112, which lists the negative charge of amino acids), in others they exhibit at best only moderate correlations (e.g., for ParD-ParE2, the strongest correlation is $R = 0.503$, specifically with the property SNEP660101, a measure of alpha-helix propensity). Moreover, for ParB-*NBS*, the empirical exchangeabilities exhibit a negative correlation with the majority of the 553 physicochemical properties (Fig U in S1 Text, panel E). There is therefore often a disconnect between measures of amino acid exchangeability based on the physicochemical properties of amino acids and those based on fitness changes observed in our individual data sets. This helps explain the weak correlations between code robustness and landscape ruggedness reported in the main text, as well as the variation in the strength of this correlation across data sets. Indeed, when we define code robustness based on the empirical exchangeability matrices, using 100,000 codes generated by amino acid permutation restricted to preserve the number of codons per amino acid (see Section 4 in S1 Text), we consistently observe significant inverse correlations with landscape ruggedness for all six data sets (Table P in S1 Text). This observation lends additional support to the hypothesis that robust genetic codes, i.e. codes that preferentially allow conservative mutations, produce smooth fitness landscapes that enhance protein evolvability.

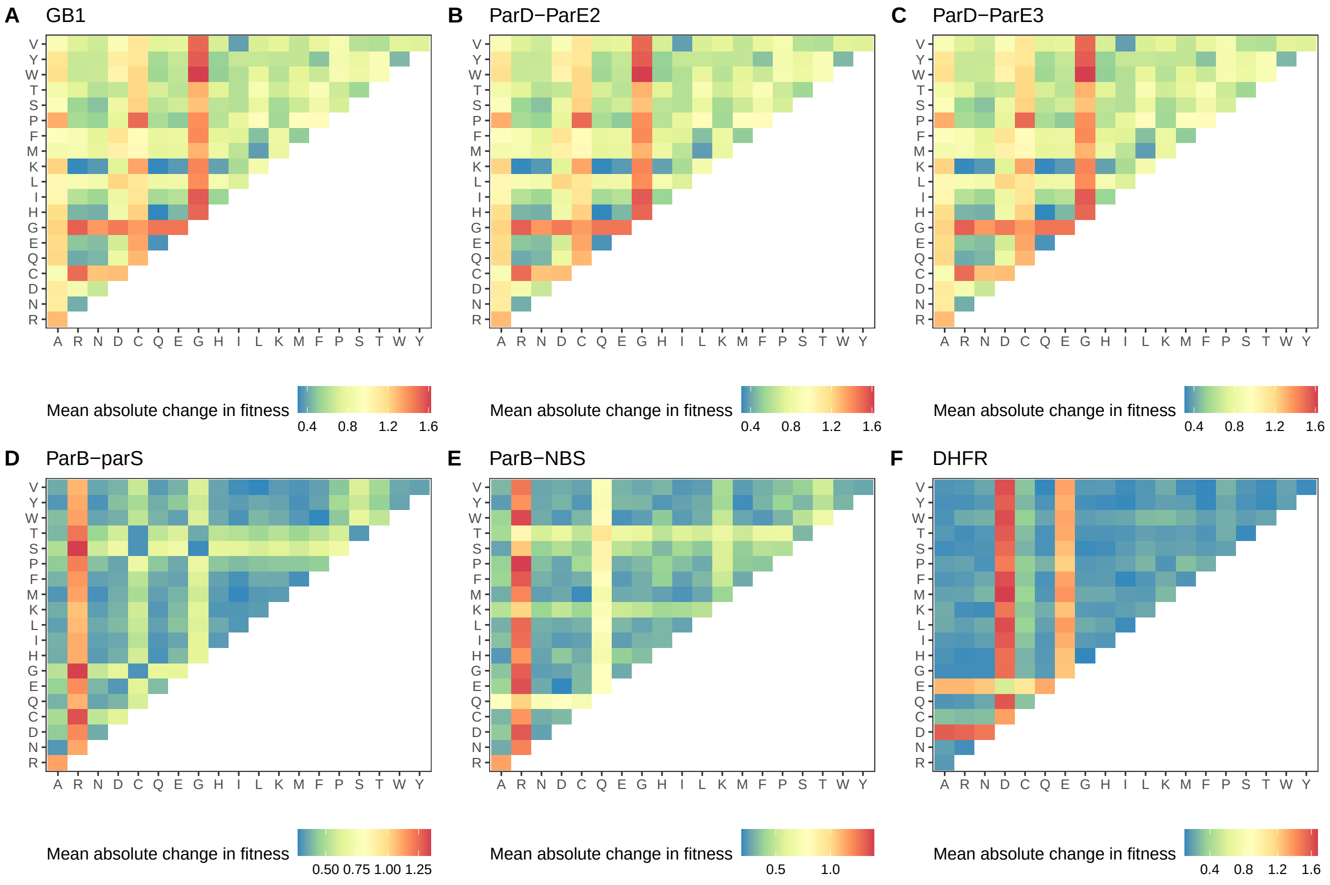

Figure T: Empirical exchangeability matrices for (A) GB1, (B) ParD-ParE2, (C) ParD-ParE3, (D) ParB-parS, (E) ParB-NBS, and (F) DHFR. Each matrix element shows the mean absolute change in fitness across all genetic backgrounds. See color bar. For DHFR, only functional sequences were used for the computation. The data and code required to generate this Figure can be found at https://zenodo.org/records/10677993.

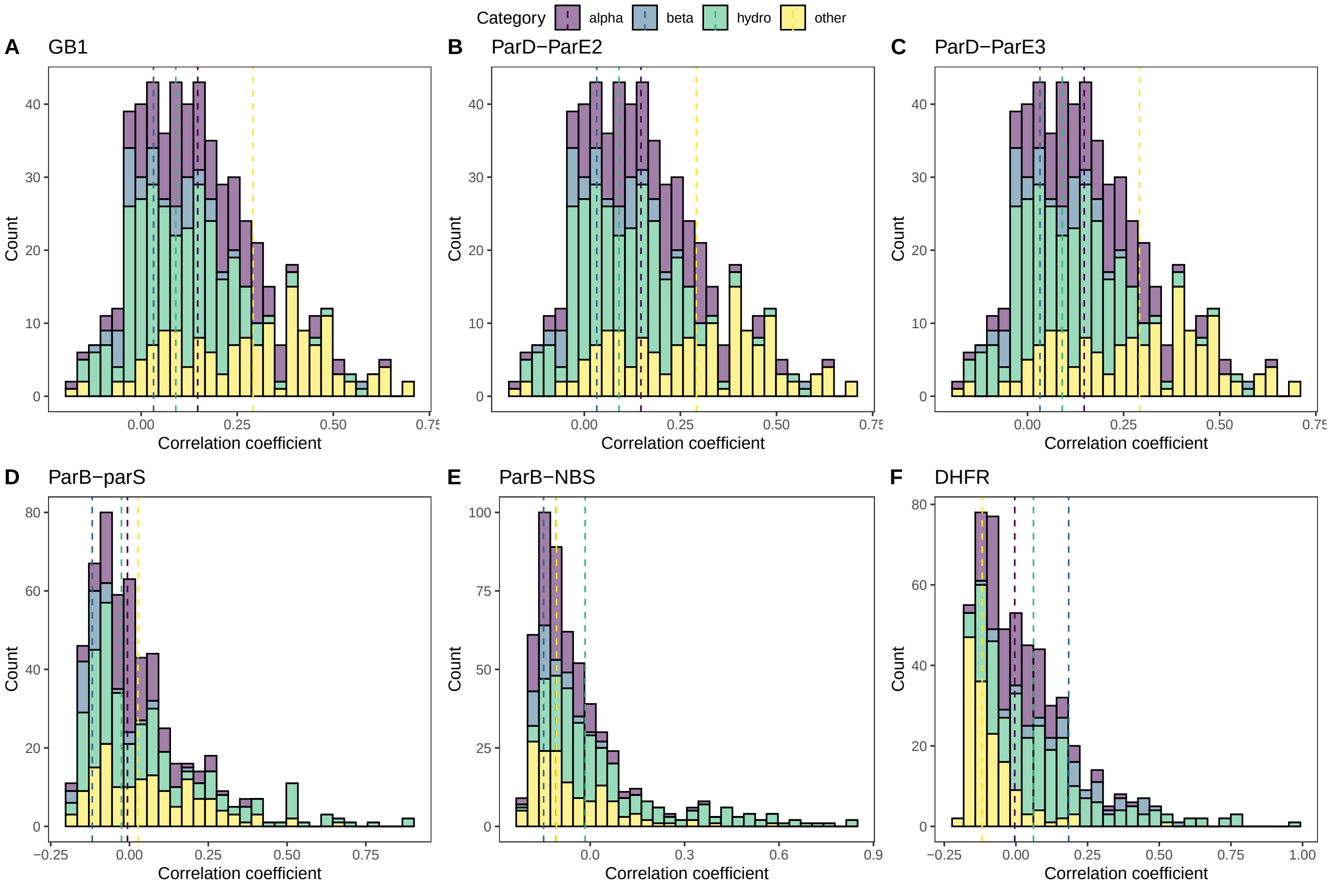

Figure U: Distribution of correlation coefficients for amino acid exchangeabilities measured in terms of absolute fitness change vs. absolute change in physicochemical property, for each of the 553 amino acid properties in the Aaindex database [6, 7]. The properties are colored by their category, according to ref. [8]. The vertical lines denote the median correlation coefficient for each category. The data and code required to generate this Figure can be found at https://zenodo.org/records/10677993.

Property	Description	Category	R	Reference
**GB1**				
KARS160109	Maximum eigenvalue of the weighted Laplacian matrix of the graph	other	0.702	[14]
KARS160108	Average weighted degree (total degree, divided by the number of vertices)	other	0.682	[14]
DAWD720101	Size	other	0.649	[15]
LEVM760104	Side chain torsion angle phi(AAAR)	other	0.646	[16]
KARS160121	Weighted average eigenvalue based on the atomic numbers	other	0.625	[14]
KARS160111	Average eigenvalue of the Laplacian matrix of the the graph	other	0.621	[14]
KOEP990101	Alpha-helix propensity derived from designed sequences	alpha	0.620	[17]
KARS160119	Weighted maximum eigenvalue based on the atomic numbers	other	0.613	[14]
LEVM760103	Side chain angle theta(AAR)	other	0.613	[16]
MAXF760105	Normalized frequency of zeta L	other	0.594	[18]
**ParD-ParE2**				
SNEP660101	Principal component I	alpha	0.503	[19]
YUTK870101	Unfolding Gibbs energy in water, pH7.0	hydro	0.463	[20]
QIAN880135	Weights for coil at the window position of 2	alpha	0.454	[21]
COHE430101	Partial specific volume	other	0.452	[22]
LEVM780106	Normalized frequency of reverse turn, unweighted	alpha	0.451	[23]
TANS770110	Normalized frequency of chain reversal	alpha	0.450	[24]
NAKH900112	Transmembrane regions of mt-proteins	other	0.446	[25]
FAUJ880102	Smoothed upsilon steric parameter	other	0.440	[26]
NAKH920105	AA composition of MEM of single-spanning proteins	other	0.437	[27]
QIAN880133	Weights for coil at the window position of 0	alpha	0.436	[21]
**ParD-ParE3**				
FINA910104	Helix termination parameter at posision j+1	alpha	0.698	[28]
MUNV940105	Free energy in beta-strand region	beta	0.639	[29]
ROBB760104	Information measure for C-terminal helix	alpha	0.630	[30]
QIAN880109	Weights for alpha-helix at the window position of 2	alpha	0.616	[21]
FAUJ880113	pK-a(RCOOH)	alpha	0.606	[26]
MUNV940101	Free energy in alpha-helical conformation	alpha	0.605	[29]
MUNV940104	Free energy in beta-strand region	beta	0.588	[29]
BLAM930101	Alpha helix propensity of position 44 in T4 lysozyme	alpha	0.587	[31]
FINA910102	Helix initiation parameter at posision i,i+1,i+2	alpha	0.586	[28]
QIAN880134	Weights for coil at the window position of 1	alpha	0.580	[21]
**ParB-*parS***				
YUTK870103	Activation Gibbs energy of unfolding, pH7.0	hydro	0.876	[20]
YUTK870104	Activation Gibbs energy of unfolding, pH9.0	hydro	0.875	[20]
EISD860102	Atom-based hydrophobic moment	hydro	0.771	[32]
JACR890101	Weights from the IFH scale	hydro	0.684	[33]
JOND750102	pK (-COOH)	hydro	0.679	[34]
HUTJ700103	Entropy of formation	other	0.652	[35]
YUTK870102	Unfolding Gibbs energy in water, pH9.0	hydro	0.634	[20]
FAUJ880109	Number of hydrogen bond donors	hydro	0.621	[26]
KHAG800101	The Kerr-constant increments	hydro	0.621	[36]
RADA880104	Transfer free energy from chx to oct	hydro	0.564	[37]
**ParB-*NBS***				
YUTK870103	Activation Gibbs energy of unfolding, pH7.0	hydro	0.822	[20]
YUTK870104	Activation Gibbs energy of unfolding, pH9.0	hydro	0.820	[20]
EISD860102	Atom-based hydrophobic moment	hydro	0.755	[32]
FAUJ880109	Number of hydrogen bond donors	hydro	0.727	[26]
JACR890101	Weights from the IFH scale	hydro	0.679	[33]
RADA880107	Energy transfer from out to in(95%buried)	hydro	0.664	[37]
GUYH850105	Apparent partition energies calculated from Chothia index	hydro	0.655	[38]
RADA880104	Transfer free energy from chx to oct	hydro	0.623	[37]
YUTK870102	Unfolding Gibbs energy in water, pH9.0	hydro	0.573	[20]
JOND750102	pK (-COOH)	hydro	0.572	[34]
**DHFR**				
FAUJ880112	Negative charge	other	0.983	[26]
RICJ880105	Relative preference value at N2	hydro	0.790	[39]
RICJ880106	Relative preference value at N3	hydro	0.788	[39]
HOPA770101	Hydration number	hydro	0.781	[40]
WOEC730101	Polar requirement	hydro	0.715	[41]
FINA910101	Helix initiation parameter at posision i-1	hydro	0.710	[28]
CHOP780204	Normalized frequency of N-terminal helix	hydro	0.667	[42]
KLEP840101	Net charge	hydro	0.656	[43]
ROBB760102	Information measure for N-terminal helix	hydro	0.628	[30]
AURR980107	Normalized positional residue frequency at helix termini N2	hydro	0.601	[44]

Table O: Top 10 properties most strongly correlated with the empirical amino acid exchangeabilities, for the six data sets. Property codes and descriptions as in the Aaindex database. Categorization according to [8].

	GB1	ParD-ParE2	ParD-ParE3	ParB-*parS*	ParB-*NBS*	DHFR
Number of peaks	0.416	0.052	0.213	0.049	0.133	0.319
Prevalence of no epistasis	-	-	-	-	-	−0.750
Prevalence of magnitude epistasis	−0.295	−0.217	−0.056	0.248	−0.386	0.586
Prevalence of simple-sign epistasis	−0.267	−0.161	−0.397	−0.485	0.033	−0.676
Prevalence of reciprocal-sign epistasis	0.572	0.567	0.682	0.220	0.557	0.030
Accessibility of the global peak	−0.093	−0.369	−0.017	−0.192	−0.457	−0.486

Table P: Correlation of various measures of landscape ruggedness with an alternative definition of code robustness that is based on the mean fitness change upon mutation, as measured in a specific data set. All correlations are statistically significant. Results for the prevalence of no epistasis are shown only for DHFR, because in the remaining landscapes all amino acid permutation codes have the same proportion of squares with no epistasis. For DHFR, the magnitude, simple-sign, and reciprocal-sign epistasis results correspond to correlation between code robustness and the proportion of given epistasis type among epistatic squares only.

4 Restricted amino acid permutation codes

In the standard genetic code, the number of codons encoding an amino acid ranges from 1 (M, W) to 6 (L, S, R). Because the classical amino acid permutation rewiring scheme does not impose any restrictions on the permutations, the number of codons encoding amino acid X differs amongst rewired codes. This is important from the point of view of code robustness, as code robustness increases if 'special' amino acids, such as G or P, are assigned to small codon blocks. On the other hand, it presents a challenge for the interpretation of our results, because the number of mRNA sequences encoding a given protein variant differs amongst codes, which means different codes yield landscapes with different mean fitness. Moreover, as shown in the main text, the number of mRNA sequences encoding the global peak influences its mutational accessibility and the outcomes of greedy walks.

To check that our results also hold for genetic codes that preserve the number of codons per amino acid, we generated 100,000 genetic codes by amino acid permutation, but restricted the permutations to those that do not change the number of codons encoding each amino acid relative to the standard genetic code. Under these genetic codes, the number of mRNAs encoding each protein variant is exactly the same as in the standard genetic code, and the resulting fitness landscapes thus differ only by which protein variants are reachable from each other and which are not. We then repeated the analyses from the main text using these genetic codes.

We observe qualitatively the same results. In particular, we again observe that more robust codes result in smoother fitness landscapes (Table Q in S1 Text). Consequently, we also see that more robust codes lead to higher fitness reached in simulations of evolution (with the exception of the DHFR landscape) and increased predictability of evolution (Table R in S1 Text).

	GB1	ParD-ParE2	ParD-ParE3	ParB-*parS*	ParB-*NBS*	DHFR
Number of peaks	-0.115	-0.096	-0.057	-0.109	-0.059	-0.003 ($p = 0.342$)
Prevalence of no epistasis	-	-	-	-	-	0.211
Prevalence of magnitude epistasis	0.269	0.055	-0.012	-0.012	0.186	-0.190; -0.102
Prevalence of simple-sign epistasis	-0.090	0.075	0.156	0.171	-0.107	0.115; 0.142
Prevalence of reciprocal-sign epistasis	-0.281	-0.188	-0.214	-0.188	-0.179	-0.127; -0.066
Accessibility of the global peak	0.109	0.171	0.073	0.121	0.017	0.130

Table Q: Correlation of various measures of landscape ruggedness with code robustness, for amino acid permutation codes restricted to preserve number of codons per amino acid. All correlations are statistically significant, unless stated otherwise. Results for the prevalence of no epistasis are shown only for the DHFR landscape, because in the remaining 5 landscapes all amino acid permutation codes have the same proportion of squares with no epistasis. For DHFR, two numbers are shown for the prevalence of magnitude, simple-sign, and reciprocal-sign epistasis; the first one is the correlation between code robustness and the prevalence of a given type of epistasis among all squares, the second is the correlation between code robustness and the prevalence of a given type of epistasis among epistatic squares only. The results for accessibility of the global peak are based on a subset of the genetic codes under which the global peak is formed by single connected region in the sequence space.

	GB1	ParD-ParE2	ParD-ParE3	ParB-*parS*	ParB-*NBS*	DHFR
Mean fitness	0.066	0.229	0.077	$-6.24 \cdot 10^{-4}$ ($p = 0.910$)	0.022	-0.071
Mean number of steps	0.078	0.220	0.082	-0.047	0.095	-0.068
Entropy of the distribution of reached peaks	-0.124	-0.275	-0.122	-0.078	-0.097	-0.004 ($p = 0.239$)

Table R: Correlation of code robustness with the outcomes of greedy adaptive walks for amino acid permutation codes restricted to preserve the number of codons per amino acid as in the standard genetic code. The results are based on a subset of the genetic codes under which the global peak is formed by single connected region in the sequence space. All correlations are statistically significant, unless stated otherwise.

5 Random codon assignment codes

To further verify that our results are insensitive to the choice of rewiring scheme, we also repeated the analyses from the main text for 100,000 genetic codes generated by randomly assigning an amino acid meaning to each of the 61 sense codons, ensuring that each of the 20 amino acids is assigned at least one codon [45, 46, 47]. We refer to these as 'random codon assignment' codes. These differ from the codes generated using amino acid permutation by lacking the synonymous codon block structure of the standard genetic code, making them less realistic as possible alternatives to the standard genetic code than the amino acid permutation codes. As a result of the missing block structure, their average robustness is much lower than that of the amino acid permutation codes ($p < 2.2 \cdot 10^{-16}$, Welch two sample t-test; Fig V in S1 Text).

Consistent with our previous observations, we find that increasing code robustness decreases landscape ruggedness under this alternative rewiring scheme (Table S in S1 Text) and that code robustness is positively correlated with length of greedy adaptive walks and predictability of evolution (Table T in S1 Text).

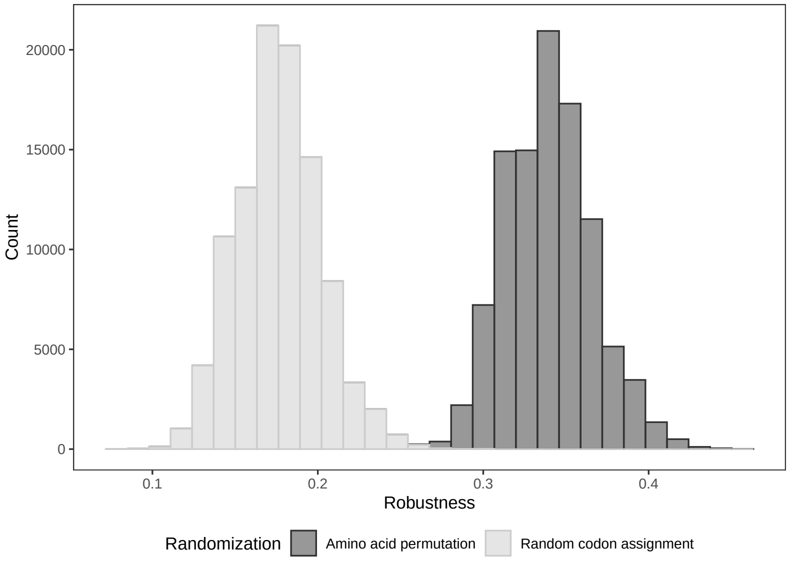

Figure V: Robustness distributions for genetic codes rewired by amino acid permutation and random codon assignment. Data pertain to 100,000 codes per rewiring scheme. The data and code required to generate this Figure can be found at https://zenodo.org/records/10677993.

	GB1	ParD-ParE2	ParD-ParE3	ParB-*parS*	ParB-*NBS*	DHFR
Number of peaks	−0.387	−0.392	−0.329	−0.296	−0.319	−0.283
Prevalence of no epistasis	0.371	0.371	0.371	0.371	0.371	0.189
Prevalence of magnitude epistasis	−0.160	0.133	−0.038	−0.273	−0.127	−0.185
Prevalence of simple-sign epistasis	−0.417	−0.393	−0.249	−0.371	−0.362	−0.182
Prevalence of reciprocal-sign epistasis	−0.476	−0.466	−0.401	−0.406	−0.447	−0.248
Accessibility of the global peak	−0.002, $p = 0.535$	−0.053	−0.228	0.123	0.022	0.047
Accessibility of the global peak (codes preserving the size of the global peak)	0.149	0.134	0.024, $p = 0.190$	0.018, $p = 0.149$	0.065	0.150

Table S: Correlation of various measures of landscape ruggedness with code robustness for random codon assignment codes. All correlations are statistically significant, unless stated otherwise. Unlike for the amino acid permutation codes, the random codon assignment codes differ in the proportion of mutations that are synonymous, and hence also differ in the prevalence of squares showing no epistasis. However, the observed correlation with code robustness is exactly the same for all data sets except DHFR because the prevalence of squares showing no epistasis is determined by the proportion of synonymous mutations in the genetic code. (This is not true for DHFR because of the non-functional variants.)

	GB1	ParE2	ParE3	ParB-*parS*	ParB-*NBS*	DHFR
Mean fitness	0.140; 0.141	0.127; 0.183	−0.0734; 0.092	−0.021; −0.065	0.0869; 0.138	−0.130; −0.157
Mean number of steps	0.418; 0.454	0.259; 0.170	0.266; 0.124	0.164; 0.158	0.204; 0.210	0.114; 0.091
Entropy of the distribution of reached peaks	−0.071; −0.109	−0.072; −0.213	0.0635; −0.123	−0.084; −0.070	−0.0928; −0.118	−0.131; −0.129

Table T: Correlation of code robustness with the outcomes of greedy adaptive walks for the random codon assignment codes. In each cell, the first number gives the correlation in the full set of 100,000 random codon assignment codes, the second number the correlation for the subset of genetic codes that preserve the size of the global peak. (For these codes, the subset of codes that preserve the size of the global peak is not required to also fulfill the condition that the global peak forms a single connected region in the genotype space, because due to the nature of the random codon assignment codes this is almost never the case.) The size of the subset is $n = 4,584$ (GB1), $n = 16,032$ (ParD-ParE2), $n = 6,781$ (ParD-ParE3), $n = 6,484$ (ParB-*parS*), $n = 3,099$ (ParB-*NBS*), $n = 3,059$ (DHFR). All correlations are statistically significant.

6 Landscape dimensionality

One of the most fundamental characteristics of a fitness landscape is its dimensionality, which in our empirical landscapes is determined by the number of variable protein sites, L. Recent theoretical work has shown that landscapes of low dimensionality tend to be more rugged than their higher-dimensional counterparts [48]. We therefore wanted to test the sensitivity of our results, which all pertain to small L, to changes in landscape dimensionality. However, because the number of protein variants grows exponentially with L, it is currently experimentally intractable to obtain combinatorially-complete fitness landscapes of higher dimensionality.

We therefore studied the sensitivity of our results to landscape dimensionality by generating landscapes of even lower dimensionality. We did so by subsetting the GB1 data, which has the most variable sites of any of our data sets ($L = 4$), to contain only $L = 2$ or 3 variable sites. For $L = 3$, there are 80 such landscapes (4 ways to choose 3 sites out of 4×20 different backgrounds) and we generated all of them; for $L = 2$, there are 2,400 such landscapes (6 ways to choose 2 sites $\times 20^2$ different backgrounds) and we randomly sampled 100 of them. We then repeated our analyses from the main text on these low-dimensionality landscapes. The results are summarized in Figs W and X in S1 Text, which depict histograms of correlation coefficients between code robustness and our various measures of protein evolvability. While the variance in the distribution of correlation coefficients is high, code robustness is on average negatively correlated with landscape ruggedness and positively correlated with the mean fitness reached by greedy adaptive walks. Moreover, the strength of these trends tends to increase with L. Taken together, these results suggests our main conclusions are qualitatively insensitive to landscape dimensionality.

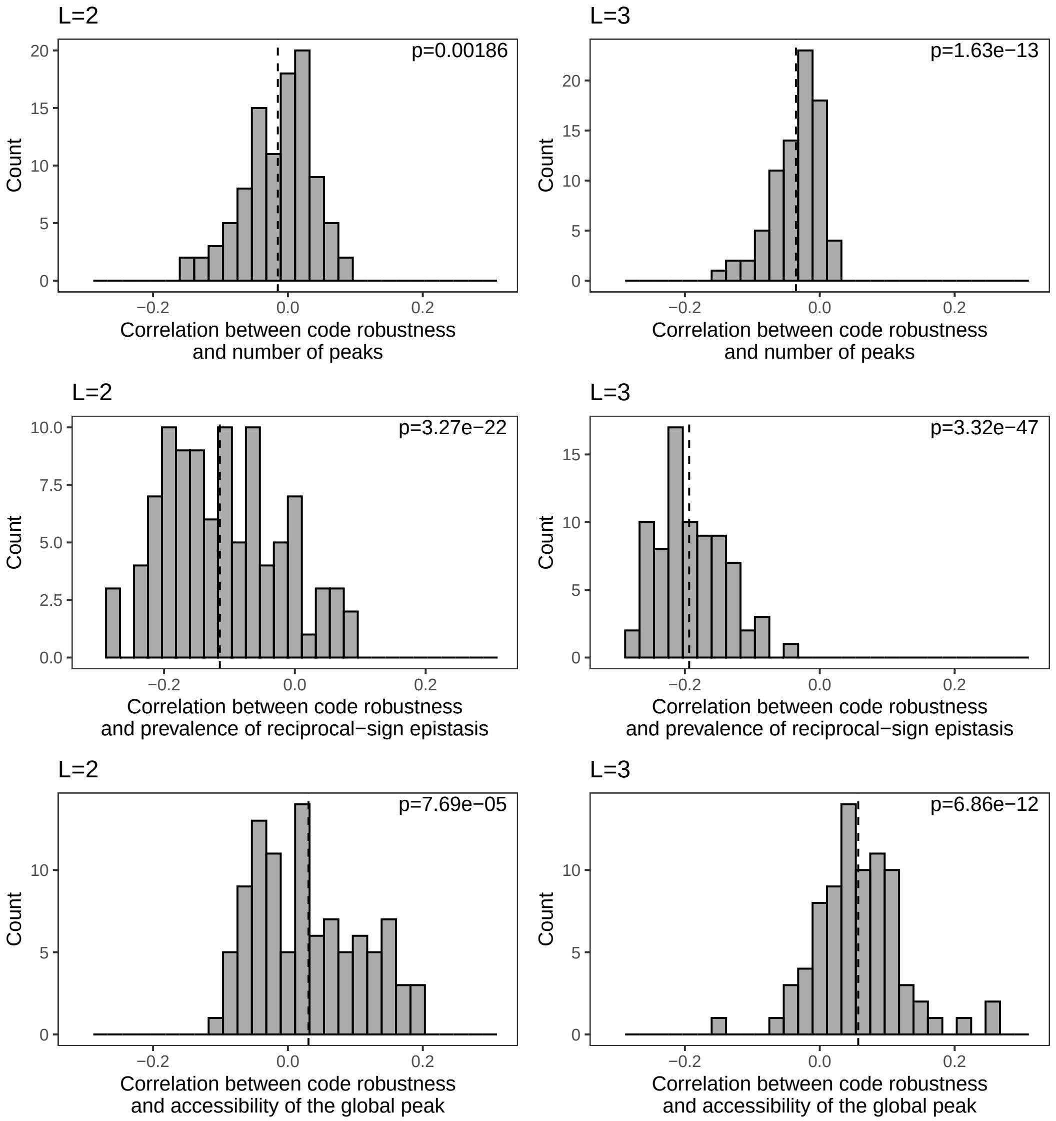

Figure W: Distribution of Pearson's correlation coefficients between different measures of landscape ruggedness and code robustness, for 100 fitness landscapes with $L = 2$ (left column) and 80 fitness landscapes with $L = 3$ (right column). The rows, from top to bottom, correspond to the number of peaks in the landscape, the prevalence of reciprocal-sign epistasis, and the accessibility of the global peak. In each histogram, the dashed line denotes the mean of the distribution. P-value in the upper right corner of each plot corresponds to a one-sample one-sided t-test of the mean being significantly different from 0 (significantly lower than 0 for number of peaks and proportion of reciprocal-sign epistasis, significantly greater than 0 for accessibility of the global peak). Plots are centered on zero to emphasize the weight of each distribution. The data and code required to generate this Figure can be found at https://zenodo.org/records/10677993.

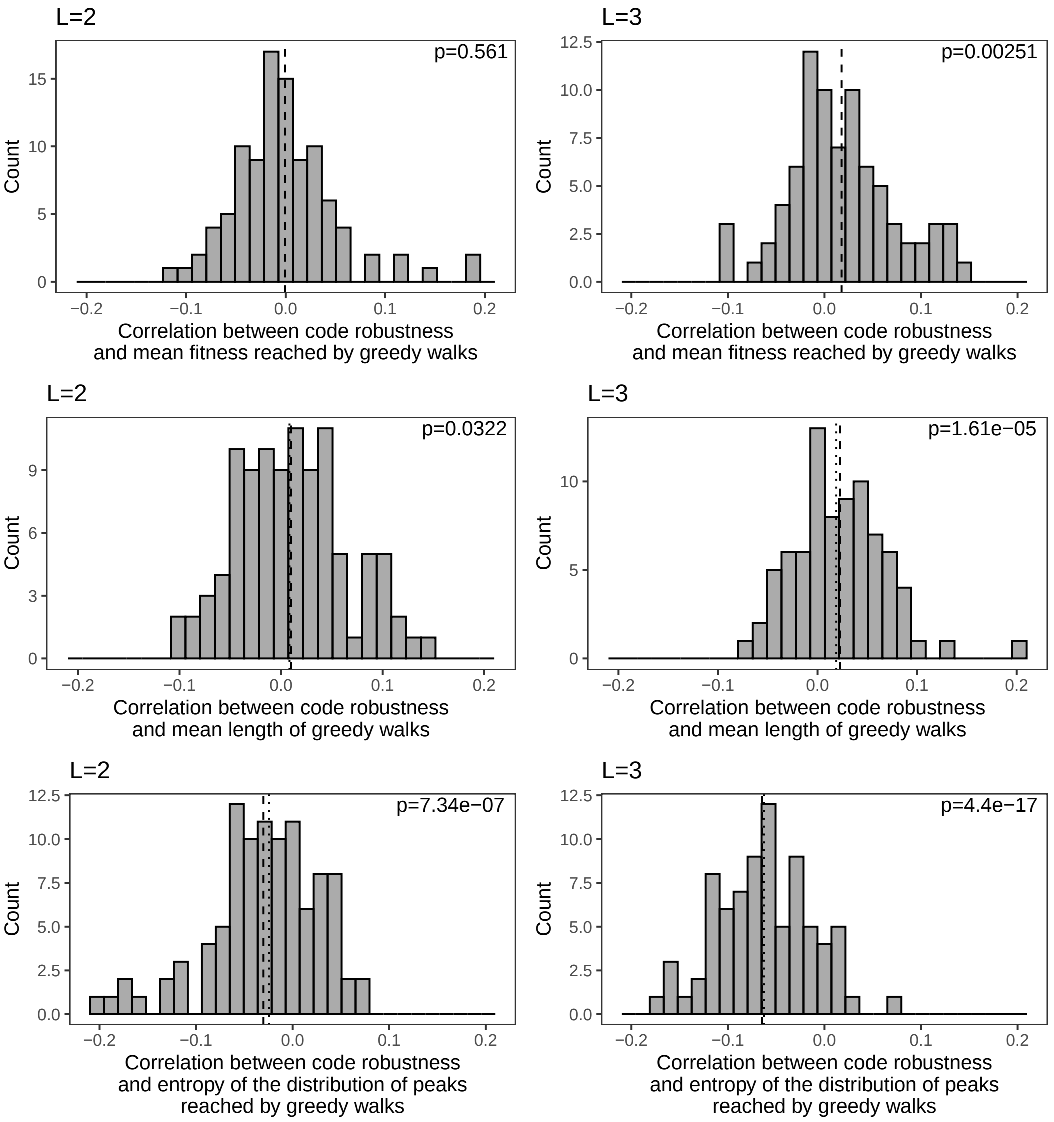

Figure X: Distribution of Pearson's correlation coefficients between the outcomes of greedy adaptive walks and code robustness, for 100 fitness landscapes with $L = 2$ (left column) and 80 fitness landscapes with $L = 3$ (right column). The rows, from top to bottom, correspond to mean fitness, mean number of steps, and entropy of the distribution of reached peaks. In each histogram, the dashed line denotes the mean of the distribution. P-value in the upper right corner of each plot corresponds to a one-sample one-sided t-test of the mean being significantly different from 0 (significantly lower than 0 for entropy of the distribution of reached peaks, significantly greater than 0 for mean fitness and mean number of steps). Plots are centered on zero to emphasize the weight of each distribution. The data and code required to generate this Figure can be found at https://zenodo.org/records/10677993.

7 Causes of the correlation between code robustness and mean fitness reached by greedy adaptive walks

In our simulations of greedy adaptive walks, we observed a positive correlation between code robustness and mean fitness reached by the greedy adaptive walks in 4 out of 6 data sets (GB1, ParD-ParE2, ParD-ParE3, and ParB-*NBS*) and a negative one in the remaining two (ParB-*parS* and DHFR). What is the reason behind these conflicting results? To answer this question we investigated the subsets of landscapes that preserve the size of the global peak and in which the global peak consists of a single connected region in genotype space. One possible explanation is that the landscapes differ in the relationship between code robustness and the mean height of fitness peaks. However, we observe that in all six data sets there is a negative correlation between code robustness and the mean height of fitness peaks ($R = -0.106$, $p = 7.20 \cdot 10^{-11}$, GB1; $R = -0.144$, $p < 2.2 \cdot 10^{-16}$, ParD-ParE2; $R = -0.0794$, $p = 5.81 \cdot 10^{-11}$, ParD-ParE3; $R = -0.168$, $p = 5.25 \cdot 10^{-16}$, ParB-*parS*; $R = -0.00318$, $p = 0.856$, ParB-*NBS*; $R = -0.075$, $p = 5.55 \cdot 10^{-10}$, DHFR).

The mean fitness reached by greedy adaptive walks depends not only on the heights of a landscape's fitness peaks, but also on the sizes of their basins of attraction, i.e., the number of greedy walks that terminate on them. Given the mostly positive relationship between code robustness and mean fitness reached by the greedy walks, we hypothesized that under robust codes the basins of attraction of the high-fitness peaks are relatively larger compared to those of less robust codes; in other words, that under robust codes the adaptive walks tend to converge on a smaller number of high-fitness peaks. Indeed, we observe that with increasing code robustness the Shannon entropy of the distribution of peaks reached by the greedy walks decreases in all six data sets (Table D in S1 Text; Methods). To model the relationship between code robustness, peak height, and basin of attraction more explicitly, we fitted a linear model that, for each of the genetic codes and each data set, predicts the logarithm of the size of the basin of attraction of a peak as a linear function of its height:

$$\log(\text{size of basin}) = \beta_0 + \beta_1(\text{peak height}).$$

The β_1 coefficient controls how fast the size of the basin changes with peak height; for example, using the standard genetic code and the GB1 landscape, the coefficient is 0.591, meaning that if the peak height increases by 1, the size of the basin is expected to increase $\exp(0.591) \approx 1.8$-times. The bigger the β_1 coefficient, the faster the basin of attraction grows with peak height and the more concentrated the ends of the adaptive walks are on the high peaks. Having computed the β_1 coefficients for all genetic codes in the subset of genetic codes that preserve the size of the global peak, we then correlated them with the corresponding robustness. We observe the expected positive correlation in all data sets except for DHFR ($R = 0.141$, $p < 2.2 \cdot 10^{-16}$, GB1; $R = 0.250$, $p < 2.2 \cdot 10^{-16}$, ParD-ParE2; $R = 0.228$, $p < 2.2 \cdot 10^{-16}$, ParD-ParE3; $R = 0.0987$, $p = 2.13 \cdot 10^{-16}$, ParB-*parS*; $R = 0.117$,

$p = 2.40 \cdot 10^{-11}$, ParB-*NBS*; but $R = -0.078$, $p = 1.10 \cdot 10^{-10}$, DHFR). The DHFR result is explained in more detail below. For the remaining five data sets, these analyses show that under robust genetic codes, evolutionary trajectories to adaptation mostly become more predictable, in that they converge on a smaller number of adaptive peaks, and moreover, they preferentially converge on high-fitness peaks.

In summary, the relationship between code robustness and the mean fitness reached by greedy adaptive walks is influenced by two crucial factors: the mean height of peaks in the landscape and the sizes of their basins of attraction. While under robust genetic codes the peaks are on average lower, the high-fitness peaks have larger basins of attraction. This interplay between the average height of peaks and the sizes of their basins of attraction determines the sign of the correlation between code robustness and mean fitness reached by the greedy walks, with our analyses suggesting that in most cases, the larger basins of attraction of high-fitness peaks compensates for their reduced height.

7.1 Greedy walks in the DHFR landscape

To understand the negative correlation between code robustness and mean fitness in the DHFR landscape, we further investigated the greedy adaptive walks under 100,000 amino acid permutation codes that preserve the number of codons per amino acid as in the standard genetic code (see also Section 4 in S1 Text). We used this set of genetic codes to eliminate the potentially confounding factor of different number of codons per amino acid among the classical amino acid permutation codes. Also under these codes, we observe a negative correlation between code robustness and mean fitness (Table R in S1 Text).

We observed that vast majority (99.3%) of the greedy walks terminates on local peaks with C, D, or E in their second position, with the XCX peaks having a significantly lower fitness than the XDX and XEX peaks (mean fitness -0.861 vs. 3.334, $p < 2.2 \cdot 10^{-16}$, Welch two sample t-test). Consequently, mean fitness reached by the greedy walks is strongly negatively correlated with the proportion of walks that terminate on an XCX peak ($R = -0.958$, $p < 2.2 \cdot 10^{-16}$). We thus hypothesized that the observed correlation between code robustness and mean fitness might be due to increased probability of reaching the XCX peaks under robust codes. Indeed, we see a positive correlation between code robustness and the proportion of greedy walks terminating at XCX peaks ($R = 0.060$, $p < 2.2 \cdot 10^{-16}$). To find out why, we divided the 100,000 amino acid permutation codes into two groups – those where the proportion of greedy walks terminating at XCX peaks is lower than 5% and those where the proportion is larger (Fig Y in S1 Text) – and tried to identify whether these genetic codes differ systematically in any way. We observe that all the codes in the low XCX proportion group allow a C-D or C-E mutation (or both), while none of the codes in the high XCX proportion group do. The proportion of greedy walks that reach an XCX peak under a given genetic code is thus largely determined by whether the genetic code allows a C-D or C-E mutation. This is because under codes that allow a C-D or C-E mutation, the intermediate-fitness XCX sequences neighbor the high-fitness XDX/XEX sequences, and are thus less likely to be local peaks (0.572 vs. 4.98 local XCX peaks in

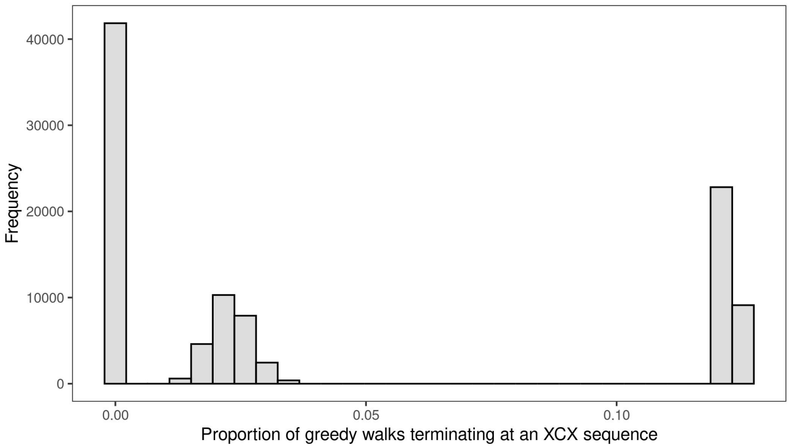

Figure Y: Histogram of the proportion of greedy walks terminating at an XCX sequence, in the set of 100,000 amino acid permutation codes restricted to preserve number of codons per amino acid as in the standard genetic code. The data and code required to generate this Figure can be found at https://zenodo.org/records/10677993.

the codes that do allow a C-D or C-E mutation and in codes that do not, resp., $p < 2.2 \cdot 10^{-16}$, Welch two sample t-test) and, if they are local peaks, their fitness is on average higher (0.963 vs. -0.305, $p < 2.2 \cdot 10^{-16}$, Welch two sample t-test) and their basin of attraction smaller (250.89 vs. 396.53 greedy walks, $p < 2.2 \cdot 10^{-16}$, Welch two sample t-test) than under codes that do not allow either of these two mutations. At the same time, we observe that robust codes are less likely to allow the C-D and C-E mutations (code robustness 0.342 vs. 0.345 for codes that do allow a C-D or C-E mutation and for codes that do not allow either of these mutations, resp., $p < 2.2 \cdot 10^{-16}$, Welch two sample t-test), because both C-D and C-E mutations are considered non-conservative (Fig C in S1 Text).

To summarize, the observed negative correlation between code robustness and mean fitness observed in the DHFR landscape can be explained by a particular idiosyncracy in the landscape, in which the mean fitness is largely determined by whether the genetic code allows a C-D or C-E mutation. The negative correlation arises because these mutations are less likely to be allowed under robust codes.

8 Weak mutation adaptive walks

In the main text, we modeled evolution using greedy adaptive walks, which correspond to a strong-mutation-strong-selection regime. To understand how our results depend on population genetic conditions, we also performed weak mutation adaptive walks. The weak mutation adaptive walks represent adaptive evolution under the regime where mutations occur so infrequently that any mutation will either go to extinction or to fixation prior to the arrival of a subsequent mutation. The probability of fixation depends on both the improvement in fitness and the population size, which controls the strength of genetic drift. For each genetic code, each landscape and each choice of one of four different population sizes, we simulated 100,000 random walks, initialized in randomly chosen mRNA sequences. In each subsequent step, a random single-nucleotide mutation was proposed, and accepted with probability

$$P_{\text{accept}} = \begin{cases} \frac{1-\exp(f_{\text{old}}-f_{\text{new}})}{1-\exp(N(f_{\text{old}}-f_{\text{new}}))} & \text{if } f_{\text{old}} \neq f_{\text{new}} \\ \frac{1}{N} & \text{otherwise} \end{cases}$$

where f_{old} denotes the fitness of the current genotype, f_{new} the fitness of the proposed genotype, and N is the population size. This corresponds to the exact fixation probability under the Moran process [49]. We ran each adaptive walk for 500 steps, using population sizes $N \in \{10; 100; 10,000; 1,000,000\}$.

We obtained qualitatively the same results as for the greedy adaptive walks for the set of 100,000 amino acid permutation codes (Table U in S1 Text), as well as for the Ostrov codes (Tables V, W, and X in S1 Text).

	GB1	ParD-ParE2	ParD-ParE3	ParB-*parS*	ParB-*NBS*	DHFR
$N = 10$	0.120	0.124	0.050	−0.058, $p = 0.0057$	0.0891	−0.123
$N = 100$	0.096	0.155	0.029, $p = 0.018$	−0.081	0.121	−0.123
$N = 10,000$	0.091	0.148	0.018, $p = 0.128$	−0.083	0.119	−0.129
$N = 1,000,000$	0.091	0.148	0.018, $p = 0.131$	−0.083	0.119	−0.128

Table U: Correlation of code robustness with the mean fitness reached after 500 steps of the weak mutation adaptive walks, for different population sizes N, using the amino acid permutation codes. Correlations are statistically significant unless specified otherwise. Data pertain to the subset of amino acid permutation codes that preserve the size of the global peak and under which the global peak forms a single connected region in the genotype space.

	GB1	ParD-ParE2	ParD-ParE3	ParB-*parS*	ParB-*NBS*	DHFR
$N = 10$	0.300	0.295	−0.141	0.170	0.111	−0.186
$N = 100$	0.268	0.380	−0.050	0.148	−0.207	−0.194
$N = 10,000$	0.306	0.410	0.009, $p = 0.0056$	0.159	−0.238	−0.174
$N = 1,000,000$	0.307	0.410	0.010, $p = 0.0014$	0.160	−0.239	−0.173

Table V: Correlation of code robustness with the mean fitness reached after 500 steps of weak mutation adaptive walks, for different population sizes N, using the Ostrov codes. All correlations are statistically significant unless stated otherwise. All results pertain to the subset of codes that preserve the size of the global peak and under which the global peak forms a single connected region in the genotype space.

	GB1	ParD-ParE2	ParD-ParE3	ParB-*parS*	ParB-*NBS*	DHFR
$N = 10$	-0.117	-0.200	-0.087	-0.064	-0.262	0.221
$N = 100$	-0.014, $p = 0.294$	-0.160	0.049	0.0025, $p = 0.763$	-0.056	0.224
$N = 10,000$	0.104	-0.006, $p = 0.428$	0.185	0.058	0.013, $p = 0.170$	0.227
$N = 1,000,000$	0.105	-0.003, $p = 0.703$	0.187	0.058	0.014, $p = 0.144$	0.227

Table W: Correlation of the number of split codon blocks with the mean fitness reached after 500 steps of weak mutation adaptive walks, for different population sizes N, using the Ostrov codes. All correlations are statistically significant unless specified otherwise. All results pertain to the subset of codes that preserve the size of the global peak and under which the global peak forms a single connected region in the genotype space.

	Number of split codon blocks	GB1	ParD-ParE2	ParD-ParE3	ParB-*parS*	ParB-*NBS*	DHFR
	0	-0.255	-0.088, $p = 0.00268$	-0.044, $p = 0.037$	-0.031, $p = 0.450$	-0.107, $p = 0.011$	-0.677
	1	-0.145	-0.072	-0.008, $p = 0.337$	-0.013, $p = 0.464$	-0.157	-0.291
$N = 10$	2	-0.084	-0.050	0.011, $p = 0.040$	0.004, $p = 0.752$	-0.159	-0.184
	3	-	-	0.016, $p = 0.0017$	-	-	-0.110
	0	-0.376	-0.229	-0.077, $p = 2.42 \cdot 10^{-4}$	-0.029, $p = 0.480$	-0.048, $p = 0.253$	-0.575
	1	-0.246	-0.145	-0.033	0.0056, $p = 0.745$	-0.079	-0.246
$N = 100$	2	-0.149	-0.077	0.0011, $p = 0.828$	0.025, $p = 0.043$	-0.082	-0.163
	3	-	-	0.021	-	-	-0.102
	0	-0.769	-0.765	-0.565	-0.080, $p = 0.051$	-0.108, $p = 0.010$	-0.760
	1	-0.631	-0.626	-0.495	-0.023, $p = 0.179$	-0.104	-0.385
$N = 10,000$	2	-0.458	-0.432	-0.402	0.013, $p = 0.281$	-0.086	-0.263
	3	-	-	-0.275	-	-	-0.170
	0	-0.773	-0.634	-0.573	-0.081, $p = 0.048$	-0.109	-0.749
	1	-0.635	-0.771	-0.504	-0.024, $p = 0.169$	-0.104	-0.381
$N = 1,000,000$	2	-0.463	-0.440	-0.411	0.013, $p = 0.290$	-0.086	-0.261
	3	-	-	-0.282	-	-	-0.169

Table X: Correlation of the number of stop codons, conditioned on the number of split codon blocks, with the mean fitness reached after 500 steps of weak mutation adaptive walks, using the Ostrov codes. Results for 4 split codon blocks not shown because all codes with 4 split codon blocks have 2 stop codons. All correlations are statistically significant, unless specified otherwise. All results pertain to the subset of codes that preserve the size of the global peak and under which the global peak forms a single connected region in the genotype space.

9 Fitness landscape visualizations

In this section, we provide a more detailed description of the fitness landscape visualizations presented in Fig 4 for protein GB1 under the standard genetic code, two robust codes, and two non-robust codes.

9.1 Standard genetic code

Fig 4A shows our visualization of the GB1 landscape under the standard genetic code. Looking down Diffusion Axis 1 (Fig 4A, vertical axis), we see that Region 1 (characterized by 41G) is at the top of this axis whereas Region 3 (characterized by 41F or L and 54G or T) is at the bottom, indicating that under long-term purifying selection for GB1 functionality it would take an extremely long time for a population to evolve from Region 1 (which contains the wild-type sequence) to Region 3. The reason is that under the standard genetic code, neither 41F nor 41L is accessible from 41G, and so high-fitness paths from Region 1 to Region 3 instead pass through Region 2, which remains accessible from both Regions 1 and 3.

Besides reducing the connectivity between regions of high-fitness sequences that are accessible to each other in amino acid sequence space, different genetic codes can also restrict the connectivity within these high-fitness regions, or can even break such a region into several disconnected pieces. Under the standard genetic code, we see both of these phenomena, as Region 1 is spread along Diffusion Axis 2, with 41G-54L at one end and the connection to Region 3, 41G-54A, at the other, and Region 3 is in fact broken into two pieces (defined by 54G and 54T, which are not accessible to each other under the standard genetic code) and spread along Diffusion Axis 3. These two pieces of Region 3 are then connected by a set of high-fitness sequences comprising 41L or 41F together with 45A, which are adjacent to both pieces of Region 3 (since under the standard genetic code G and T are both accessible from A). The end result is that while in amino acid sequence space any two highly fit sequences are typically connected by a high-fitness path with at most 4-5 substitutions, in nucleotide space a typical trajectory from Region 1 to Region 3 contains many more substitutions, many of which must be accumulated in a specific order (Fig I in S1 Text).

Nonetheless, we see that the standard genetic code manages to retain evolvability in several different ways. First, we see the genotype network remains connected, such that high-fitness protein variants in distant reaches of sequence space are mutationally-accessible from one another via a series of intermediates that are also of high fitness. This connectedness is important for evolvability, because an evolving population can diffuse across the genotype network to produce new phenotypic variants, and populations with sufficiently high mutation rates will accumulate genetic diversity, which can be revealed as phenotypic variation upon environmental change [50]. Second, whereas traversing from one end of the network to another typically requires many mutations, this is not always the case. For example, in Fig 4A bottom right, we can see that distant pieces of the genotype network are in fact accessible to each other via S_4, the larger of the two disconnected sets of codons for S. We call such a path a "wormhole", as it allows a population to jump from one region of the genotype network to another. Finally, an important

aspect of evolvability is modularity, which in this case refers to amino acids positions that can evolve relatively independently from each other and which produces extended regions of amino acid sequence space where mutations can be accumulated in any order. Under any given genetic code, such regions can either remain connected or be broken into separated pieces, with the maintenance of connectivity resulting in a grid-like region of the visualization. We see such a region at the bottom of Diffusion Axis 1, where F and L at position 41 can be combined with any of G, A and T at position 54.

9.2 Robust genetic codes

Under Robust Code A, Region 1 is disconnected from the remaining high-fitness protein variants in Regions 2 and 3, as can be seen when the landscape is visualized along Diffusion Axis 1 (Fig 4B). The reason is that this code does not allow substitutions from G to any of the key intermediate amino acids that connect Region 1 to Regions 2 or 3 (C, L, F, A; panel A in Fig G in S1 Text). In contrast, Regions 2 and 3 are connected, and form a large 2-dimensional grid-like structure that spreads out along Diffusion Axes 2 and 3. The grid is formed by variants at position 54 along Diffusion Axis 2 and by variants at position 41 along Diffusion Axis 3. However, note that the grid is imperfect, in that it contains some “holes” that correspond to less fit amino acid combinations (not in the top 1% of sequences) at positions 41 and 54, such as 41C-54T or 41S-54V. The grid also contains “bypasses” that connect pairs of protein variants via indirect paths along each axis of the grid. For example, C and M are directly accessible under Robust Code A, however, it is also possible to pass via a F intermediate (see the upper right corner of the grid).

The high-level topology of the genotype network is similar under Robust Code B, except that now it is the 41L-54T portion of Region 3 that is disconnected from the rest of the genotype network. The resulting fitness valley is the largest barrier to diffusion and hence dominates Diffusion axis 1 (Fig H in S1 Text), but the rest of the genotype network is connected and its structure is well-captured by Diffusion Axes 2 and 3 (Fig 4C).

9.3 Non-robust genetic codes

Under Non-Robust Code A (Fig 4D), the genotype network adopts a long linear structure stretching from 41L-54V to 41M-54T. This greatly diminishes evolvability, both because traversing among amino acid sequences that differ even in only one position (e.g., 41L-54V and 41L-54G) may require many nucleotide mutations, and because only very few mutational paths exist between any pair of sequences in the genotype network. This is especially true for 41M sequences. 41M is compatible with amino acids at position 54 typical for Region 2 and 3, i.e., A, G, T, and V, and 41M sequences thus usually cluster with sequences in Regions 2 and 3 (see e.g. Fig 4B). However, under Non-Robust Code A, M is encoded by a single codon (UGG; panel C in Fig G in S1 Text), which differs by more than one mutation from any of the codons for the other high-fitness amino acids at position 41. As a result, only very few mutational paths exist from the 41M sequences to other high-fitness protein variants. Despite

the mostly linear structure of the genotype space, though, this genotype network, similar to the one under the standard genetic code, has a “wormhole”, in which 39W 40W 41L 54P sequences bridge otherwise distant regions of the genotype network, namely the 41L-54V and 41L-54G sequences. Under Non-Robust Code B (Fig 4E), we also observe limited connectivity amongst the high-fitness protein variants, with the 41L-sequences disconnected from the rest along Diffusion Axis 1 and the remaining sequences laid out in a star-like geometry along Diffusion Axis 2 and 3.

10 Epistasis under the Ostrov codes

The Ostrov codes can vary in the number of split codon blocks and the number of stop codons they contain. The effect of increasing the number of split codon blocks on the prevalence of the different forms of epistasis follows intuition: As the number of split codon blocks increases, the number of synonymous mutations decreases, thus decreasing the prevalence of squares with no epistasis. And among the epistatic squares, increasing the number of split codon blocks increases the prevalence of simple and reciprocal sign epistasis (Table H and Fig M in S1 Text). However, the effect of increasing the number of stop codons on the different forms of epistasis is more complicated (Table J and Fig P in S1 Text). First, we observe a strong positive correlation between the number of stop codons and the prevalence of no epistasis. This is because the more stop codons a genetic code has, the more mRNAs contain at least one stop codon, and hence the more squares that consist entirely of sequences containing stop codons. As we have assigned the same fitness value to all sequences containing stop codons (Methods), these squares will be classified as exhibiting no epistasis. Second, contrary to expectation, we observe a strong positive correlation between the number of stop codons and the prevalence of magnitude epistasis, and a strong negative correlation between the number of stop codons and simple, as well as reciprocal, sign epistasis (Table J in S1 Text).

To understand these results, we must think of the types of squares that mRNA sequences containing stop codons can be part of. Not considering the trivial squares consisting only of sequences containing at least one stop codon, there are 6 possible configurations, which we depict in Fig Z in S1 Text. In the following, we will mostly focus on the configurations in Panels A and B, and discuss the remaining 4 configurations at the end. Configuration A involves squares where the “wild type” sequence contains one stop codon and one of the mutations changes the stop codon to a sense codon, while the second mutation happens in any of the remaining codons (Panel A of Fig Z in S1 Text). Configuration B involves squares where the wild type sequence contains one stop codon and both mutations change the stop codon to a sense codon (Panel B of Fig Z in S1 Text). In both cases we assume that the double mutant does not contain any stop codons. To understand the influence these squares have on the prevalence of epistasis, we need to know what types of epistasis squares A and B can exhibit and how many of these squares there are.

All the type A squares show magnitude epistasis, regardless of the fitness values of the two sequences without stop codons. How many squares of this type are there? While the exact number will depend on the particular location of the stop codons in the genetic code, we can estimate the number to be roughly

$$N_A = L \cdot n_{\mathrm{STOP}} \cdot n_{\mathrm{sense}}^{L-1} \cdot n_{\mathrm{STOP}\to\mathrm{sense}} \cdot (L-1) n_{\mathrm{sense}\to\mathrm{sense}},$$

where L is the total number of codons in the sequence, n_{STOP} is the number of stop codons in the code, n_{sense} is the number of sense codons in the code, $n_{\mathrm{STOP}\to\mathrm{sense}}$ is the expected number of mutations from a stop codon to a sense codon, and $n_{\mathrm{sense}\to\mathrm{sense}}$ is the expected number of mutations from a sense codon to another sense codon. The

first three terms quantify the number of sequences containing exactly one stop codon, while the fourth and fifth terms quantify the number of possible mutations that would give rise a type A square.

For the type B squares, the type of epistasis depends on the exact fitness values of the three sequences that do not contain stop codons, which can cause the square to exhibit magnitude, simple sign, or reciprocal sign epistasis. Using reasoning similar to that above, we estimate the number of these squares as roughly

$$N_B = L \cdot n_{\text{STOP}} \cdot n_{\text{sense}}^{L-1} \cdot n_{\text{STOP}\to\text{sense}} \cdot n_{\text{STOP}\to\text{sense}}.$$

Even without taking into account the fact that the two mutations must happen in two different nucleotide positions, and hence the last two terms are at most $9 \cdot 6$, N_A is clearly at least $(L-1)$-times bigger than N_B. In other words, it is much more likely that the second mutation happens in a different codon, than that both mutations happen in the stop codon. As all type A squares exhibit magnitude epistasis, we would thus expect that the prevalence of magnitude epistasis, relative to simple sign and reciprocal sign epistasis, increases as the number of stop codons increases.

Do the remaining possible configurations (Panels C and D) change the result? All squares in Panel C exhibit magnitude epistasis and will thus further increase the prevalence of magnitude epistasis. On the other hand, squares in Panel D, consisting of a wild type and a double mutant that do contain a stop codon and two single mutants that do not, always exhibit reciprocal-sign epistasis. However, in the Ostrov codes the number of such squares is extremely low, due to the fact that the stop codons can be placed in only a small handful of positions; in fact, the maximum number of type D squares in the whole landscape is 2 (see Panel D of Fig Z in S1 Text), so their effect on reciprocal sign epistasis is negligible.

To conclude, contrary to expectation, increasing the number of stop codons increases the prevalence of magnitude epistasis, relative to simple sign and reciprocal sign epistasis, because the number of squares with two neighboring sequences containing stop codons (as in Panels A and C of Fig Z in S1 Text) is much larger than the number of squares where a low-fitness sequence containing a stop codon separates two higher-fitness variants without stop codons (Panels B and D).

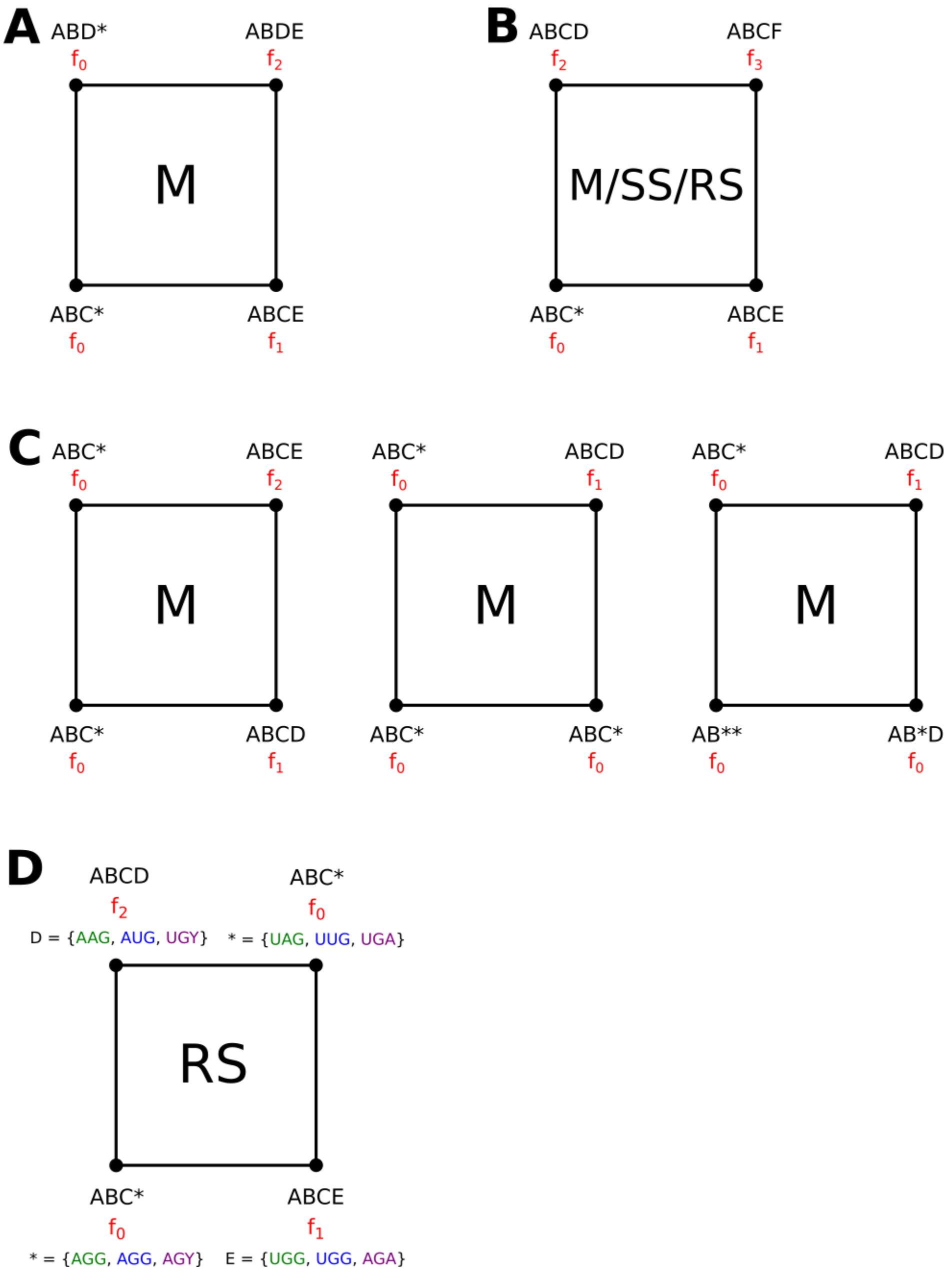

Figure Z: Possible configurations of squares involving at least 1 and at most 3 sequences containing stop codons, for sequences of length $L = 4$, with the stop codon occupying the last position. A, B, C, D, and E denote arbitrary amino acids, not necessarily different from each other. Fitness values are denoted by $f_0, \ldots, f_3$, with $f_0 < f_i$ for $i = 1, 2, 3$, while we assume no particular ordering of f_1, f_2, and f_3. The letters in the middle of the squares denote the possible types of epistasis a given configuration can exhibit; M = magnitude epistasis, SS = simple-sign epistasis, RS = reciprocal-sign epistasis. In D, possible assignments of the last codon, based on the Ostrov codes, are listed in green, blue, and violet; notice that the green and blue assignments are not compatible with the violet one, as AGG and AGA codons need to be assigned the same amino acid (or stop signal), and cannot thus at the same time encode a stop signal (green, blue) and an amino acid (violet). Thus, the maximum number of squares of type D in a landscape caused by an Ostrov code is 2.

11 Examples of Ostrov codes promoting or diminishing evolvability

In the main text we discussed common features of Ostrov codes enhancing or diminishing evolvability. Namely, we observed that genetic codes promoting evolvability tend to have fewer split codon blocks, fewer stop codons, and higher physicochemical robustness, while the opposite is true for genetic codes that diminish evolvability, with the exception of the number of stop codons (Fig 6 and Fig R in S1 Text). Here, we present concrete examples of genetic codes following these design principles.

Panels A and C in Fig AA in S1 Text show the two genetic codes with the highest robustness of the consistently high-ranking Ostrov codes ('Evolvable Ostrov Code A' and 'Evolvable Ostrov Code B'). They have a robustness of 0.385, the minimal number of zero split codon blocks, and the minimal number of two stop codons. Engineering these codes in a living organism is in principle possible with existing technology, although it requires the reassignment of five of the seven freed codons, which is no small feat. In contrast, most experimental studies of rewired genetic codes only change the meaning of the UAG stop codon [51]. Bacterial strains containing no genomic TAG, as well as a variety of orthologous translation systems that decode UAG as a nonstandard amino acid are commercially available, so engineering a strain with reassigned UAG is relatively straightforward. The best of such codes, in terms of mean fitness reached in our simulations, is depicted in Panel C of Fig AA ('Evolvable Ostrov Code C'). It reassigns UAG to glutamate, and it ranks among the best 25% of Ostrov codes for all data sets except for GB1 (Table M in S1 Text).

How do these codes compare to the standard genetic code in our evolutionary simulations? Evolvable Ostrov Code A ranks better in terms of fitness on five out of six data sets, and Evolvable Ostrov Codes B and C rank better than the standard genetic code in four out of six cases (Table M in S1 Text). It is also worth noting that in four out of six data sets, the mean fitness reached under the standard genetic code, as well as Evolvable Ostrov Codes A, B, and C is higher than when using genetic codes specifically designed for increased evolvability [2] (Table M in S1 Text), even though they are much easier to engineer than those proposed by Pines et al. [2]. Interestingly, for the ParB-*NBS* data set, some of the codes proposed by Pines et al. perform worse than any of the 194,481 Ostrov codes. This further confirms that decreasing code robustness does not in general lead to an increase in evolvability.

While for the genetic codes promoting evolvability it is possible to optimize all three design principles at once, this is not possible for the codes that diminish evolvability. For example, a genetic code where all free codon blocks are assigned to a stop signal will have the maximum possible number of stop codons (9), but it will also have the minimal number of split codon blocks (0). It is thus impossible to highlight one genetic code that would be expected to decrease evolvability the most based on our design principles. Instead, in Panel D of Fig AA we show a genetic code that ranks among the bottom 12.5% of codes for all six data sets (Table M in S1 Text). We again compared the level to which this code diminishes evolvability with codes specifically designed to slow down the rate of evolution [5] (Table M in S1 Text). While reducing evolvability beyond the majority of the Ostrov codes, the mean fitness reached in adaptive walks using genetic code D is still much higher than when using the codes proposed by Calles

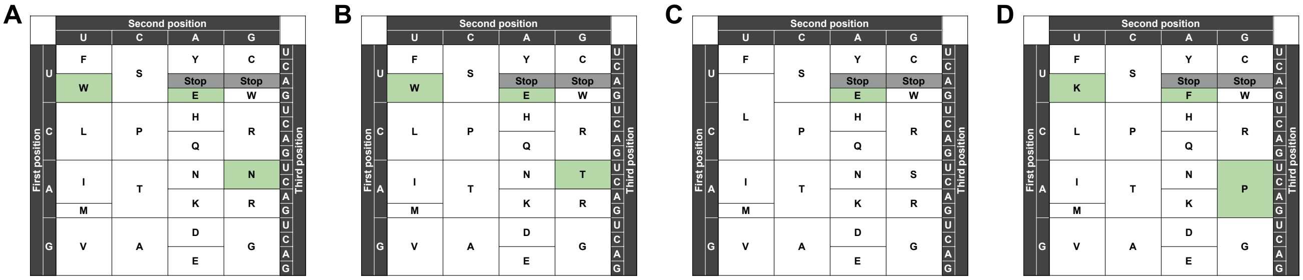

Figure AA: Examples of codes promoting (A-C) or diminishing (D) evolvability, identified based on their robustness, number of stop codons, and number of split codon blocks, as well as the results of the greedy adaptive walks. Changes compared to the standard genetic code are highlighted in green.

et al. [5]. However, we emphasize that, similar to the codes proposed by Pines et al. [2], the codes proposed by Calles et al. require extensive genome recoding, such that the majority of codons are 'null', meaning they encode neither an amino acid nor a stop signal. We hope the design principles we have identified here will provide guidance for engineering genetic codes that significantly enhance or diminish evolvability, but remain within reach of current technology.

References

[1] Zhou J, Wong MS, Chen WC, Krainer AR, Kinney JB, McCandlish DM. Higher-order epistasis and phenotypic prediction. Proceedings of the National Academy of Sciences. 2022;119(39):e2204233119.

[2] Pines G, Winkler JD, Pines A, Gill RT. Refactoring the Genetic Code for Increased Evolvability. mBio. 2017;8(6).

[3] McCandlish DM. Visualizing fitness landscapes. Evolution. 2011;65(6):1544–1558.

[4] Ostrov N, Landon M, Guell M, Kuznetsov G, Teramoto J, Cervantes N, et al. Design, synthesis, and testing toward a 57-codon genome. Science. 2016;353(6301):819–822.

[5] Calles J, Justice I, Brinkley D, Garcia A, Endy D. Fail-safe genetic codes designed to intrinsically contain engineered organisms. Nucleic Acids Research. 2019;47(19):10439–10451.

[6] Kawashima S, Ogata H, Kanehisa M. AAindex: Amino Acid Index Database. Nucleic Acids Research. 1999;27(1):368–369.

[7] Kawashima S, Kanehisa M. AAindex: Amino Acid index database. Nucleic Acids Research. 2000;28(1):374.

[8] Bartonek L, Braun D, Zagrovic B. Frameshifting preserves key physicochemical properties of proteins. Proceedings of the National Academy of Sciences. 2020;117(11):5907–5912.

[9] Benjamini Y, Hochberg Y. Controlling the False Discovery Rate: A Practical and Powerful Approach to Multiple Testing. Journal of the Royal Statistical Society Series B (Methodological). 1995;57(1):289–300.

[10] Nisthal A, Wang CY, Ary ML, Mayo SL. Protein stability engineering insights revealed by domain-wide comprehensive mutagenesis. Proceedings of the National Academy of Sciences. 2019;116(33):16367–16377.

[11] Dayhoff M, Schwartz R, Orcutt B. A model of evolutionary change in proteins. In: Dayhoff M, editor. Atlas of Protein Sequence and Structure. vol. 5. Washington, D. C.: National Biomedical Research Foundation; 1978. p. 345–352.

[12] Henikoff S, Henikoff JG. Amino acid substitution matrices from protein blocks. Proceedings of the National Academy of Sciences. 1992;89(22):10915–10919.

[13] Yampolsky LY, Stoltzfus A. The Exchangeability of Amino Acids in Proteins. Genetics. 2005;170(4):1459–1472.

[14] Kakraba S, Knisley D. A graph-theoretic model of single point mutations in the cystic fibrosis transmembrane conductance regulator. Journal of Advances in Biotechnology. 2016;6(1):780–786.

[15] Dawson DM. In: Brock DJH, Mayo O, editors. The biochemical genetics of man. New York: Academic Press; 1972. p. 1–38.

[16] Levitt M. A simplified representation of protein conformations for rapid simulation of protein folding. Journal of Molecular Biology. 1976;104(1):59–107.

[17] Koehl P, Levitt M. Structure-based conformational preferences of amino acids. Proceedings of the National Academy of Sciences. 1999;96(22):12524–12529.

[18] Maxfield FR, Scheraga HA. Status of empirical methods for the prediction of protein backbone topography. Biochemistry. 1976;15(23):5138–5153.

[19] Sneath PHA. Relations between chemical structure and biological activity in peptides. Journal of Theoretical Biology. 1966;12(2):157–195.

[20] Yutani K, Ogasahara K, Tsujita T, Sugino Y. Dependence of conformational stability on hydrophobicity of the amino acid residue in a series of variant proteins substituted at a unique position of tryptophan synthase alpha subunit. Proceedings of the National Academy of Sciences. 1987;84(13):4441–4444.

[21] Qian N, Sejnowski TJ. Predicting the secondary structure of globular proteins using neural network models. Journal of Molecular Biology. 1988;202(4):865–884.

[22] Cohn EJ, Edsall JT. Protein, Amino Acid, and Peptides. New York: Reinhold; 1943.

[23] Levitt M. Conformational preferences of amino acids in globular proteins. Biochemistry. 1978;17(20):4277–4285.

[24] Tanaka S, Scheraga HA. Statistical Mechanical Treatment of Protein Conformation. 5. Multistate Model for Specific-Sequence Copolymers of Amino Acids. Macromolecules. 1977;10(1):9–20.

[25] Nakashima H, Nishikawa K, Ooi T. Distinct character in hydrophobicity of amino acid compositions of mitochondrial proteins. Proteins: Structure, Function, and Bioinformatics. 1990;8(2):173–178.

[26] Fauchère JL, Charton M, Kier LB, Verloop A, Pliska V. Amino acid side chain parameters for correlation studies in biology and pharmacology. International Journal of Peptide and Protein Research. 1988;32(4):269–278.

[27] Nakashima H, Nishikawa K. The amino acid composition is different between the cytoplasmic and extracellular sides in membrane proteins. FEBS Letters. 1992;303(2-3):141–146.

[28] Finkelstein AV, Badretdinov AY, Ptitsyn OB. Physical reasons for secondary structure stability: α-Helices in short peptides. Proteins: Structure, Function, and Bioinformatics. 1991;10(4):287–299.

[29] Muñoz V, Serrano L. Intrinsic secondary structure propensities of the amino acids, using statistical ϕ–ψ matrices: Comparison with experimental scales. Proteins: Structure, Function, and Bioinformatics. 1994;20(4):301–311.

[30] Robson B, Suzuki E. Conformational properties of amino acid residues in globular proteins. Journal of Molecular Biology. 1976;107(3):327–356.

[31] Blaber M, jun Zhang X, Matthews BW. Structural Basis of Amino Acid α Helix Propensity. Science. 1993;260(5114):1637–1640.

[32] Eisenberg D, McLachlan AD. Solvation energy in protein folding and binding. Nature. 1986;319(6050):199–203.

[33] Jacobs RE, White SH. The nature of the hydrophobic binding of small peptides at the bilayer interface: implications for the insertion of transbilayer helices. Biochemistry. 1989;28(8):3421–3437.

[34] Jones DD. Amino acid properties and side-chain orientation in proteins: A cross correlation approach. Journal of Theoretical Biology. 1975;50(1):167–183.

[35] Hutchens JO. Heat capacities, absolute entropies, and entropies of formation of amino acids and related compounds. In: Sober HA, editor. Handbook of Biochemistry. Cleveland, Ohio: Chemical Rubber Co.; 1970. p. B60–B61.

[36] Khanarian G, Moore WJ. The Kerr effect of amino acids in water. Australian Journal of Chemistry. 1980;33:1727–1741.

[37] Radzicka A, Wolfenden R. Comparing the polarities of the amino acids: side-chain distribution coefficients between the vapor phase, cyclohexane, 1-octanol, and neutral aqueous solution. Biochemistry. 1988;27(5):1664–1670.

[38] Guy HR. Amino acid side-chain partition energies and distribution of residues in soluble proteins. Biophysical Journal. 1985;47(1):61–70.

[39] Richardson JS, Richardson DC. Amino Acid Preferences for Specific Locations at the Ends of α Helices. Science. 1988;240(4859):1648–1652.

[40] Hopfinger AJ. Intermolecular interactions and biomolecular organizations. New York: Wiley; 1977.

[41] Woese CR. Evolution of the genetic code. Naturwissenschaften. 1973;60(10):447–459.

[42] Chou PY, Fasman GD. In: Prediction of the Secondary Structure of Proteins from their Amino Acid Sequence. John Wiley & Sons, Ltd; 1979. p. 45–148.

[43] Klein P, Kanehisa M, DeLisi C. Prediction of protein function from sequence properties: Discriminant analysis of a data base. Biochimica et Biophysica Acta (BBA) - Protein Structure and Molecular Enzymology. 1984;787(3):221–226.

[44] Aurora R, Rosee GD. Helix capping. Protein Science. 1998;7(1):21–38.

[45] Caporaso JG, Yarus M, Knight RD. Error Minimization and Coding Triplet/Binding Site Associations Are Independent Features of the Canonical Genetic Code. Journal of Molecular Evolution. 2005;61:597–607.

[46] Rozhoňová H, Payne JL. Little Evidence the Standard Genetic Code Is Optimized for Resource Conservation. Molecular Biology and Evolution. 2021;38(11):5127–5133.

[47] Tripathi S, Deem MW. The Standard Genetic Code Facilitates Exploration of the Space of Functional Nucleotide Sequences. Journal of Molecular Evolution. 2018;86:325–339.

[48] Greenbury SF, Louis AA, Ahnert SE. The structure of genotype-phenotype maps makes fitness landscapes navigable. Nature Ecology & Evolution. 2022;6(11):1742–1752. doi:10.1038/s41559-022-01867-z.

[49] Moran PAP. Random processes in genetics. Mathematical Proceedings of the Cambridge Philosophical Society. 1958;54(1):60–71.

[50] Zheng J, Payne JL, Wagner A. Cryptic genetic variation accelerates evolution by opening access to diverse adaptive peaks. Science. 2019;365(6451):347–353.

[51] Mukai T, Lajoie MJ, Englert M, Söll D. Rewriting the Genetic Code. Annual Review of Microbiology. 2017;71(1):557–577.
