# Supplementary material for: Robust genetic codes enhance protein evolvability
Source: PLoS Biol. 2024 May 16;22(5):e3002594. doi: 10.1371/journal.pbio.3002594 (PMC11098591; doi:10.1371/journal.pbio.3002594)
Supplement: Attachment — Submitted filename: responses.pdf [file pbio.3002594.s002.pdf]

Dear Dr Payne,

Thank you for your patience while your manuscript "Protein evolvability under rewired genetic codes" was peer-reviewed at PLOS Biology. It has now been evaluated by the PLOS Biology editors, an Academic Editor with relevant expertise, and by four independent reviewers.

You'll see that the reviewers are broadly positive (though reviewer #3 is somewhat sceptical regarding the magnitude of advance), but three of them raise a number of concerns that must be addressed before we can consider it further. In light of the reviews, which you will find at the end of this email, we would like to invite you to revise the work to thoroughly address the reviewers' reports.

IMPORTANT: We discussed the reviewers' comments with the Academic Editor, who sent the following additional guidance that you might find helpful when deciding how to proceed with your revisions:

(1) Shorten the manuscript. I agree with reviewer 1 that it is far too long, and kind of meanders around, especially in the second half of the results. But I also think that it is very well written and clearly explained, and it would be a disservice to ask the authors to reduce the explanatory text. Hence, I like the idea of removing a part of the results altogether, or perhaps moving some things to the SI and only briefly mentioning it in the main text. E.g. the network analysis adds relatively little value but takes up a lot of space, as do sections 2.6 and 2.7.

We have followed the editor's and reviewers' suggestions and moved the results deemed non-essential to the supplementary material, shortening the main text by roughly 25%. We have opted to keep the network visualizations in the main text, because we feel they provide a compelling demonstration of how the genetic code influences the structure of fitness landscapes, but have greatly reduced the length of this section as well as the length of former sections 2.6 and 2.7.

(2) Address the impact of the method used to calculate robustness and clarify some of the methods that are missing or unclear (as suggested by reviewer 2). Which physicochemical properties were used, and which ones were most important? Also, on line 158 there's a mention of imputation of fitness landscapes and missing sequence variants, but there are no details about this in the methods. I am not sure exactly what was missing, how it was imputed, and whether this impacts the fitness landscape and/or the outcomes.

Per Reviewer 2's suggestion, we have repeated our analyses using a protein-specific definition of code robustness, with qualitatively the same results as before. This analysis is presented in Supplementary Note S3. We believe that, together with the analysis of 553 different amino acid properties (now presented in Supplementary Note S2), this allays any concerns regarding our definition of code robustness.

We have clarified the methods as suggested by Reviewer 2, as well as the imputation by empirical variance component regression. We wish to stress that the imputation of missing variants only involves a handful of sequence variants in only one of the data sets (6.6% of the GB1 data set); most of the fitness values were directly measured in the input data and we show in Supp. Fig. S2 that the fitness values inferred by empirical variance component regression are strongly correlated with the raw data.

(3) Clearly discuss the very weak correlations and different outcomes for the different proteins, and resulting biological implications, as indicated by reviewer 3. I think these issues do reduce the novelty and impact of the results; at the very least they should be clearly discussed. The low dimensionality of the dataset (reviewer 4) is similarly an important concern that should be discussed.

We have made changes throughout the text to clarify our message regarding the strengths of the correlations, the different outcomes for the different proteins, and the non-exceptionality of the standard genetic code for some proteins.

We have added the dimensionality analysis suggested by Reviewer 4 to show that our results are qualitatively insensitive to the dimensionality of the data (Supplementary Note S6). This analysis also shows that quantitatively, the strengths of the correlations between code robustness and our measures of protein evolvability tend to increase as dimensionality increases. We therefore speculate that analyses of landscapes of even higher dimensionality, when they become available, will only reinforce our results.

Reviewer #1:
[identifies himself as Stephen Freeland]

Protein evolvability under rewired genetic codes
Hana Rozhoňová, Carlos Martí-Gómez, David M. McCandlish and Joshua L. Payne

Before delving into the intellectual content of this manuscript, let me state how well written it is - well organized and with a clarity of written English that surpasses most manuscripts I review. As a reviewer, this quality of preparation makes a real difference to how easily I can assess the content. As a potential reader, this contributes directly to how likely I am to actually read the work, and to cite it in my own future works. Thank you to the authors, and well done, for this care in preparation!

Thank you for the kind words.

This paper tackles the important and fascinating question of how molecular evolution would vary were amino acid "meanings" distributed to genetic code words (codons) differently than the pattern found within the standard genetic code. The standard genetic code had become established as the single, dominant pattern by which genes are translated into proteins by the time of LUCA. Molecular evolution for more or less all life on Earth has proceeded under the influence of this pattern for 3.5 billion years. The influence of the pattern is evident within all statistical summaries of accepted mutations, since the inception of the PAM matrix, and yet a wealth of evidence indicates that the pattern was itself an outcome of evolution, not a biophysical constraint. Many different branches of biological science, from bioinformatics and synthetic biology to evolutionary theory and "origins", are therefore directly interested in the data presented by the question addressed by this manuscript. This important question is currently underdeveloped within the literature and the authors explain their significance well. Citations are thorough and provide an excellent guide for all readers to understand what has been done on all fronts (including the growing technological reality of engineered, artificial genetic codes). Indeed the general level and thoroughness of citations throughout this manuscript is one of its many strengths. Perhaps the one citation that I was surprised not to see in the introduction is to the deeply similar (though strictly analogous) question/investigation made by Stadlers, Fontana and Wagner for RNA: (J Theor Biol, (2001) Nov 21;213(2):241-74. doi: 10.1006/jtbi.2001.2423.
"The topology of the possible: formal spaces underlying patterns of evolutionary change")

Thank you for the suggestion. We have added the citation to the third paragraph of the introduction (line 31).

Having established that the manuscript is well written, the question is of broad interest and that the significance of the work is well developed, the next observation I would offer is that the approach (methods) seem very sensible - impressively thoughtful in navigating the opportunities and limitations of current scientific capabilities, both computational and informational (i.e. the data that exists to serve an investigation of this question). I particularly like the use of the saturated sequence "maps" for two real proteins that are each involved in measurable binding as a proxy for fitness. I understand why it is only a few sites that can be considered and this is state of the art for all practical purposes. I do think this is a limitation worth pointing out more heavily in the discussion, if only to motivate future work on more powerful computing and/or richer phenotypic maps of the future.

Following the reviewer's suggestion, and in response to comments from Reviewers 2 and 4, we have added some additional comments to the Discussion that address this limitation and suggest future work on larger landscapes (lines 610-615).

Turning to results, my opinion is that the statistical approaches used to analyze results are equally well thought out - in particular, they are elegantly simple to derive insights appropriate to the limitations and assumptions of the study. Maybe that is a fancy way of saying I felt confident that I understood what was being measured, I could see its direct relevance to the conclusions being drawn and I was persuaded by the data. Well done! For what it is worth, for the exact reason stated in my previous sentence, the single metric that I found most compelling was the number of adaptive peaks - I am a little cynical about reading too much into more sophisticated metrics of smoothness: here, they are well presented; in other literature exploring evolvability, I have seen authors place too much faith in a highly sophisticated metric without remembering how dependent its meaning is upon their underlying assumptions. The

current work skirts closest to this danger in Figure 5, but the thoughtful text in section 2.7 (and even lines 745-746) rescues readers from reading too much into premature over extrapolation of ideals for "the perfect code"!

Conclusions/Discussion
Sensible, well developed and do not extend beyond findings - in fact, and excellent job here to guide readers new to this thinking about what exactly has and has not been discovered here.

Summary: A great paper, of wide interest - well done!

Thank you for the positive assessment of our work!

Other comments:
My only suggestion for substantive is very tentative, and really one for the editor and authors to discuss. This is a long paper, and I believe it could potentially work better as two back-to-back papers: Parts 1 and 2. The most obvious dividing line for me would seem to be "part 1: measuring the influence of the code on fitness landscapes" and "Part 2: simulating effect of the code on evolutionary pathways"

To be clear, there is nothing wrong with the paper as one big whole - My worry is that it might not get the readership it deserves (# readers, and depth of careful reading) if presented as one monolithic test. It is, to me, a "magnum opus" for this group's work over a long and careful period of development. I would humbly offer the opinion that both PLoS and the authors might benefit from finding a dividing line and breaking it into 2 chapters for the audience.

We agree that the paper was originally too long. In response to this comment, as well as the suggestion made by the academic editor, we moved the non-essential parts of the results to the supplements, shortening the main text by roughly 25%.

Reviewer #2:

The paper by Rozhonova et al. addresses an interesting and important question: to what extent does robustness of the genetic code facilitate or hinder the production of adaptive variation. While this question has received prior treatment, it remains unanswered for a number of technical reasons. The current work addresses several of these issues by using data from combinatorically complete deep mutational scans to characterize protein function and explore how the shape of these empirically based landscapes change as the genetic code is computationally 'rewired'. Overall the work is well written, with sufficient detail to understand what was done, why it was done, and the conclusion that the authors' draw from the results. In general, I have very little to add that would likely improve the paper and I think it is a strong addition to the field. However, there are a few caveats to the work worth pointing out.

We thank you for the positive assessment of our work.

1. My largest concern with the work is the definition of robustness used and how this may influence the results. The definition of robustness relies on the long standing observation that some amino acids have more similar biochemical properties than others. Current, and previous work by others, uses this observation to code sets of amino acids as being equivalent and that mutations between those amino acids are robust, while changes between amino acids in different sets are not robust. I understand the use of this simplification, but I'm concerned about how it could influence the papers conclusions. In particular, it is well established biochemical similarities between some amino acids lead to greater exchangeabilities during evolution, but that the ability to exchange two amino acids at any particular site may be idiosyncratic. For example, I and V are routinely seen to flip back and forth in alignments and very often have similar effects on protein function in deep mutational scanning data sets. However, they aren't always exchangeable and this property depends on the particular protein and site examined. My concern is that robustness is measured based on global patterns, while evolvability is dependent on the specific data set analyzed and that this mismatch, and in particular the way in which the amino acids are divided into sets, may influence the conclusions.

This concern is amplified by the authors' analysis of alternative ways to group sets of amino acids by hundreds of other properties. While very few of these sets resulted in the opposite conclusion that robustness generates more rugged landscapes, the majority resulted in no relationship between robustness and ruggedness. This is also not the exact same analysis as conducted earlier in the paper - it connects the different ways of sorting amino acids into groups to ruggedness, but not to evolvability itself. Given the weak correlations overall, how different ways of grouping the amino acids to establish robustness would affect evolvability isn't clear. Furthermore, the analysis as conducted was on the entire protein and not site specific, which again is the necessary scale on which the idiosyncrasies in amino acid exchangeability are observed.

One idea that could alleviate this concern would be to use the data itself to determine which amino acids are functionally similar for each protein at each particular site and then reanalyze the different genetic codes under a protein and site specific estimate of robustness. It may be that this ends up being very similar to one of the already measured groupings, or it may be distinct in some particular way. Regardless, knowing the relationship between robustness and evolvability under such a model would remove most of my concerns about the definition of robustness because robustness would be estimated from the specific data itself instead of generic tendencies of amino acid equivalence. There may also be other ways to test this that would be sufficient.

We thank the reviewer for this insightful comment. We agree that amino acid exchangeabilities can be idiosyncratic in landscapes that were built only from a couple of sites, as the ones we use here. We have followed the reviewer's suggestion to compute a measure of code robustness that is specific to each data set. We are happy to report that our results are qualitatively insensitive to this alternative definition of code robustness, and that quantitatively, they are even stronger when using this alternative definition. This analysis is reported in Supplementary Note S3 and discussed on lines 276-284.

2. My only other major concern is that figure 4 and the corresponding analyses are quite hard to follow. In part, this is because the analysis method is very abstract, incorporating numerous aspects of evolution on a particular network. I appreciate the authors' attempts to provide an intuitive explanation, but what a 'diffusion axis' is still remains unknown

to me. More problematically, what is meant by a 'barrier to diffusion' is abstract and not a standard metric in the field. Additional issues are that the axis units don't have units and there is no intuitive understanding of what a change in one diffusion unit means for evolution. Is this a short or long time (the axes are a measure of time, correct)? Is it a relative or absolute measure? What really adds to the confusion of this section is that the axes are supposed to somehow represent dimensions in sequence space that are hard to move along, yet there is also the connectivity of the network itself drawn in the figures. At times, this connectivity is explicitly referred to and invoked in evolutionary explanations (e.g. wormholes), but shouldn't the existence of such connections already be captured by the diffusion axes? I guess what I'm struggling with is how to reconcile points that are far apart on the axes, but connected by what looks like a single genotype in between.

In the revised manuscript, we have substantially shortened and simplified this section. In the original manuscript, the information concerning the axis units, units used to measure time, and a rigorous definition of the diffusion axes were all included in the Methods section, but we have now added further verbal description to the main text and figure caption so as to avoid any confusion. Specifically, we now clarify in the main text and in the figure legend that the Diffusion Axes have units of square root of time (so that squared distances have units of time), and time is scaled so that individual nucleotide mutations occur at rate 1 (time is scaled to the waiting time for specific mutations to appear).

In this comment, the reviewer is struggling with a real, but counter-intuitive, phenomenon that occurs in high-dimensional sequence space. In particular, it is completely consistent that two genotypes may typically be connected only by very long evolutionary paths (and thus be plotted far apart in the visualization) even if there exists a short direct high fitness path between them. This is because in the high–dimensional context of sequence space there may be orders of magnitude more long winding paths than there are short simple paths. Addressing this reality is one of the key motivations for this section, which provides an important complement to our analysis of simpler metrics of evolutionary accessibility. In order to help clarify this issue further for the reader, we now provide explicit calculations for the fraction of time a population would use the rare but direct paths (wormholes), showing in one case that the direct path would only be used approximately 1% of the time whereas in another case the direct path would be used only 0.002% of the time.

A few suggestions may help. First the figure text is quite small. It would also be helpful to label in the figure which sites are mutated in the entire data set to help line up with the text, i.e. the text uses site numbers like 41 and 54, while the figure has sites 1-4. A clearer labeling of the regions 1,2, and 3 would also help. Finally, I like being able to see specific examples in this section, but there is a lack of quantitative understanding. In the end, the conclusion seems vague about some sort of network structure being important, but what that structure is, how I can see that in the data, and how that conclusion is robust with only a couple of examples is unclear.

We thank the reviewer for the helpful suggestions. We have increased the fontsize of the labels, labeled the amino acid positions, and colored the genotype networks with the fittest 1% genotypes by the corresponding functional region. We have also added a new supplementary Figure S6, which shows visualization of the GB1 landscape at the amino acid level (i.e., assuming any amino acid can mutate into any other), as well as sequence logos of the three functional regions. In addition, we have supplemented our qualitative analysis of network structure with several specific calculations of Markov chain statistics (relaxation times, and probabilities of taking one set of paths versus another), and have clarified that the main purpose of this section is to illustrate the diversity of fitness landscape structures different rewirings of the genetic code can generate.

Minor concerns:

1. There are no methods details on how the rewired genetic codes were generated. This information can be found in previous papers that are cited. However, since this is key to the current work, it would be helpful to have more details on exactly what properties of the standard genetic code are maintained. For example, more explicit details on how the split serine codons, stop codons, and the fact that some amino acids have more codons than others are handled.

We have added the requested methodological details for all code rewiring schemes, including a new scheme added in revision (lines 711-722, Supplementary Notes S4 and S5).

2. I couldn't find the results for the two ParD datasets for the equivalent analysis on lines 227-230.

In our effort to shorten the manuscript, we have removed these results from the main text. They can be found on Zenodo.

3. In section 2.4, I would suggest not mentioning the weak mutation adaptive walks until the very end of the section. Essentially, pulling some of the information out of the beginning of this section to the end would make it easier to follow.

We have followed this suggestion. We now present the weak mutation adaptive walks in Supplementary Note S8, which is referenced at the end of section 2.3.

4. In section 2.5.2. Line 506. Disconnected networks with holes diminish evolvability compared to what exactly? An ideal case without these? Does such a situation exist? I see how disconnection works, but holes are only holes in one dimension of sequence space, there may easily be alternative routes that are easy to pursue. In addition, disconnection and holes can be a result of thresholding behavior. In this sense it is another form of epistasis that may be artificial as the thresholds are based on a percentage cutoff and not on actual function.

The remark about diminished evolvability indeed compared the grids with “holes” to an ideal case without these (the reviewer is correct in their intuition that such a perfect grid is unlikely to appear in empirical landscapes; however, one could easily construct an artificial landscape with this property). The reviewer also rightly points out that such holes might be easily bypassed in higher dimensions; however, such bypasses would inevitably be longer than the direct routes. Lastly, it is true the visualizations restricted to 1% fittest genotypes introduce an artificial threshold. However, we do believe there is merit in showing these restricted landscapes, as they provide a compelling illustration of the huge influence the genetic code has on the structure of fitness landscapes.

To forego any confusion, the discussion of holes has been removed from the main text and now only appears in Supplementary Note S9.2. We have also removed any discussion of the relationship of holes to evolvability.

Reviewer #3:

A referee report on Protein evolvability under rewired genetic code

This paper by Rozhoňová et al. studies the effect of the Standard Genetics Code (SGC) on protein evolvability. The work belongs to a sizable literature that uses theoretical investigations to understand the SGC and its evolution. The main question in the field, is why is there (almost exclusively) one genetic code, the SGC, and not a different hypothetical one, or why not multiple alternative codes for different families of organism. There are basically two possible answers to this question, either that the SGC is "among the best possible codes", or that it's a good one, and its selection among equally good or better codes is a "frozen accident". A landmark paper in the field from Hurst and colleagues suggested that the genetic code is "one in a million" in that it tends to be better than any random set of a million alternatives (yet perhaps not the best among larges sets of alternatives) in terms of its robustness to mutations. The current work walks along same lines but adds a new interesting component: it compares the SGD against a set of 100,000, generated by a proper randomization process (in fact two, one that preserves block structure and one that doesn't), but unlike previous works it computes how each code affects evolvability of three proteins for which a fitness landscape was measured empirically in exhaustion in a small number of positions.

The current work splits the analysis into to either small mutational steps or to long trajectories of evolutionary simulations to examine how each random code, along with the SGC, would allow evolvability. Several measures of evolvability are used (common from the literature) such as number of fitness peeks reached, epistasis and peek accessibility, along with network connectivity (which was new to me) to rank the SGC relative to random alternatives. The conclusions are that the SGC is better than many other random codes in promoting evolvability of proteins, at least on the present 3 fitness landscapes. It is relatively robust and it facilitates predictable adaptive evolution.

This is an interesting paper describing a research which was done very intelligently. The paper is well-written, and all methodological aspects are sounds. In terms of methodological means to study the genetic code there's impressive novelty, not only because it uses wisely empirical fitness landscapes, but because it also deals elegantly with their internal structure and biases (e.g. by correcting for differences in terms of number of mRNAs that correspond to a peak etc). However the biological results are not ground breaking in my mind. It was already appreciated that the code is robust, and now we see that such robustness is associated with evolvability. But the correspondence between robustness and evolvability remains unclear, the correlation between these two properties are rather weak (only 1-2% variance is explained among codes by the observed level of correlation), and all conclusions are based on 3 proteins and they are not consistent among them. I believe that a lot more is needed for a PloS Biology, and would suggest that otherwise the contribution might fit PLoS Computational Biology better.

We thank the reviewer for their feedback on our work, which was mostly positive, but also included some important critical points, specifically regarding the strength of correlation between robustness and evolvability and the number of proteins analyzed.

We agree that the correlations reported in the main text are weak, and we are forthcoming about this in our writing. However, the correlations are mainly consistent across proteins, in that more robust codes tend to produce smoother fitness landscapes. This observation therefore clarifies the relationship between code robustness and protein evolvability in that it resolves previous conflicting reports about whether these two properties are antagonistic or synergistic (e.g. Firnberg and Ostermeier, NAR, 2013 vs. Pines et al., mBio, 2017). For example, our observation of a weak positive correlation between code robustness and protein evolvability is in direct contrast to the suggestion by Pines et al. (mBio, 2017) that these are antagonistic properties. Moreover, the correlations we report in the main text are conservative, with considerably stronger correlations observed under alternative definitions of code robustness (see our response to your point 1 below and to Reviewer 2’s major point 1) and in our analysis of the Ostrov codes.

Regarding the number of proteins analyzed, we agree that including more proteins would improve the generality of our findings. However, as detailed below, other publicly-available combinatorially complete (i.e., 20^L) data sets have shortcomings that prohibit their inclusion in our study. The two exceptions are data sets describing the binding profile of ParB to two distinct DNA sequences, and a data set released on November 24th describing the fitness effects of mutations to dihydrofolate reductase in the presence of the antibiotic trimethoprim. We have now included these data

sets in our study, doubling the number of fitness landscapes from three to six. Please see our response to your second comment below.

Comments

1. The abstract states that the SGC mitigates errors, but what I actually see is that it does so very moderately. Perhaps the biggest result here is more negative than positive - while Hurst introduced the "1 in a million" statement, what we see here, across the board, is that even among 100,000 random codes, there are many that are better than the SGD. In one of the analyses, for example, the SGC is ranked only at the top 5.48%, i.e. "one in ~20"! maybe there biggest conclusion is that it's not that great after all? The paper's question is not dependent on the SGD's quality… It could be that the paper's merit is actually in showing the mediocracy of the SGD. But the authors must be clear about that, either correct their statement on the superiority of the code, or make that claim defendable.

We would like to first point out that it has long been recognized that the “one in a million” result does not hold for all definitions of mutational robustness (Haig & Hurst, 1991), so we do not wish to emphasize this further in our manuscript. However, we agree with the Reviewer that with respect to protein evolvability, our message regarding the (non)exceptionality of the standard genetic code needed clarification. We have revised the text carefully to avoid any confusion.

Please see also our response to Reviewer 2’s major point 1, which is related to your concern.

2. All results are based on just 3 proteins whose fitness landscape has been mapped exhaustively. Are these the only ones available for this analysis? I'd be surprised. Of course there are many other proteins whose fitness landscape was mapped but far from exhaustively. These are not ideal for the current study, but I'd guess that a lot could be done with them, here too. For example, the GFP fitness landscape (PMID: 27193686) was mapped only locally, i.e. in the vicinity of the wild-type, but why can't it be used here? I could imagine that the SGD and each of its random alternatives could each be scored on their ability to allow optimization on this GFP landscape, or am I wrong on this? It's far from ideal that the landscape is not exhaustively mapped, but perhaps with clever algorithms, even such partially mapped landscapes (of which we already have many) can be studies wrt evolvability as done here. If the authors find ways to study partially mapped landscape then they can put their theory into true scrutiny.

We agree with the reviewer that it would be extremely useful to perform our analyses on more fitness landscapes. However, we have undertaken a thorough literature search for other combinatorially-complete (i.e., 20^L) data sets and, unfortunately, the three landscapes we used (GB1, ParD-ParE2, ParD-ParE3) are the only reliable ones available. The others suffer from at least one of the following limitations:

(1) Low between-replicate reproducibility. This is the case for the data set in Starr et al., Nature, 2017. The authors report R^2=0.62 for functional variants, but only R^2 = 0.11 when non-functional variants are included. They dealt with this issue by categorizing the variants into low-, intermediate- and high-fitness. Such categorical phenotypes are not suitable for our analysis.
(2) The data are not publicly available. Two papers (Raman et al., Cell, 2016; Yoo et al., Nature Communications, 2020) report combinatorially-complete data sets, but the data are not publicly available. We have repeatedly contacted the authors of both papers for access to their data, but to no avail.
(3) Incomplete sampling. We identified two papers (Yoo et al., Nature Communications, 2020,; Jalal et al., Cell Reports, 2020) that report combinatorially-complete data sets, but the data do not, in fact, include measurements for all 20^L sequence variants. For example, Jalal et al. measured the binding affinity of 20^L variants of the bacterial DNA-binding protein ParB to two different DNA sequences, *parS* and *NBS*, but for each sequence, binding affinities were reported for only ~50% of the protein variants.

As reported, the data sets above are not suitable for our analysis. However, because the pipeline of Jalal et al. (Cell Reports, 2020) was designed to enrich for high-binding variants, we reasoned we could include these two data sets in our analysis by assuming that missing variants simply do not bind the corresponding DNA sequence. We therefore repeated all of our analyses on these two additional fitness landscapes, finding qualitatively similar results to those obtained for GB1, ParD-ParE2, and ParD-ParE3. We also added the landscape from Papkou et al. (Science, 2023),

which was published after our original submission (November 24th, 2023). The number of landscapes included in our analysis has therefore increased from three to six, improving the generality of our findings.

Regarding partially mapped fitness landscapes such as GFP, we agree they can offer insight into the relationship between code robustness and evolvability. Indeed, prior work attests to this (Firnberg and Ostermeier, NAR, 2013; Firnberg et al., *MBE*, 2014; Pines et al., *mBio*, 2017). However, such analyses are inherently limited to a small mutational radius around a single sequence, and thus focus on one-step adaptation. They are not amenable to studying multi-step adaptation using the landscape-based analyses that are the focus of our paper. Their inclusion would therefore not only be a distraction, it would dilute the novelty of our work.

For example: At the end of 2.2.1 we get to realize that while SGC is very good wrt GB1, it's mediocre wrt to the 2 other proteins (32.6% and 57%). What do the authors make out of this?

As stated at the end of Section 2.2.1, this illustrates that the influence of a genetic code on protein evolvability is protein-specific.

Another example: The linear model (line 383) is a clever attempt to connect between size of basin and peak height. But the beta1 values thus obtained correlate very weakly with robustness of the various codes. More generally throughout the paper, the Pearson (I supposed all Rs are Pearson) are between 0.1-0.2, i.e. they explain 1-2% of the variance only. While P-value are always significant, and that's nice, they come from the fact that they have examined a large number of random codes. So with modesty, one must admit that only a fraction of the variance is explained here.

We have clarified in the text that these are Pearson correlations. Regarding their strength, this is caused by our use of an aggregate measure of code robustness, which, in most cases, is not a precise description of which amino acid substitutions are conservative in the context of a particular protein, but rather a general description of conservativeness derived from analyses of many proteins. This is now explicitly addressed in Supplementary Note S3. Please see also our reply to Reviewer 2's major point 1.

Minor comments

1. are the three properties in fig 2 (# of adaptive peaks, sign epistasis and accessibility) correlated with one another? I'd imagine that the first two would be positively correlated, and each negatively with the latter. I'd encourage the authors to plot each pair of these properties against the other across 100,001 codes (the random and SGC) to check for this. If correlations are very strong (or very strongly negative) then we are actually seeing 3 similar graphs that are dependent on one another.

The reviewer is correct that these measures are correlated with one another, as has been pointed out in prior work (Szendro et al. *J. Stat. Mech.*, 2013). Nonetheless, we believe there is merit in reporting all of them, because they illustrate different aspects of landscape topography. Specifically, measures of pairwise epistasis illustrate local topographical properties, whereas measures like the number and mutational accessibility of adaptive peaks illustrate global properties. In response to this comment, we have added Supplementary Table S1 and the following text:

"Whereas these three measures are all correlated with one another (Szendro et al., 2013; Supp. Tab. S1), they illustrate different aspects of landscape topography, ranging from local (pairwise epistasis) to global (number and accessibility of adaptive peaks)."

2. the authors stress "predictability" of the SGD as an attribute that may explain its uniqueness. It's very hard to imagine that evolution would "care about" predictability, and that this attribute would matter for the selectin among alternative codes. So I suggest that authors either explain this point better, or perhaps agree that predictability is good for us human scientists but it could not have been in consideration during evolution. It is possible to claim that for protein engineering this is a useful property.

We agree that predictability is itself unlikely to be an adaptive feature, but rather a byproduct of other features, such as code robustness. We emphasize that we never stated or even suggested that predictability is an adaptive feature. Indeed, we avoided any selective arguments of code evolution in our entire manuscript. We merely pointed out that predictability is associated with code robustness, and is thus a byproduct of whatever other forces shaped the standard genetic code. In response to this comment, and in our effort to shorten the manuscript, we have heavily deemphasized predictability throughout the main text.

3. in section 2.2.2 the authors, I think, have not commented on how SGC performs on epistasis score

These values, for all 6 data sets, are reported in Supp. Table S3.

4. 2.2.4 why not show the results of codon re-wired codes on the same type of plot as in figure 2? But frankly, this type of rewired codes are chemically un-realistic, since they suggest that one nucleotide or three nucleotide changes in a codon have same effect. I'd suggest they at least comment on this, but I'd actually remove this rewiring schemen

We agree with the reviewer that the random codon assignment scheme is rather unrealistic. We included it because (1) we felt it was important to check that our results are not dependent on the choice of randomization scheme (see Rozhonova and Payne, MBE, 2021), and because (2) it is probably the second most widely used rewiring scheme after amino acid permutation (e.g., Alff-Steinberger, PNAS, 1969; Caporaso et al., J Mol Evol, 2005; Tripathi and Deem, J Mol Evol, 2018). In addition, it has recently been shown (McFeely et al., Nature Communications, 2023) that using hyperaccurate ribosome mutants together with unmodified tRNAs, it is possible to encode up to 3 different amino acids in one codon block, greatly increasing the coding capacity of the genetic code. We thus believe that there is merit to showing these results, as they might actually be relevant for genetic code engineering.

This rewiring scheme is now presented only in the supplements (Supplementary Note S5). We have also added an explicit statement about these codes being rather unrealistic as possible alternatives to the standard genetic code:

These differ from the codes generated using amino acid permutation by lacking the synonymous codon block structure of the standard genetic code, making them less realistic as possible alternatives to the standard genetic code than the amino acid permutation codes.

5. In 2.3. they check which amino acid scales are important. I found this section not profound and lack in insight. Unless for example it were the case that ParD3 has a high alpha-helical content, and GB1 - beta strand content. If not, I'd consider removal of this or to substantiate with some stats assurance that this is not trivial.

We have moved these results to Supplementary Note S2, where we also briefly discuss their connection to the structural properties of proteins:

For GB1, the statistically significant properties are enriched in beta-sheet propensity indices and, less consistently across the different landscape ruggedness measures, in alpha-helix propensity and hydrophobicity indices (Supp. Tab. S14). The importance of the preservation of hydrophobicity is consistent with three of the four residues (V39, G41, V54) being located in the protein core (Nisthal et al., 2019); however, the consistent significance of beta-sheet propensity indices is somewhat surprising, as only one of the four residues is located in a beta sheet (Supp. Fig. S1). Similarly, the properties that are significant for the DHFR landscape are enriched in hydrophobicity indices, likely as a result of the fact that most peaks have D or E in their second position, which are the two most hydrophobic amino acids. The properties that are significant for the two ParD3 landscapes, as well as the ParB-parS landscape, in contrast, are consistently enriched in alpha-helix propensity indices (Supp. Tab. S14), which is consistent with both proteins being mostly helical (Supp. Fig. S1).

6. The authors have simulated evolution of a greedy search or by strong selection week mutation. There's also the regime of weak selection strong mutation in the literature. Can they justify which is more relevant and informative here? On which regime the code evolved really?

The strong mutation regime is a good approximation of directed protein evolution experiments, which is an important application of rewired genetic codes. We have therefore chosen to focus on this regime in the main text, and moved the results for the weak mutation regime to the Supplement (also in response to Reviewer 2). The results are qualitatively the same for both regimes.

Regarding the population genetic regime under which the standard genetic code evolved, one can only speculate. The strong mutation regime is a likely candidate, because it is a reasonable assumption that early in code evolution all molecular machinery was far less precise than it is today, resulting in a high mutation rate. However, we wish to emphasize that our paper is not about the evolution of the standard genetic code. The intent of our population genetic simulations is to study how a population of proteins might evolve under a particular genetic code, standard or otherwise.

7. I didn't understand why time to convergence is long in SGC, is that a good property of it? It actually seems bad to me, please comment.

We never stated this is a good property, but rather emphasized the tradeoff between landscape ruggedness and time to convergence (lines 324-327):

We also observe that the average length of the walks tended to be longer under robust codes (Fig. 3C; 4.89 vs. 5.30 steps, on average, for the 1% least and most robust codes, respectively), revealing that the benefit of increased fitness afforded by code robustness comes at the cost of longer evolutionary trajectories to adaptation.

8. Section 2.5. - Clever analyses. Please explain in the main text what are "Diffusion Axes"

Thank you! We have clarified our description of the methods used in this section. Please see our response to Reviewer 2.

Reviewer #4:

The authors of "Protein evolvability under rewired genetic codes" study the space of alternatives to the standard genetic code (SGC) in order to establish whether the SGC has properties that are beneficial to biological evolution. I found this paper interesting and generally sound. Some comments follow below.

One property, robustness, has already been shown to hold for the SGC, but the relationship of robustness and evolvability - much-studied in other contexts, such as the work of Andreas Wagner - is unclear in the case of the genetic code.

The authors use data from three empirical fitness landscapes that provide complete coverage of all amino acid mutations for a handful of key sites. They then employ criteria such as the number of adaptive peaks, epistasis, and global peak accessibility to assess the evolvability of the landscapes for a large number of 'rewired' genetic codes. They find that the SGC produces smooth landscapes that are more evolvable in the sense of the above criteria.

In my view this analysis is missing one important element, namely dimensionality. The dimensionality of the space is low, due to the small number of mutation sites considered in each empirical landscape. Low dimensionality leads to rugged landscapes, and lowers the accessibility of global peaks. (See e.g. Greenbury et al. in Nat Ecol Evol 2022, and Gavrilets on "High-dimensional fitness landscapes and speciation", 2010). It would be nice to see a discussion of this aspect, especially as the authors rely on these metrics to define evolvability.

Their conclusions may still hold for higher-dimensional landscapes, i.e. if their landscapes included a larger number of key sites, e.g. if the number of adaptive peaks decreases across codes but the SGC still has fewer peaks than the rewired codes.

Given that studying higher dimensional landscapes is challenging in this context, perhaps the authors can explore how their results change if they lower the dimensionality even further by excluding one or more sites? Do the landscapes become more rugged and do their conclusions nevertheless still hold?

If the aspect of dimensionality can be addressed I see no reason why this paper wouldn't be publishable in PLOS Biology.

We thank the reviewer for their positive assessment of our work and for raising the issue of landscape dimensionality. We have followed their suggestion by lowering the dimensionality of the GB1 landscape, which has the most variable protein sites of our six data sets. More specifically, we generated all 80 landscapes with dimensionality $L = 3$ and randomly sampled 100 landscapes with dimensionality $L = 2$, and repeated our landscape analyses and population genetic simulations using them. While there is an expected increase in variance among landscapes, on average our main conclusions hold in these lower-dimensional landscapes: Robust genetic codes render smooth adaptive landscapes that promote protein evolvability. These analyses are presented in Supp. Note S6.

Moreover, this analysis revealed that, on average, the strength of the correlations between code robustness and our various measures of landscape ruggedness increase as landscape dimensionality increases, suggesting that our main findings will likely be reinforced should empirical landscapes of higher dimensionality be characterized in the future. We have added the following text addressing this (lines 610-615):

Second, all six landscapes pertain to just a small number of sites within a larger protein, so it is not possible to understand how sequence variants at these sites interact with other sites in the protein (Gavrilets, 2010; Greenbury et al., 2022). However, our ablation analysis of the GB1 landscape suggests that the average strength of the correlation between robustness and evolvability increases as the number of protein sites increases from 2 to 4 (Supp. Note S6).
