# Supplementary material for: Robust genetic codes enhance protein evolvability
Source: PLoS Biol. 2024 May 16;22(5):e3002594. doi: 10.1371/journal.pbio.3002594 (PMC11098591; doi:10.1371/journal.pbio.3002594)
Supplement: Attachment — Submitted filename: responses.pdf [file pbio.3002594.s003.pdf]

Reviewer #1:

Apologies for a slow response here as commitments became rather overwhelming in the past couple of weeks.

I fully support publication of this paper - I think the points raised by reviewers are valid, especially as summarized by the editor. I think that the authors have addressed these points, and that the paper is publishable. I do have very minor comments (really the suggestion to add ~2 important sentences, that continue/strengthen the response to these comments.

Thank you for the positive assessment of our work!

Editor comment 1: "Shorten the manuscript..."
Reviewer comment from me: The significant reduction definitely makes a more readable paper to this reader: thank you! I do regret that lots of important information is now in the supplementary data - but that is exactly what was asked for. So in that context, I probably haven't truly let go of my original impression that there were two papers here: one (lower impact factor) that sets up all the assumptions and methods, with checks and validations; then a second paper focusing on the story (results) presented here. I say that because the remainder of my comments below really just ask the authors if they could be careful to highlight specific elements of this important supplementary information for careful readers - this background material is I think crucial to a healthy and accurate reading of the work as a whole.

Editor comment 2: "Address the impact of the method used to calculate robustness and clarify some of the methods that are missing or unclear (as suggested by reviewer 2). Which physicochemical properties were used, and which ones were most important?"

Reviewer comment from me: Again, I am 100% in support of the editor's redux of the reviewer's point - very well spotted by the reviewer, and the more I dug into this point the more interested I was by the (apparent) answers. I now perceive that within the discussion, under the paragraph "several caveats…", it merits a sentence that highlights the nature of the simplification and where it can be read about in much greater detail in supplementary material. If I understand correctly, then robustness of the code, measured around the concept of "fraction of point mutations which do NOT cause a change in amino acid meaning" also simplifies the 20 amino acid meanings into groups which are not truly synonymous (from the perspecitve of traditional molecular evolution) - this simpliification scheme is inherited from Pines et al. [22] (Supp. Fig. 725 S3), right? Fascinating, but definitely something a naive reader needs to bear in mind thinking about the results here.

Thank you for pointing this out. We have added the following text to the discussion (lines 623-632):

Third, in the main text, we defined code robustness using a discrete categorization of amino acids based on their physicochemical properties (Supp. Fig. S3). A mutation between two amino acids belonging to the same physicochemical group was considered `synonymous'. While it is well known that some amino acid pairs are more exchangeable than others (Dayhoff1978, Henikoff1992, Yampolsky2005), it is a simplification to assume that these amino acids are entirely equivalent. To overcome this limitation, we additionally considered definitions of code robustness based on a large number of diverse physicochemical properties, one at a time (Supp. Note S2), and on a custom amino acid similarity score specific to each of the six data sets (Supp. Note S3), with qualitatively the same results.

Editor comment (3): "Clearly discuss the very weak correlations and different outcomes for the different proteins, and resulting biological implications, as indicated by reviewer 3. I think these issues do reduce the novelty and impact of the results; at the very least they should be clearly discussed. The low dimensionality of the dataset (reviewer 4) is similarly an important concern that should be discussed.

R1: Once again I do see the editor/reviewer's point and see/appreciate the steps taken by the authors. Once again, however, I would encourage the authors to add a specific sentence to the Discussion (probably paragraph beginning "There are several caveats to the results presented here…"?) reminding readers that results vary for the 4 proteins, and directing them specifically to the supplementary information where this can be considered in details (e.g. S2 Analysis of physicochemical properties from the Aaindex?)

We have changed the first half of the suggested paragraph to (lines 604-616):

There are several caveats to the results presented here. First, because there are so few combinatorially-complete data sets measuring a quantitative phenotype for all 20^L protein variants, our conclusions are based on only six empirical adaptive landscapes. Moreover, the six landscapes differ in the strength of the relationship between code robustness and landscape ruggedness (see, e.g., Supp. Tab. S2) and, in some cases, even in the sign of the trend. We believe this results from the low dimensionality of our landscapes, in that the landscapes are largely influenced by idiosyncracies specific to a single protein site (Gavrilets2010, Greenbury2022). For example, we observe that the fitness reached by greedy walks in the DHFR landscape is mostly determined by whether the genetic code allows a mutation from cysteine to aspartate or glutamate (Supp. Note S7.1). Indeed, our ablation analysis of the GB1 landscape suggests that the average strength of the correlation between robustness and evolvability increases as the number of protein sites increases from 2 to 4 (Supp. Note S6).

Reviewer #2:

I'm satisfied with the author's responses to the reviews. The paper is a great addition to the field.

Thank you!

Reviewer #3:
[identifies himself as Yitzhak Pilpel]

The authors have revised the paper according to comments made by the 3 referees. Obviously the other referees are more positive than myself.

I do appreciate very much their thought and response to my comments. Adding three more proteins is a good addition to their test set. I remain under impressed due to the two main caveats I saw in the first submission: still very few proteins examined. I still believe that they could have tested proteins whose mapping was incomplete (that don't meet the "20^L" criterion), that would have been a methodological achievement by itself, and could allow them to examine many more proteins. It is still the case that the results are very very moderate in magnitude, and still a very small % of the variance is explained. Their response to my question of generality "As stated at the end of Section 2.2.1, this illustrates that the influence of a genetic code on protein evolvability is protein-specific" confuses me very much. The whole discussion of the code supporting evolvability could imply some generality, but the end result is lack thereof , with only 6 proteins examined.
But I'm not against publication. The paper and study, as said originally, is very intelligently done, it will contribute to the study of the code and will surely be followed up by others.

Thank you for your comments, and for supporting the publication of our work.

We do believe that the positive correlation between code robustness and evolvability is general, in that, when averaging over many proteins, robust codes lead to increased evolvability. What we mean when we write "the influence of a genetic code on protein evolvability is protein-specific" is that for a particular code it is hard to predict what effect it will have in a particular landscape – as the reviewer correctly notes, the proportion of variance explained by code robustness is fairly low. This is further amplified by our observation that in the six landscapes, different amino acid properties seem to be important for protein function (see Supp. Note S2 and S3). Thus, a code robust in terms of hydrophobicity and/or amino acid volume might promote evolvability in the GB1 landscape to a great extent, but not so much in the ParD landscapes, where alpha-helix propensity is more important. In short, the trends we observe are true when averaged over a large number of codes and

several landscapes; for a particular code and a particular landscape, the behavior may be idiosyncratic. We believe that this is at least partially due to the low dimensionality of the landscapes.

Please see also our response to Reviewer 1’s second comment.

Reviewer #4:

The authors have addressed my concerns. I am happy for this paper to be published in PLOS Biology.

Thank you!
